# Benzofuran–appended 4-aminoquinazoline hybrids as epidermal growth factor receptor tyrosine kinase inhibitors: synthesis, biological evaluation and molecular docking studies

**DOI:** 10.1080/14756366.2018.1510919

**Published:** 2018-10-02

**Authors:** Malose J. Mphahlele, Marole M. Maluleka, Abimbola Aro, Lyndy J. McGaw, Yee Siew Choong

**Affiliations:** a Department of Chemistry, College of Science, Engineering and Technology, University of South Africa, Florida, South Africa;; b Phytomedicine Programme, Department of Paraclinical Sciences, University of Pretoria, Onderstepoort, South Africa;; c Institute for Research in Molecular Medicine (INFORMM), Universiti Sains Malaysia, Minden, Malaysia

**Keywords:** Benzofuran-aminoquinazolines, cytotoxicity, apoptosis, EGFR-TK, molecular docking

## Abstract

A series of 2-arylbenzo[*b*]furan–appended 4-aminoquinazoline hybrids were prepared and evaluated for cytotoxicity in vitro against the human lung cancer (A549), colorectal adenocarcinoma (Caco-2), hepatocellular carcinoma (C3A) and cervical cancer (HeLa) cell lines. Compounds **10d** and **10j** exhibited significant cytotoxicity against the C3A and Caco-2 cell lines and induced apoptosis in these cell lines. Likewise, compounds **10d** and **10e** exhibited significant inhibitory activity towards epidermal growth factor receptor-tyrosine kinase phosphorylation (IC_50_ values of 29.3 nM and 31.1 nM, respectively) against Gefitinib (IC_50_ = 33.1 nM). Molecular docking of compounds **10** into EGFR-TK active site suggests that they bind to the region of EGFR like Gefitinib does.

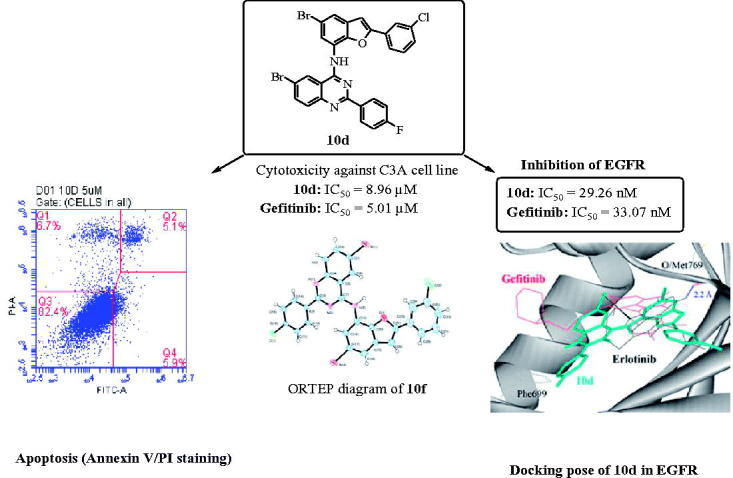

## 1. Introduction

**Figure 1.**
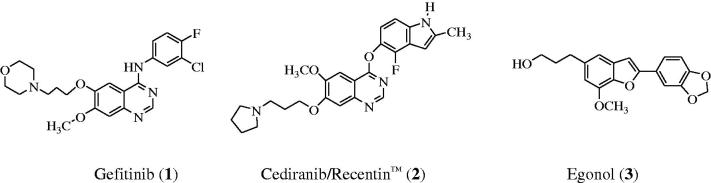
Structure of Gefitinib (**1**), Cediranib (**2**) and Egonol (**3**).

Epidermal growth factor receptor (EGFR) is a tyrosine kinase receptor that has been found to play an essential role in normal cell growth and differentiation and it is also involved in tumor proliferation and survival^1^. This receptor is over expressed in various types of tumors including colon, non-small cell lung, prostate, breast and ovarian cancers^2^. EGFR continues to be an attractive target for the design and development of compounds that can specifically bind to it and inhibit its tyrosine kinase (TK) activity and its signal transduction pathway in cancer cells^3^. The 4-aminoquinazolines have established themselves as selective and effective inhibitors of the epidermal growth factor receptor tyrosine kinase (EGFR-TK) phosphorylation, which results from competitive binding at the ATP site^4^^,^^5^. Gefitinib (**1**) shown in Figure 1, for example, is a selective inhibitor of the EGFR-TK and it inhibits tumor pathogenesis, metastasis and angiogenesis, and also promotes apoptosis^6^. Structure–activity relationship studies for the ability of the 4-anilinoquinazolines to inhibit EGFR-TK activity revealed that both of the quinazoline nitrogen atoms are essential for biological activity and the aniline moiety bearing lipophilic substituents such as chloro, bromo and trifluoromethyl group was also important as it occupies the lipophilic pocket^7^. In the design of new drugs, hybridization approach has been found to provide a more general method to obtain molecular hybrids with improved biological activities capable of overcoming multi-drug resistance. This strategy has been employed before in the synthesis of an indole-ether quinazoline hybrid, Cediranib/Recentin™ (**2**) shown in Figure 1, which is a highly potent, orally bioavailable and selective vascular endothelial growth factor receptor (VEGFR) inhibitor for the treatment of cancer^8^^,^^9^.

**Scheme 1.**
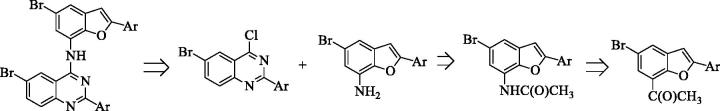
Design of benzofuran-amino quinazolines using molecular hybridization approach.

In our previous investigation we observed that a combination of the 2–(4-fluoro/chlorophenyl) group and a bromine atom at the 6-position of the 4-anilinoquinazoline framework leads to increased antiproliferative activity^10^. Literature review revealed that most of the EGFR-TK inhibitors have a common 4-aminoquinazoline core and only differ in terms of the substituents and side chains. Moreover, the 4-anilinoquinazolines substituted with a bromoaniline group have previously been found to exhibit high affinity and selectivity for EGFR-TK^11^. A bromine atom on C-5 position of a benzofuran ring or the analogous indole moiety has been found to impart significant biological activity of the 2-arylbenzofurans^12^ or 2-arylindoles^13^. These literature precedents encouraged us to replace the phenyl ring of the 4-anilino group of the 2-aryl-6-bromoquinazolines with a 2-aryl-5-bromobenzofuran moiety to comprise benzofuran-appended 4-aminoquinazoline analogs of **2** (Scheme 1). This idea was also inspired by the versatility of the naturally occurring Egonol (**3**) shown in Figure 1 and its derivatives, which have been found to exhibit cytotoxic, anti-inflammatory, anti-asthma, and antioxidant properties^14–16^. The main aim was to evaluate the resultant molecular hybrids for potential antiproliferative properties *in vitro* against a panel of EGFR-positive cancer cell lines^2^, namely, the human lung cancer (A549), colorectal adenocarcinoma (Caco-2) and the hepatocellular carcinoma (C3A; HepG2/C3A) cell lines as well as the cervical cancer (HeLa) cell line with relatively low EGFR expression^17^. The ability of the resultant benzofuran-aminoquinazoline hybrids to induce apoptosis and their potential to inhibit EGFR-TK phosphorylation were evaluated experimentally complemented with molecular docking (*in silico*) studies into the ATP binding site of the EGFR.

## 2. Materials and methods

### 2.1. Chemistry

The melting points of the prepared compounds were recorded on a Thermocouple digital melting point apparatus and are uncorrected. Their IR spectra were recorded as powders by using the thin-film method on a Bruker VERTEX 70 FT-IR Spectrometer (Bruker Optics, Billerica, MA, USA) equipped with a diamond ATR (attenuated total reflectance) accessory. The Merck kieselgel 60 (0.063–0.200 mm) (Merck KGaA, Frankfurt, Germany) was used as stationary phase for column chromatography. The ^1^H- and ^13^C-NMR spectra of the prepared compounds were obtained as CDCl_3_ or DMSO-d_6_ solutions using the Agilent 500 MHz NMR spectrometer (Agilent Technologies, Oxford, UK) and the chemical shifts are quoted relative to the TMS peak. The low- and high-resolution mass spectra were recorded at the University of Stellenbosch using a Waters Synapt G2 Quadrupole Time-of-flight mass spectrometer (Waters Corp., Milford, MA, USA) at an ionization potential of 70 eV. The synthesis and analytical data of the 2–(4-halogenophenyl)-4-chloroquinazolines **9a** and **9 b** have been described before^18^. Copies of ^1^H- and ^13^C-NMR spectra of compounds **6a**–**e**, **7a**–**e**, **8a**–**e**, **10a**–**j** are included as Supplementary Material.

### 2.2. Synthesis of 5-bromo-2-hydroxy-3-iodoacetophenone (5)

#### 5-Bromo-2-hydroxyacetophenone

A stirred solution of hydroxyacetophenone (5.26 g, 38.7 mmol) in acetic acid (400 ml) was treated with *N*-bromosuccinimide (18.02 g, 38.7 mmol) at room temperature (RT). The mixture was stirred under reflux for 1.5 h and then quenched with an ice-cold water. The resulting precipitate was filtered and recrystallized to afford 5-bromo-2-hydroxyacetophenone (4.92 g, 59%); mp. 74–75 °C (hexane); ν_max_ (ATR) 491, 516, 624, 735, 879, 1081, 1195, 1285, 1346, 1473, 1559, 1640, 3280 cm^−1^; ^1^H NMR (CDCl_3_) 2.63 (3H, s, CH_3_), 6.87 (1H, d, *J* = 8.5 Hz, H-3), 7.53 (1H, dd, *J* 2.4 and 8.5 Hz, H-4), 7.81 (1H, d, *J* = 2.4 Hz, H-6), 12.14 (1H, s, OH); ^13^C NMR (CDCl_3_) 26.6, 87.6, 106.7, 118.9, 134.7, 145.7, 148.5, 203.0; HRMS (ES): MH^+^, found. C_8_H_8_O_2_^79^Br^+^ requires: 215.9609.

#### 5-Bromo-2-hydroxy-3-iodoacetophenone (**5**)

A stirred solution of 5-bromo-2-hydroxyacetophenone (3.23 g, 15.0 mmol) and *N*-iodosuccinimide (5.78 g, 18.0 mmol) in acetic acid (400 ml) was refluxed for 2 h and then quenched with an ice-cold water. The resulting precipitate was filtered and recrystallized from EtOH to afford **5** as a brown solid (4.78 g, 93%), mp. 111–112 °C (Lit^19^. 104–105 °C); ν_max_ (ATR) 441, 539, 670, 779, 862, 968, 1077, 1190, 1233, 1362, 1420, 1581, 1650, 3259 cm^−1^; ^1^H NMR (CDCl_3_) 2.65 (3H, s, CH_3_), 7.83 (1H, d, *J* = 2.1 Hz, H-4), 8.04 (1H, d, *J* = 2.1 Hz, H-6), 13.07 (1H, s, OH); ^13^C NMR (CDCl_3_) 26.4, 87.6, 111.1, 120.2, 133.1, 147.1, 160.2, 203.2; HRMS (ES): MH^+^, found. C_8_H_7_O_2_^79^BrI^+^ requires: 341.8575.

### 2.3. Typical procedure for the synthesis of benzofuran derivatives 6a–e

To a three necked round-bottom flask equipped with a stirrer bar, condenser and rubber septa was added in sequence compound **5** (1 mmol), PdCl_2_(PPh_3_)_2_ (5 mmol % of **5**), CuI (10 mmol % of **5**), Cs_2_CO_3_ (1.2 equiv. of **5**) and 10:1 dioxane-water (7.0 ml/mmol of **5**). The mixture was purged with nitrogen gas for 20 min. and a balloon filled with nitrogen gas was connected to the top of the condenser. Phenylacetylene (1.2 equiv. of **5**) was added via a syringe and the mixture was heated at 80 °C for 2 h and then quenched with an ice-cold water. The product was extracted with chloroform and the combined organic layers were washed with brine and dried over MgSO_4_. The salt was filtered off and the solvent was evaporated under reduced pressure on a rotary evaporator. The residue was purified by column chromatography on silica gel using 30% toluene-hexane mixture as an eluent to afford **6a**. The following compounds were prepared in this fashion.

#### 2.3.1. 1–(5-Bromo-2-phenylbenzofuran-7-yl) ethanone (6a)

Solid (0.69 g, 73%), *R_f_* = 0.33, mp. 130–133 °C; ν_max_ (ATR) 684, 790, 884, 989, 1095, 1179, 1279, 1360, 1490, 1567, 1586, 1677, 3064 cm^−1^; δ_H_ (CDCl_3_) 2.95 (3H, s, CH_3_), 7.03 (1H, s, H-3), 7.42 (2H, d, *J* = 7.0 Hz, Ar), 7.48 (1H, t, *J* = 7.5 Hz, Ar), 7.85 (2H, d, *J* = 8.0 Hz, Ar), 7.98 (1H, d, *J* = 1.5 Hz, H-4), 7.99 (1H, d, *J* = 1.5 Hz, H-6); δ_C_ (CDCl_3_) 31.0, 100.5, 116.4, 122.9, 125.2, 127.5, 128.1, 128.6, 129.0, 129.5, 132.8, 152.1, 157.8, 194.6; HRMS (ES): found 316.9743. C_16_H_12_O_2_^79^Br^+^ requires 316.9726. *Anal* calcd for C_16_H_11_O_2_Br: C, 60.98; H, 3.58. Found: C, 60.96; H, 3.44.

#### 2.3.2. 1–(5-Bromo-2–(3-fluorophenyl)benzofuran-7-yl)ethanone (6b)

Solid (0.73 g, 76%), *R_f_* = 0.32, mp. 161–164 °C (ATR) 667, 781, 878, 987, 1081, 1163, 1280, 1365, 1488, 1556, 1686; δ_H_ (CDCl_3_) 2.94 (3H, s, CH_3_), 7.06 (1H, s, H-3), 7.12 (1H, t, *J* = 8.5 Hz, Ar), 7.40 (2H, dd, *J* = 8.5 and 2.0 Hz, Ar), 7.64 (1H, d, *J* = 8.5 Hz, Ar), 7.89 (1H, d, *J* 2.0 Hz, H-4), 8.00 (1H, d, *J* 2.0 Hz, H-6); δ_C_ (CDCl_3_) 30.9, 101.6, 112.1 (d, ^2^*J*_CF_*=*23.6 Hz) 116.4 (d, ^2^*J*_CF_ = 21.9 Hz), 116.5, 120.9 (d, ^4^*J*_CF_ = 2.8 Hz), 123.1, 127.5, 128.1, 128.4, 130.7 (d, ^3^*J*_CF_ = 7.5 Hz), 132.5, 152.1, 156.4 (d, ^4^*J*_CF_ = 2.9 Hz), 163.1 (d, ^1^*J*_CF_ = 249.4 Hz) 194.4; HRMS (ES): found 332.9828. C_16_H_11_O_2_F^79^Br^+^ requires 332.9826. *Anal* calcd for C_16_H_10_O_2_FBr: C, 57.68; H, 3.03. Found: C, 57.50; H, 2.99.

#### 2.3.3. 1–(5-Bromo-2–(4-fluorophenyl)benzofuran-7-yl)ethanone (6c)

Solid (0.82 g, 76%), *R_f_* = 0.31, mp. 178–180 °C; ν_max_ (ATR) 668, 784, 898, 987, 1029, 1166, 1280, 1365, 1488, 1555, 1682 cm^−1^; δ_H_ (CDCl_3_) 2.93 (3H, s, −CH_3_), 6.98 (1H, s, H-3), 7.18 (2H, t, *J* = 8.5 Hz, H-2',6'), 7.83 (2H, t, *J* = 8.7 Hz, H-3',5') 7.86 (1H, d, *J* 1.8 Hz, H-4), 7.97 (1H, d, *J* 1.8 Hz, H-6); δ_C_ (CDCl_3_) 30.9, 100.1, 116,3 (d, ^2^*J*_CF_ = 22.8 Hz), 125.6, 127.1, 127.6, 128.1, 128.5 (d, ^3^*J*_CF_ = 12.2 Hz), 132.2 (d, ^4^*J*_CF_ = 10.3 Hz), 132.8, 151.9, 156.9, 163.4 (d, ^1^*J*_CF_ = 249.4 Hz) 194.5; HRMS (ES): found 332.9848. C_16_H_11_O_2_F^79^Br^+^ requires 333.9828. *Anal* calcd for C_16_H_10_O_2_ClBr: C, 57.68; H, 3.03. Found: C, 57.63; H, 3.01.

#### 2.3.4. 1–(5-Bromo-2–(3-chlorophenyl)benzofuran-7-yl)ethanone (6d)

Solid (0.76 g, 74%), *R_f_* = 0.28, mp. 139–141 °C; ν_max_ (ATR) 682, 788, 882, 1078, 1144, 1275, 1359, 1474, 1561, 1587, 1675, 3081 cm^−1^; δ_H_ (CDCl_3_) 2.94 (3H, s, CH_3_), 7.07 (1H, s, H-3), 7.41 (2H, dd, *J* = 1.5 and 8.0 Hz, Ar), 7.78 (1H, d, *J* = 8.0 Hz, Ar), 7.83 (1H, d, *J* = 2.0 Hz, H-4), 7.90 (1H, d, *J* = 1.5 Hz, Ar), 7.97 (1H, d, *J* = 2.0 Hz, H-6); δ_C_ (CDCl_3_) 30.9, 101.6, 116.6, 123.1, 123.2, 125.2, 128.1, 128.4, 129.4, 130.4, 130.9, 132.5, 135.1, 152.1, 156.3, 194.4; HRMS (ES): found 348.9634. C_16_H_11_O_2_^35^Cl^79^Br^+^ requires 348.9631. *Anal* calcd for C_16_H_10_O_43_ClBr: C, 54.97; H, 2.88. Found: C, 54.64; H, 2.95.

#### 2.3.5. 1–(5-Bromo-2–(4-(trifluoromethoxy)phenyl)benzofuran-7-yl)ethanone (6e)

Solid (0.93 g, 77%), *R_f_* = 0.35, mp. 170–173 °C; ν_max_ (ATR) 666, 746, 854, 987, 1033, 1199, 1267, 1363, 1405, 1502, 1614, 1676, 3082 cm^−1^; δ_H_ (CDCl_3_) 2.92 (3H, s, CH_3_), 7.03 (1H, s, H-3), 7.34 (2H, d, *J* = 8.1 Hz, Ar), 7.85 (2H, d, *J* = 9.3 Hz, Ar), 7.98 (2H, d, *J* = 1.8 Hz, Ar); δ_C_ (CDCl_3_) 30.8, 101.1, 116.6, 121.5, 123.0, 126.7, 127.7, 127.9, 128.0, 128.3, 130.2 (t, *J* = 25.6 Hz) 132.6, 152.1, 156.4, 194.4; HRMS (ES): 398.9663. found C_17_H_11_O_3_F_3_^79^Br ^+^ requires 398.9745. *Anal* calcd for C_17_H_10_O_3_F_3_Br: C, 51.15; H, 2.53. Found: C, 51.32; H, 2.67.

### 2.4. Typical procedure for the synthesis of oxime derivatives 7a–e

A mixture of **6** (1 equiv.) and hydroxylamine hydrochloride (1.2 equiv.) in pyridine (8.0 ml/mmol of **6**) was heated at 120 °C for 1 h. The mixture was quenched with an ice-cold water and the product was filtered. The resultant precipitate was washed thoroughly with cold hexane and then recrystallized from ethanol to afford **7** as a solid. Compounds **7a**–**e** were prepared in this fashion.

#### 2.4.1. 1–(5-Bromo-2-phenylbenzofuran-7-yl)ethanone oxime (7a)

Solid (0.76 g, 93%), mp. 177–179 °C; ν_max_ (ATR) 748, 777, 874, 1029, 1230, 1366, 1429, 1445, 1562, 1601, 3107, 3200 cm^−1^; δ_H_ (DMSO-d_6_) 2.42 (3H, s, CH_3_), 7.41 (2H, d, *J* = 7.0 Hz, Ar), 7.47 (1H, s, H-3), 7.53 (1H, t, *J* = 8.5 Hz, Ar), 7.58 (2H, d, *J* = 8.5 Hz, Ar), 7.61 (1H, d, *J* = 1.5 Hz, H-4), 7.84 (1H, d, *J* = 1.5 Hz, H-6), 11.5 (1H, s, OH); δ_C_ (DMSO-d_6_) 14.0, 102.2, 117.8, 122.9, 124.7, 125.3, 126.4, 129.2, 129.8, 130.8, 131.8, 151.0, 151.6, 156.9; HRMS (ES): found 330.0031. C_16_H_13_O_2_N^79^Br^+^ requires 330.0051. *Anal* calcd for C_16_H_12_O_2_NBr: C, 58.20; H, 3.66; N, 4.24. Found: C, 58.33; H, 3.71; N, 4.00.

#### 2.4.2. 1–(5-Bromo-2–(3-fluorophenyl)benzofuran-7-yl)ethanone oxime (7b)

Solid (0.57 g, 86%), mp. 199–201 °C; ν_max_ (ATR) 748, 906, 1029, 1154, 1262, 1316, 1453, 1555, 1603, 3108, 3187 cm^−1^; δ_H_ (DMSO-d_6_); 2.38 (3H, s, CH_3_), 7.48 (1H, s, H-3), 7.49 (1H, d, *J* = 8.0 Hz, H-6'), 7.54 (1H, t, *J* 7.5 Hz, H-4'), 7.59 (1H, d, *J* = 7.5 Hz, H-2'), 7.86 (1H, t, *J* = 7.0 Hz, H-5'), 7.88 (1H, d, *J* = 2.0 Hz, H-4), 7.94 (1H, d, *J* = 2.0 Hz, H-6), 11.6 (1H, s, OH); δ_C_ (DMSO-d_6_) 14.0, 103.3, 112.1 (d, ^2^*J*_CF_*=*23.6 Hz) 116.1 (d, ^2^*J*_CF_ = 21.9 Hz), 119.5, 120.9 (d, ^4^*J*_CF_ = 2.8 Hz), 123.9, 127.7, 129.6, 130.8, 131.5 (d, ^3^*J*_CF_ = 7.5 Hz), 132.2, 134.4, 150.7 (d, ^4^*J*_CF_ = 2.9 Hz), 156.5, 163.1 (d, ^1^*J*_CF_ = 249.4 Hz); HRMS (ES): found 348.0028 C_16_H_12_NO_2_F^79^Br^+^ requires 348.0035. *Anal* calcd for C_16_H_11_NO_2_FBr: C, 55.20; H, 3.18; N, 4.02. Found: C, 55.43; H, 3.33; N, 3.99.

#### 2.4.3. 1–(5-Bromo-2–(4-fluorophenyl)benzofuran-7-yl)ethanone oxime (7c)

Solid (0.61 g, 81%), mp. 216–218 °C; *ν*_max_ (ATR) 690, 769, 852, 944, 1029, 1188, 1276, 1339, 1411, 1503, 1574, 1604 3108, 3187 cm^−1^; δ_H_ (DMSO-d_6_) 2.39 (3H, s, CH_3_), 7.36 (2H, t, *J* = 8.7 Hz, H-2',6'), 7.43 (1H, s, H-3), 7.56 (1H, d, *J* = 2.0 Hz, H-4), 7.86 (2H, d, *J* = 2.0 Hz, H-6), 7.97 (2H, t, *J* = 8.7 Hz, H-3',5'), 11.6 (1H, s, OH); δ_C_ (DMSO-d_6_) (125 MHz, CDCl_3_) 14.0, 101.7, 115.9, 116,7 (d, ^2^*J*_CF_ = 21.9 Hz), 123.9, 124.0, 125.2, 126.1, 126.2, 127.7 (d, ^3^*J*_CF_ = 8.5 Hz), 132.5, 150.7 (d, ^4^*J*_CF_ = 4.7 Hz), 156.3, 163.1 (d, ^1^*J*_CF_ = 245.6 Hz), HRMS (ES): found: 348.9934. C_16_H_12_NO_2_F^79^Br^+^ requires 348.9937. *Anal* calcd for C_16_H_11_NO_2_FBr: C, 55.20; H, 3.18; N, 4.02. Found: C, 55.41; H, 3.31; N, 4.00.

#### 2.4.4. 1–(5-Bromo-2–(3-chlorophenyl)benzofuran-7-yl)ethanone oxime (7d)

Solid (0.39 g, 81%), mp. 192–195 °C ν_max_ (ATR) 748, 849, 916, 1006, 1090, 1162, 1278, 1314, 1337, 1429, 1448, 1561, 1604, 3106, 3211 cm^−1^; δ_H_ (DMSO-d_6_) 2.29 (3H, s, CH_3_), 7.47 (1H, s, H-3), 7.58 (1H, dd, *J* = 2.0 and 8.5 Hz, H-6'), 7.58 (1H, t, *J* = 8.5 Hz, H-5'), 7.67 (1H, d, *J* = 2.0 Hz, H-4), 7.85 (1H, d, *J* = 2.0 Hz, H-6), 7.67 (1H, d, *J* = 8.0 Hz, H-4'), 7.98 (1H, d, *J* = 2.0 Hz, H-2), 11.0 (1H, s, OH); δ_C_ (DMSO-d_6_) 14.0, 103.3, 116.1, 123.9, 124.1, 124.3, 124.9, 125.7, 129.6, 131.5, 131.6, 132.2, 134.4, 150.7, 150.8, 155.5; HRMS (ES): found: 364.9722. C_16_H_12_NO_2_^35^Cl^79^Br^+^ requires 364.9641. *Anal* calcd for C_16_H_11_NO_2_ClBr: C, 52.70; H, 3.04; N, 3.84. Found: C, 52.65; H, 3.14; N, 3.88.

#### 2.4.5. 1–(5-Bromo-2–(4-(trifluoromethoxy)phenyl)benzofuran-7-yl)ethanone oxime (7e)

Solid (0.61 g, 78%), mp. 167–169 °C; ν_max_ (ATR) 681, 777, 908, 1029, 1161, 1279, 1337, 1449, 1460, 1510, 1583, 3074, 3208 cm^−1^; δ_H_ (DMSO-d_6_) 2.51 (3H, s, CH_3_), 6.99 (1H, s, H-3), 7.30 (2H, d, *J* = 8.5 Hz, H-3',5'), 7.61 (1H, d, *J* = 1.5 Hz, H-4), 7.72 (1H, d, *J* = 1.5 Hz, H-6), 7.88 (2H, d, *J* = 8.0 Hz, H-2',6'), 8.34 (1H, s, OH); δ_C_ (DMSO-d_6_) 13.6, 101.3, 116.3, 121.3, 121.3, 122.7, 124.3, 126.1, 126.8, 130.1 (t, *J*_CF_ = 25.6 Hz), 133.2, 149.6, 150.9, 153.1, 156.1; HRMS (ES): found 414.9932. C_17_H_12_NO_3_F_3_^79^Br^+^ requires 414.9854. *Anal* calcd for C_17_H_11_NO_3_F_3_Br: C, 49.30; H, 2.68; N, 3.38. Found: C, 49.19; H, 2.59; N, 3.42.

### 2.5. Typical procedure for the one-pot beckmann rearrangement and hydrolysis of the oxime derivatives 7a–e

A stirred mixture of **7** (1 equiv.) and triflic acid (0.4 equiv.) in acetonitrile (10 ml/mmol of **7**) was heated at 80 °C for 4 h. The mixture was cooled to room temperature and quenched with ice-cold water. The resultant precipitate was washed thoroughly with cold hexane and then recrystallized from ethanol to afford compound **6** as a solid. Compounds **8a**–**e** were prepared in this fashion.

#### 2.5.1. 5-Bromo-2-phenylbenzofuran-7-amine (8a)

Solid (0.35 g, 62%), mp. 177–179 °C; ν_max_ (ATR) 736, 915, 1156, 1214, 1309, 1433, 1502, 1572, 1583, 1611, 3351, 3464 cm^−1^; δ_H_ (DMSO-d_6_) 5.70 (2H, s, NH_2_), 7.21 (1H, s, H-3), 7.28 (2H, d, *J* = 8.5 Hz, Ar), 7.41 (2H, d, *J* = 8.0 Hz, Ar), 7.50 (1H, t, *J* = 8.5 Hz, Ar), 7.65 (1H, d, *J* = 1.5 Hz, H-6), 7.84 (1H, d, *J* = 7.5 Hz, Ar); δ_C_ (DMSO-d_6_) 103.3, 111.1, 125.2, 128.7, 129.3, 129.4, 130.1, 133.6, 134.2, 136.2, 145.9, 156.1; HRMS (ES): found 287.9946. C_14_H_11_NO^79^Br^+^ requires 287.9925. *Anal* calcd for C_14_H_10_NOBr: C, 58.36; H, 3.50; N, 4.85. Found: C, 58.68; H, 3.20; N, 4.77.

#### 2.5.2. 5-Bromo-2–(3-fluorophenyl)benzofuran-7-amine (8b)

Solid (0.34 g, 78%), mp. 124–126 °C; ν_max_ (ATR) 721, 765, 834, 1100, 1261, 1309, 1433, 1572, 1583, 1611, 3375, 3482 cm^−1^; δ_H_ (DMSO-d_6_) 5.81 (2H, br s, NH_2_), 6.68 (1H, d, *J* = 1.5 Hz, H-6), 6.95 (1H, d, *J* = 1.5 Hz, H-4), 7.22 (1H, t, *J* 8.0 Hz, H-4'), 7.35 (1H, s, H-3), 7.52 (1H, dd, *J* 1.5 and 7.5 Hz, H-5'), 7.79 (1H, d, *J* = 8.0 Hz, H-6'), 7.82 (1H, s, H-2'); δ_C_ (DMSO-d_6_) 103.5, 110.5, 111,7 (d, ^3^*J*_CF_ = 8.5 Hz), 111.9, 115.9 (d, ^2^*J*_CF_ = 20.9 Hz), 116.7, 121.3 (d, ^4^*J*_CF_ = 2.9 Hz), 130.8, 131.5 (d, ^3^*J*_CF_ = 8.5 Hz), 132.4 (d, ^3^*J*_CF_ = 8.6 Hz), 135.7, 142.1, 154.3 (d, ^4^*J*_CF_ = 2.8 Hz),163.1 (d, ^1^*J*_CF_ = 240.6 Hz); HRMS (ES): found: 305.9852. C_14_H_10_NOF^79^Br^+^ requires 305.9831. *Anal* calcd for C_14_H_9_NOFBr: C, 54.93; H, 2.96; N, 4.58. Found: C, 54.87; H, 3.09; N, 4.72.

#### 2.5.3. 5-Bromo-2–(4-fluorophenyl)benzofuran-7-amine (8c)

Solid (0.39 g, 71%), mp. 121–123 °C; ν_max_ (ATR) 688, 765, 915, 1100, 1214, 1309, 1433, 1502, 1583, 1612, 3370, 3473 cm^−1^; δ_H_ (DMSO-d_6_) 5.75 (2H, brs, NH_2_), 6.66 (1H, d, *J* = 2.0 Hz, H-6), 6.93 (1H, d, *J* = 2.0 Hz, H-4), 7.23 (1H, s, H-3), 7.34 (2H, t, *J* 8.7 Hz, H-3',5'), 7.99 (2H, t, *J* = 8.5 Hz, H-2',6'); δ_C_ (DMSO-d_6_) 102.1, 110.5, 111.4, 116,4 (d, ^2^*J*_CF_ = 21.8 Hz), 116.6, 126.8 (d, ^4^*J*_CF_ = 2.9 Hz) 127.4 (d, ^3^*J*_CF_ = 8.5 Hz), 131.1, 135.6, 142.1, 154.8, 162.7 (d, ^1^*J*_CF_ = 244.7 Hz); HRMS (ES): found 305.9715. C_14_H_10_NOF^79^Br^+^ requires 305.9776. *Anal* calcd for C_14_H_9_NOFBr: C, 54.93; H, 2.96; N, 4.58. Found: C, 54.88; H, 3.07; N, 4.70.

#### 2.5.4. 5-Bromo-2–(3-chlorophenyl)benzofuran-7-amine (8d)

Solid (0.31 g, 65%), mp. 106–108 °C; ν_max_ (ATR) 737, 854, 879, 1126, 1270, 1429, 1593, 1615, 3372, 3482 cm^−1^; δ_H_ (DMSO-d_6_) 5.82 (2H, br s, NH_2_), 6.68 (1H, d, *J* = 2.0 Hz, H-6), 6.94 (1H, d, *J* = 2.0 Hz, H-4) 7.37 (1H, s, H-3), 7.43 (1H, d, *J* = 8.0, H-4'), 7.51 (1H, t, *J* = 7.5 Hz, H-5'), 7.90 (1H, d, *J* = 7.5 Hz, H-6'), 8.05 (1H, d, *J* = 8.0 Hz, H-2'); δ_C_ (DMSO-d_6_) 103.6, 110.6, 111.7, 116.7, 123.7, 124.7, 128.9, 130.8, 131.3, 132.2, 134.4, 135.7, 142.2, 154.0; HRMS (ES): found 321.9556. C_14_H_10_NO^35^Cl^79^Br^+^ requires 321.9536. *Anal* calcd for C_14_H_9_NOClBr: C, 52.13; H, 2.81; N, 4.34. Found: C, 52.31; H, 2.66; N, 4.28.

#### 2.5.5. 5-Bromo-2–(4-(trifluoromethoxy)phenyl)benzofuran-7-amine (8e)

Solid (0.47 g, 85%), mp. 118–119 °C; ν_max_ (ATR) 685, 737, 823, 947, 1044, 1214, 1468, 1568, 1614, 3382, 3484 cm^−1^; δ_H_ (DMSO-d_6_) 5.78 (2H, br s, NH_2_), 6.95 (1H, d, *J* = 2.0 Hz, H-4), 6.68 (1H, d, *J* = 2.0 Hz, H-6), 7.32 (1H, s, H-3), 7.49 (2H, d, *J* = 8.5 Hz, H-3',5'), 8.05 (2H, d, *J* = 8.0 Hz, H-2',6'); δ_C_ (DMSO-d_6_) 101.3, 116.3, 121.3, 122.7, 124.3, 126.1, 126.8, 130.1 (t, *J*_CF_ = 25.6 Hz), 133.2, 149.6, 150.9, 153.1, 156.1; HRMS (ES): found 371.9748. C_15_H_10_NO_2_F_3_^79^Br^+^ requires 371.9769. *Anal* calcd for C_15_H_9_NO_2_F_3_Br: C, 48.41; H, 2.44; N, 3.76. Found: C, 48.14; H, 2.65; N, 3.89.

### 2.6. Typical procedure for the amination of 9a and 9 b with the 7-aminobenzofurans 8a–e to afford 10a–j

A mixture of **8** (1 equiv.), **9** (1 equiv.) and HCl (5% mol equiv. of **9**) in isopropanol (15.5 ml/mmol of **9**) was stirred under reflux for 2 h. After completion of the reaction (tlc monitoring) the mixture was cooled to room temperature and quenched with ice-cold water. The resultant precipitate was washed thoroughly with hot acetonitrile and then recrystallized from DMSO to afford **10** as a solid. Compounds **8a**–**e** were prepared in this fashion. The following compounds were prepared in this fashion:

#### 2.6.1. 6-Bromo-*N*-(5-bromo-2-phenylbenzofuran-7-yl)-2–(4-fluorophenyl)quinazolin-4-amine (10a)

Solid (0.28 g, 75%) mp. > 300 °C; ν_max_ (ATR) 688, 782, 873, 911, 1047, 1161, 1221, 1373, 1515, 1603, 1631, 3082, 3169 cm^−1^; δ_H_ (DMSO-d_6_) 7.29 (2H, t, *J* = 8.5 Hz, Ar), 7.34 (2H, d, *J* = 8.5 Hz, Ar), 7.47 (1H, s, =C–H), 7.78–7.79 (5H, m, Ar), 7.85 (1H, d, *J* = 8.5 Hz, Ar), 8.04 (1H, d, *J* = 8.0 Hz, Ar), 8.11 (2H, d, *J* = 8.5 Hz, Ar), 8.91 (1H, d, *J* = 1.5 Hz, Ar), 10.89 (1H, s, NH); δ_C_ (DMSO-d_6_) 103.3, 115.8, 116.2 (d, ^2^*J*_CF_ = 21.8 Hz), 119.5, 121.1, 122.9, 125.4, 126.9, 128.4, 128.8, 129.4, 129.8, 130.2, 131.0 (d, ^4^*J*_CF_ = 2.8 Hz), 131.2 (d, ^3^*J*_CF_ = 9.5 Hz), 133.0, 135.7, 137.9, 147.9, 150.7, 152.5, 157.4, 158.3, 162.7 (d, ^1^*J*_CF_ = 272.1 Hz); HRMS (ES): found 589.9715. C_28_H_16_N_3_OFBr_2_^+^ requires 588.9624. *Anal* calcd for C_28_H_15_N_3_OFBr: C, 57.07; H, 2.74; N, 7.13. Found: C, 57.11; H, 2.97; N, 7.12.

#### 2.6.2. 6-Bromo-*N*-(5-bromo-2–(3-fluorophenyl)benzofuran-7-yl)-2–(4-fluorophenyl)quinazolin-4-amine (10b)

Solid (0.32 g, 82%) mp. > 300 °C; ν_max_ (ATR) 683, 775, 874, 937, 1089, 1086, 1239, 1365, 1454, 1594, 1632, 3070, 3171 cm^−1^; δ_H_ (DMSO-d_6_) 7.16 (2H, t, *J* = 9.0 Hz, Ar), 7.39 (2H, d, *J* = 8.5 Hz, Ar), 7.60 (2H, d, *J* = 8.5 Hz, Ar), 7.61 (1H, s, =C-H), 7.81 (1H, dd, *J* = 1.5 and 9.0 Hz, Ar), 7.99 (1H, d, *J* = 8.5 Hz, Ar), 8.12 (1H, d, *J* = 8.5 Hz, Ar), 8.16 (2H, t, *J* = 8.5 Hz, Ar), 8.22 (1H, t, *J* = 8.5 Hz, Ar), 9.03 (1H, d, *J* = 1.5 Hz, Ar), 11.02 (1H, s, NH); δ_C_ (DMSO-d_6_) 103.5, 112.0 (d, ^2^*J*_CF_ = 23.8 Hz), 115.3 (d, ^3^*J*_CF_ = 8.5 Hz), 115.6, 116.1, (d, ^2^*J*_CF_ = 21.9 Hz), 116.5, (d, ^2^*J*_CF_ = 20.9 Hz), 119.8, 121.7, 123.0, 126.8, 128.4, 130.0, 130.2, 131.5 (d, ^3^*J*_CF_ = 9.5 Hz), 130.6 (d, ^2^*J*_CF_ = 8.5 Hz), 131.7, 132.3, 136.5, 137.9, 147.2, 148.0, 152.5, 155.5, 158.0 (d, ^4^*J*_CF_ = 2.9 Hz), 162.7 (d, ^1^*J*_CF_ = 224.7 Hz) 164.7 (d, ^1^*J*_CF_ = 231.3 Hz); HRMS (ES): found 607.9629. C_28_H_16_N_3_OF_2_^79^Br_2_^+^ requires 607.9628. *Anal* calcd for C_28_H_15_N_3_OF_2_Br: C, 55.38; H, 2.49; N, 6.97. Found: C, 55.62; H, 2.57; N, 6.85.

#### 2.6.3. 6-Bromo-*N*-(5-bromo-2–(4-fluorophenyl)benzofuran-7-yl)-2–(4-fluorophenyl)quinazolin-4-amine (10c)

Solid (0.19 g, 87%) mp. 287–289 °C; ν_max_ (ATR) 737, 846, 943, 1088, 1156, 1289, 1365, 1454, 1572, 1607, 1627, 3064, 3170 cm^−1^; δ_H_ (DMSO-d_6_) 7.15 (2H, t, *J* = 8.5 Hz, Ar), 7.22 (1H, t, *J* = 8.5 Hz, Ar), 7.38 (1H, t, *J* = 8.5 Hz, Ar), 7.48 (1H, s, =C–H) 7.66 (1H, d, *J* = 8.5 Hz, Ar), 7.77–7.82 (2H, m, Ar), 7.94–7.97 (2H, m, Ar), 8.08 (1H, d, *J* = 7.5 Hz, Ar), 8.16 (1H, t, *J* = 8.5 Hz, Ar), 8.20–8.23 (1H, m, Ar), 8.99 (1H, d, *J* = 1.5 Hz, Ar), 10.89 (1H, s, NH); δ_C_ (DMSO-d_6_) 102.1, 115.8, 116.2 (d, ^2^*J*_CF_ = 21.8 Hz), 116.5 (d, ^2^*J*_CF_ = 21.8 Hz), 119.5, 121.3, 122.9, 123.5, 124.3, 126.0 (d, ^4^*J*_CF_ = 3.9 Hz), 126.6, 127.7 (d, ^3^*J*_CF_ = 8.5 Hz), 128.5, 129.4 (d, ^4^*J*_CF_ = 2.8 Hz), 130.2, 130.8 (d, ^3^*J*_CF_ = 8.5 Hz), 130.9, 132.6, 137.5, 147.9, 157.8, 161.6, 164.5 (d, ^1^*J*_CF_ = 248.8 Hz), 164.5 (d, ^1^*J*_CF_ = 267.3 Hz); HRMS (ES): found 605.9629. C_28_H_16_N_3_OF_2_Br_2_^+^ requires 605.9628. *Anal* calcd for C_28_H_15_N_3_OF_2_Br: C, 55.38; H, 2.49; N, 6.97. Found: C, 55.59; H, 2.61; N, 7.01.

#### 2.6.4. 6-Bromo-*N*-(5-bromo-2–(3-chlorophenyl)benzofuran-7-yl)-2–(4-fluorophenyl)quinazolin-4-amine (10d)

Solid (0.14 g, 78%) mp. > 300 °C; ν_max_ (ATR) 738, 856, 938, 1030, 1129, 1254, 1347, 1458, 1596, 1634, 3109, 3169 cm^−1^; δ_H_ (DMSO-d_6_) 7.09 (2H, t, *J* = 8.0 Hz, Ar), 7.38 (2H, d, *J* = 8.5 Hz, Ar), 7.58 (1H, s, =C-H), 7.85 (1H, d, *J* = 8.5 Hz, Ar), 7.96 (1H, dd, *J* = 2.5 and 8.5 Hz, Ar), 8.05 (1H, dd, *J* = 1.5 and 8.5 Hz, Ar), 8.07 (2H, d, *J* = 8.5 Hz, Ar), 8.15 (2H, t, *J* = 8.5 Hz, Ar), 8.20 (1H, d, *J* = 1.5 Hz, Ar), 8.90 (1H, d, *J* = 1.0 Hz, Ar), 10.45 (1H, s, NH); δ_C_ (DMSO-d_6_) 103.6, 115.3, 115.5, 116.1 (d, ^2^*J*_CF_ = 21.9 Hz), 119.0, 121.9, 123.6, 124.9, 126.3, 127.3, 128.4, 128.7, 129.2, 129.4, 130.3 (d, ^3^*J*_CF_ = 8.7 Hz), 132.3, 134.6, 137.0, 137.8, 147.3, 149.0 (d, ^4^*J*_CF_ = 3.8 Hz) 149.9, 155.4, 157.5, 158.8, 164.1 (d, ^1^*J*_CF_ = 246.5 Hz); HRMS (ES): found 621.9328. C_28_H_16_N_3_OF^35^Cl^79^Br_2_^+^ requires 621.9327. *Anal* calcd for C_28_H_15_N_3_OFClBr: C, 53.92; H, 2.42; N, 6.74. Found: C, 53.97; H, 2.35; N, 6.848.

#### 2.6.5. 6-Bromo-*N*-(5-bromo-2–(4-(trifluoromethoxy)phenyl)benzofuran-7-yl)-2–(4-fluorophenyl) quinazolin-4-amine (10e)

Solid (0.36 g, 82%) mp. > 300 °C; ν_max_ (ATR) 736, 856, 938, 1060, 1128, 1259, 1347, 1455, 1599, 1634, 3111, 3169 cm^−1^, δ_H_ (DMSO-d_6_) 7.09 (2H, t, *J* = 8.0 Hz, Ar), 7.38 (2H, d, *J* = 8.5 Hz, Ar), 7.56 (1H, s, =C–H), 7.82–785 (4H, m, Ar), 8.04 (1H, d, *J* = 8.5 Hz, Ar), 8.15 (2H, t, *J* = 8.5 Hz, Ar), 8.20 (1H, d, *J* = 1.5 Hz, Ar), 8.90 (1H, d, *J* = 1.0 Hz, Ar), 10.45 (1H, s, NH); δ_C_ (DMSO-d_6_) 103.6, 115.3, 115.5, 116.1 (d, ^2^*J*_CF_ = 21.9 Hz), 119.0, 121.9, 123.6, 124.9, 126.3, 127.3, 128.4, 128.7, 130.3 (d, ^3^*J*_CF_ = 8.7 Hz), 130.9 (t, *J*_CF_ = 25.6 Hz), 132.3, 134.6, 137.0, 137.8, 147.3, 149.0 (d, ^4^*J*_CF_ = 3.8 Hz), 149.9, 155.4, 157.5, 158.8, 164.1 (d, ^1^*J*_CF_ = 246.5 Hz); HRMS (ES): found 672.9227. C_29_H_16_N_3_O_2_F_4_^79^Br_2_^+^ requires 672.9447. *Anal* calcd for C_29_H_15_N_3_OF_4_Br: C, 51.74; H, 2.25; N, 6.24. Found: C, 51.68; H, 2.11; N, 6.35.

#### 2.6.6. 6-Bromo-*N*-(5-bromo-2-phenylbenzofuran-7-yl)-2–(4-chlorophenyl)quinazolin-4-amine (10f)

Solid (0.32 g, 83%) mp. > 300 °C; ν_max_ (ATR) 683, 775, 874, 937, 1089, 1086, 1239, 1365, 1454, 1594, 1632, 3070, 3171 cm^−1^; δ_H_ (DMSO-d_6_) 7.31–7.37 (3H, m, Ar), 7.61 (1H, d, *J* = 8.5 Hz, Ar), 7.65 (1H, d, *J* = 7.0 Hz, Ar), 7.68 (1H, s, =C–H), 7.75 (1H, d, *J* = 7.5 Hz, Ar), 7.89 (1H, d, *J* = 8.7 Hz, Ar), 7.96 (1H, dd, *J* = 2.5 and 8.5 Hz, Ar), 8.07 (1H, d, *J* = 8.7 Hz, Ar), 8.17 (1H, d, *J* = 8.5 Hz, Ar), 8.21 (1H, d, *J* = 2.5 Hz, Ar), 8.26 (1H, d, *J* = 8.5 Hz, Ar), 8.42 (1H, d, *J* = 1.5 Hz, Ar), 8.97 (1H, d, *J* = 8.5 Hz, Ar), 10.71 (1H, s, NH); δ_C_ (DMSO-d_6_), 103.3, 115.6, 119.6, 120.9, 126.4, 127.0, 128.5, 128.7, 129.1, 129.4, 129.9, 130.2, 130.8, 131.7, 132.9, 135.8, 136.9, 137.2, 137.9, 147.9, 150.7, 152.5, 157.2, 157.8, 158.8 161.6; HRMS (ES): found 605.9349. C_28_H_17_N_3_O^35^Cl^79^Br_2_^+^ requires 605.9328. *Anal* calcd for C_28_H_16_N_3_OClBr: C, 55.52; H, 2.66; N, 6.94. Found: C, 55.65; H, 2.78; N, 7.04.

#### 2.6.7. 6-Bromo-*N*-(5-bromo-2–(3-fluorophenyl)benzofuran-7-yl)-2–(4-chlorophenyl)quinazolin-4-amine (10g)

Solid (0.26 g, 81%) mp. > 300 °C; ν_max_ (ATR) 687, 777, 850, 1108, 1217, 1366, 1464, 1519, 1520, 1627, 3102, 3170 cm^−1^; δ_H_ (DMSO-d_6_) 7.15 (1H, t, *J* = 8.0 Hz, Ar), 7.37 (2H, d, *J* = 8.0 Hz, Ar), 7.42 (1H, s, =C–H), 7.66 (1H, d, *J* = 8.5 Hz, Ar), 7.57–7.60 (2H, m, Ar), 7.65 (1H, d, *J* = 8.5 Hz, Ar), 7.83 (1H, d, *J* = 8.5 Hz, Ar), 7.97 (1H, dd, *J* = 1.5 and 9.0 Hz, Ar), 8.10 (2H, d, *J* = 8.5 Hz, Ar), 8.15 (1H, d, *J* = 8.5 Hz, Ar), 9.03 (1H, d, *J* = 1.5 Hz, Ar), 11.00 (1H, s, NH); δ_C_ (DMSO-d_6_) 103.5, 112.0 (d, ^2^*J*_CF_ = 23.8 Hz), 115.4, 116.4 (d, ^2^*J*_CF_ = 20.9 Hz), 119.6, (d, ^4^*J*_CF_ = 2.8 Hz), 121.4, 123.0, 123.8, 124.5, 126.6, 128.5, 129.1, 130.0, 130.2 (d, ^3^*J*_CF_ = 8.5 Hz), 130.5 (d, ^4^*J*_CF_ = 2.9 Hz), 131.7 (d, ^3^*J*_CF_ = 8.6 Hz), 132.3, 136.5, 137.0, 137.9, 147.1, 149.0, 152.5, 155.5, 158.8, 162.7 (d, ^1^*J*_CF_ = 241.7 Hz); HRMS (ES): found 621.9329. C_28_H_16_N_3_OF^35^Cl^79^Br_2_^+^ requires 621.9333. *Anal* calcd for C_28_H_15_N_3_OFClBr: C, 53.92; H, 2.42; N, 6.74. Found: C, 53.99; H, 2.35; N, 6.87.

#### 2.6.8. 6-Bromo-*N*-(5-bromo-2–(4-fluorophenyl)benzofuran-7-yl)-2–(4-chlorophenyl)quinazolin-4-amine (10h)

Solid (0.15 g, 78%) mp. > 300 °C; ν_max_ (ATR) 736, 874, 1061, 1168, 1288, 1398, 1475, 1572, 1629, 3101, 3170 cm^−1^; δ_H_ (DMSO-d_6_) 7.29 (2H, t, *J* = 8.5 Hz, Ar), 7.34 (2H, d, *J* = 8.5 Hz, Ar), 7.47 (1H, s, =C–H), 7.78–7.79 (4H, m, Ar), 7.85 (1H, d, *J* = 8.5 Hz, Ar), 8.04 (1H, d, *J* = 8.0 Hz, Ar), 8.11 (2H, d, *J* = 8.5 Hz, Ar), 8.91 (1H, d, *J* = 1.5 Hz, Ar), 10.89 (1H, s, NH); δ_C_ (DMSO-d_6_) 102.0, 115.4, 116.5 (d, ^2^*J*_CF_ = 21.8 Hz), 119.5, 120.8, 122.9, 123.3, 124.8, 126.1, 126.3, 127.6, 127.7 (d, ^3^*J*_CF_ = 8.5 Hz), 128.8, 129.8, 130.8, 132.5, 135.8, 137.0 (d, ^4^*J*_CF_ = 2.9 Hz), 147.1, 149.9, 155.9, 157.5, 158.7, 162.9 (d, ^1^*J*_CF_ = 245.6 Hz); HRMS (ES): found 621.9333. C_28_H_15_N_3_OF^35^Cl^79^Br_2_^+^ requires 621.9333. *Anal* calcd for C_28_H_14_N_3_OFClBr: C, 53.92; H, 2.42; N, 6.74. Found: C, 53.88; H, 2.58; N, 6.91.

#### 2.6.9. 6-Bromo-*N*-(5-bromo-2–(3-chlorophenyl)benzofuran-7-yl)-2–(4-chlorophenyl)quinazolin-4-amine (10i)

Solid (0.29 g, 67%) mp. 282–285 °C; ν_max_ (ATR) 683, 736, 829, 940, 1012, 1159, 1372, 1501, 1530, 1516, 3048, 3170 cm^−1^; δ_H_ (DMSO-d_6_) 7.35 (1H, d, *J* = 8.5 Hz, Ar), 7.38 (1H, d, *J* = 7.0 Hz, Ar), 7.60 (2H, d, *J* = 8.5 Hz, 2H), 7.67 (2H, d, *J* = 8.5 Hz, Ar), 7.81 (1H, s, C-H), 7.85 (1H, d, *J* = 8.5 Hz), 7.96 (1H, dd, *J* = 2.5 and 8.5 Hz, Ar), 8.05 (1H, dd, *J* = 1.5 and 8.5 Hz, Ar), 8.13 (1H, d, *J* = 8.0 Hz, Ar), 8.17 (1H, d, *J* = 8.5 Hz, Ar), 8.20 (1H, d, *J* = 2.5 Hz, Ar), 8.93 (1H, d, *J* = 1.5 Hz, Ar), 10.51 (1H, s, NH); δ_C_ (DMSO-d_6_) 103.6, 115.6, 119.5, 121.0, 123.0, 124.8, 126.4, 128.4, 129.2, 129.4, 129.8, 130.2, 130.5, 131.3, 131.4, 131.7, 132.2, 134.2, 135.9, 136.9, 137.9, 147.0, 152.4, 155.2, 157.5, 158.6; HRMS: found 637.9041. C_28_H_16_N_3_O^35^Cl_2_^79^Br_2_^+^ requires 637.9037. *Anal* calcd for C_28_H_15_N_3_OCl_2_Br: C, 52.53; H, 2.36; N, 6.56. Found: C, 52.68; H, 2.50; N, 6.45.

#### 2.6.10. 6-Bromo-*N*-(5-bromo-2–(4-(trifluoromethoxy)phenyl)benzofuran-7-yl)-2–(4-chlorophenyl) quinazoline-4-amine (10j)

Solid (0.17 g, 82%) mp. > 300 °C; ν_max_ (ATR) 682, 781, 858, 938, 1091, 1199, 1259, 1372, 1455, 1553, 1615, 3082, 3170 cm^−1^; δ_H_ (DMSO-d_6_) 7.33 (1H, d, *J* = 8.0 Hz, Ar), 7.36 (1H, d, *J* = 8.0 Hz, Ar), 7.59 (1H, s, =C-H), 7.61 (1H, d, *J* = 8.5 Hz, Ar), 7.67 (1H, d, *J* = 8.0 Hz, Ar), 7.81 (1H, d, *J* = 9.0 Hz, Ar), 7.85 (2H, d, *J* = 8.5 Hz, Ar), 7.96 (1H, d, *J* = 9.0 Hz, Ar), 8.04 (1H, d, *J* = 9.0 Hz, Ar), 8.11 (1H, d, *J* = 8.5 Hz, Ar), 8.17 (1H, d, *J* = 8.5 Hz, Ar), 8.20 (1H, d, *J* = 1.5 Hz, Ar), 8.91 (1H, d, *J* = 1.5 Hz, Ar), 10.48 (1H, s, NH); δ_C_ (DMSO-d_6_) 103.6, 115.3, 119.6, 121.9, 123.0, 124.8, 126.3, 127.2, 128.4, 128.7, 129.2, 129.8, 130.7, 131.7, 132.3, 135.8, 136.9 (t, *J*_CF_ = 25.6 Hz), 137.9, 147.2, 149.0, 149.8, 152.4, 155.4, 157.5, 158.6; HRMS (ES): found 687.9236. C_29_H_16_N_3_O_2_F_3_^35^Cl^79^Br_2_^+^ requires 687.9250. *Anal* calcd for C_28_H_15_N_3_O_2_F_3_ClBr: C, 50.50; H, 2.19; N, 6.09. Found: C, 50.29; H, 2.32; N, 6.02.

### 2.7. Materials and methods for the *in vitro* cytotoxicity assay

The cytotoxic activities of the compounds were screened against the C3A/HepG2 human liver cells, human colorectal tumor (Caco-2) cells, the human lung carcinoma (A549) and the cervical cancer (HeLa) cells. The lethal concentration was determined using the 3–(4,5-dimethylthiazol)-2,5-diphenyl tetrazolium bromide (MTT) assay previously developed by Mosmann^20^. The cells were maintained in Dubelsco minimal essential medium (DMEM, Highveld Biological, South Africa) supplemented with 10% fetal calf serum (Adcock-Ingram, Midrand, South Africa) and sodium pyruvate for C3A liver cells. Cell suspensions were prepared from confluent monolayer cultures and plated at a density of 0.1 × 10^6^ cells into each well of 96-well microtitre plates. For cell attachment, plates were incubated for 24 h at 37 °C in a 5% CO_2_ incubator prior to the exposure. The compounds were dissolved in DMSO (5 mg/mL) and appropriate dilutions were prepared, added to the wells and incubated for 48 h. Doxorubicin hydrochloride (Pfizer Laboratories, Sandton, South Africa) was used as a positive control while DMSO was the negative control. After incubation for 48 h, the wells were rinsed with 150 µL of phosphate buffered saline PBS (Sigma-Aldrich, GmBH, Schnelldorf, Germany) and 200 µL of fresh medium was dispensed into the wells. MTT was dissolved in PBS (30 µL) then added to each well and the contents were incubated for 4 h at 37 °C. The medium was removed and MTT formazan crystals were dissolved in 50 µL DMSO. The amount of MTT reduction was measured immediately by detecting the absorbance using a BioTek microplate reader (BioTek Synergy, Analytical and Diagnostic Products, Johannesburg, South Africa) at a wavelength of 570 nm. Each dilution of the test sample was assayed in quadruplicate and the experiments were repeated three times. The percentage of cell viability and the LC_50_ values for each compound tested were calculated as described before^10^.

### 2.8. Apoptosis assay

#### 2.8.1. Annexin V-FITC staining assay on 10d and 10j against C3A and caco-2, respectively

Apoptotic cells were quantified using flow cytometry. The C3A or Caco-2 cells were cultured in 24 well plates and each was then treated with compound **10d** or **10j** at concentrations 5, 12.5 and 25 µM against doxorubicin hydrochloride (0.20 µM) as a reference standard, respectively. After the cells were incubated for 24 h, both treated and untreated cells were harvested, washed two times with ice PBS and then adjusted at a density of 1 x 10^6^ cells/sample. Cells were harvested and transferred into plastic flow tubes (BD Biosciences, South Africa). The cells were, in turn, stained with Annexin-V-FUOS staining kit (Roche, Mannheim, Germany) according to the manufacturer’s instructions. The cells were analyzed using Becton, Dickinson and Company FACS Accuri flow cytometer.

#### 2.8.2. Caspase-3 analysis on 10d and 10j against C3A and caco-2, respectively

Caspase-3 activity was detected by means of Caspase-3 Colorimetric Assay Kit (Abcam, Cambridge, MA, USA). The cells were cultured in 24 well plates and treated for 24 h. The cells were then washed with PBS buffer and lysed on ice. The experiments were carried out according to the manufacturer’s instructions. Optical density was measured at absorbance of 450 nm using the BioTek microplate reader. The concentration of active Caspase-3 (Asp 175) were measured in duplicates and interpolated from the active caspase-3 (Asp 175) standard curve and corrected for sample dilution.

### 2.9. Inhibition for EGFR-TK

The inhibitory activities of compound **10d** or **10j** and Gefitinib towards EGFR-TK were tested using enzyme-linked immunosorbent assay (ELISA) technique with purified epidermal growth factor receptor (Sigma-Aldrich, Bradford, UK). The procedure was carried out according to the manufacturer’s protocol as described in our previous investigation^10^.

### 2.10. Molecular docking studies

The structure of epidermal growth factor receptor (EGFR) kinase domain was obtained from RCSB PDB (id: 1M17)^21^ where all heteroatoms and water molecules were first removed. Polar hydrogen atoms, Kollman-Amber united atom partial charges and solvation parameters were then added by utilizing AutoDockTools^22^.

The coordinates for erlotinib (control) docking were retrieved from the ligand co-crystallised with EGRF (PDB id: 1M17) while the coordinates for compounds **10a**–**j** were generated using ChemDraw Professional 15.0 (PerkinElmer Informatics, Waltham, MA, USA). Minimization of compounds **10** was then performed with Chem3D module in ChemOffice Professional 15.0 (PerkinElmer Informatics). Ligands were in united atom format where only polar hydrogen atoms remained. Gasteiger charges and torsional angles of all ligands were assigned by AutoDockTools.

Hydrated docking was performed with standard protocol from AutoDock4.2 where random water atoms were added around the ligand. A total of 200 runs were performed by AutoDock4.2.6^22^ with semi-empirical free energy scoring function, Lamarckian genetic algorithm of 1,000,000 energy evaluations each run and the maximum number of 27,000 generations. The number of individuals in population was set as 350 and the rate of crossover was 0.8. All docked conformations were clustered with root mean square of 2.0 Å.

## 3. Results and discussion

### 3.1. Chemistry

**Scheme 2.**
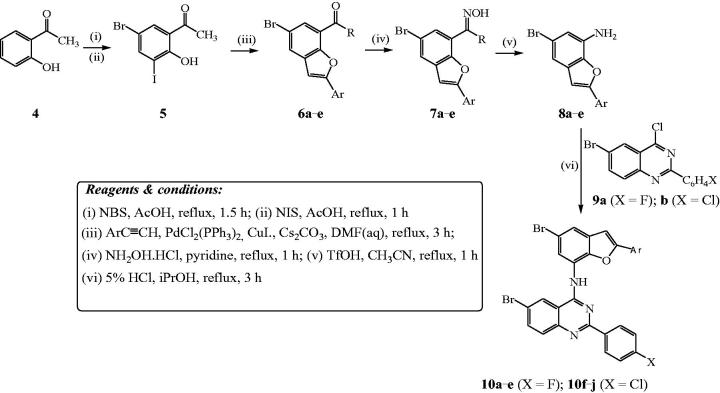
Reaction sequence for the synthesis of **10a**–**j**.

The benzofuran-aminoquinazolines **10a**–**j** and the corresponding intermediates were prepared following the reaction sequence outlined in Scheme 2 and their yields are listed in Table 1. The initial task of this investigation involved the preparation of 5-bromo-2-hydroxy-3-iodoacetophenone **5** to serve as a substrate for the tandem palladium catalyzed Sonogashira cross-coupling with arylacetylenes and subsequent *endo-dig* Csp–O cyclization to afford the requisite 7-acetyl–substituted 2-aryl-5-bromobenzofurans. Recourse to the literature revealed that 5-bromo-2-hydroxy-3-iodoacetophenone has been prepared before by treatment of the commercially available 5-bromo-2-hydroxyacetophenone with pyridinium iodochloride (1 equiv.) in methanol under reflux for 2 h^19^. We opted for the use of commercially available 2-hydroxyacetophenone **4** (purchased from Sigma-Aldrich) as a substrate for initial halogenation with 1 equivalent of *N-*bromosuccinimide (NBS) in acetic acid under reflux for 1.5 h to afford 5-bromo-2-hydroxyacetophenone in 59% yield (Scheme 2). The latter was, in turn, subjected to iodination with *N-*iodosuccinimide (NIS) in acetic acid under reflux for 1 h to afford 5-bromo-2-hydroxy-3-iodoacetophenone **5**. Sonogashira cross-coupling of **5** with terminal acetylenes afforded the corresponding 1–(5-bromo-2-arylbenzofuran-7-yl)ethanones **6a**–**e** in appreciable yields. Oximation of compounds **6a**–**e** with hydroxylamine hydrochloride in pyridine under reflux for 1 h followed by aqueous work-up and recrystallization afforded the corresponding oximes **7a**–**e**. The Beckmann rearrangement of these oximes with 20% mol equivalent of trifluoromethanesulfonic acid (triflic acid, TfOH) in acetonitrile under reflux for 4 h afforded compounds characterized using a combination of NMR, IR and mass spectrometric techniques as the 7-aminobenzofuran derivatives **8a**–**e**. The latter are the result of the initial Beckmann rearrangement via aryl carbon migration followed by an *in situ* acid-mediated hydrolysis of the intermediate 7-acetamido-2-aryl-5-bromobenzofuran derivatives. Hitherto, Cacchi *et al.*^23^ had reported a method for the synthesis of the 7-aminobenzofuran derivatives which makes use of the Buchwald-Hartwig C–N bond formation of the intermediate 7-bromobenzofurans with the primary and secondary amines. Despite what looks like a simple molecular framework, we found that no attempts have been made before towards the synthesis of benzofuran-quinazoline hybrids in which the two pharmacophores are linked through a heteroatom bridge. Consequently, we reacted the nucleophilic 7-aminobenzofurans **8a**–**e** with the electrophilic 6-bromo-4-chloro-2–(4-halogenophenyl)quinazoline **9a** (X = F) or **9 b** (X = Cl) in the presence of 5% HCl in isopropanol (iPrOH) under reflux for 4 h to afford the corresponding benzofuran-aminoquinazoline hybrids **10a**–**e** or **10f**–**j**, respectively. Dechloroamination was confirmed by the presence of increased number of proton and carbon signals in the aromatic region of the ^1^H- and ^13^C-NMR spectra of compounds **10a–j**.

**Table 1.** Percentage yields of compounds **10a**–**j** and the corresponding intermediates.

Ar	%Yield **6**	%Yield **7**	%Yield **8**	%Yield **10a**–**e**	%Yield **10f**–**j**
C_6_H_5_-	73 (**6a**)	93 (**7a**)	62 (**8a**)	75 (**10a**)	83 (**10f**)
3-FC_6_H_4_-	76 (**6b**)	86 (**7b**)	78 (**8b**)	82 (**10b**)	81 (**10g**)
4-FC_6_H_4_-	76 (**6c**)	72 (**7c**)	71 (**8c**)	87 (**10c**)	78 (**10h**)
3-ClC_6_H_4_-	74 (**6d**)	81 (**7d**)	65 (**8d**)	78 (**10d**)	67 (**10i**)
4-CF_3_OC_6_H_4_-	77 (**6e**)	78 (**7e**)	85 (**8e**)	82 (**10e**)	84 (**10j**)

**Figure 2.**
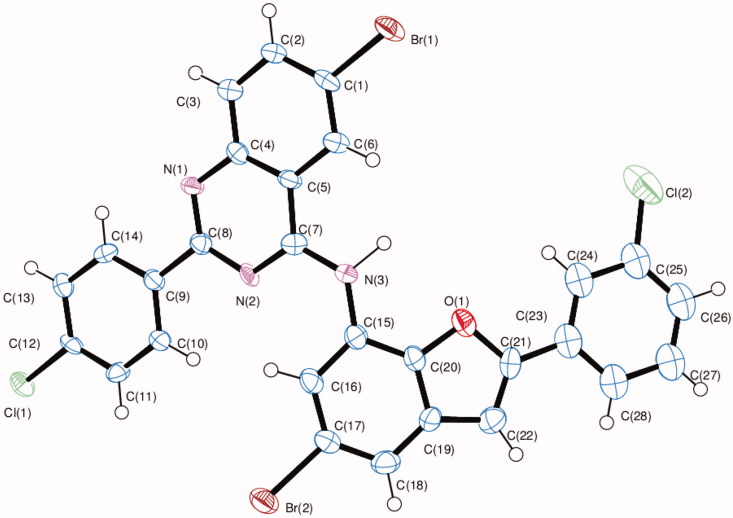
Oak Ridge Thermal Ellipsoid Plot (ORTEP) diagram of 10f. Displacement ellipsoids are drawn at the 50% probability level and H atoms are shown as small spheres of arbitrary radii.

The structure of these compounds was distinctly confirmed by single crystal X-ray diffraction (XRD) analysis of compound **10f** (Figure 2)^24^. The crystal structure reveals the presence of an intramolecular hydrogen bonding between the amine proton and endocyclic oxygen atom.

Since the molecular construct of compounds **10a**–**j** resembles that of the EGFR-TK inhibitor, Gefitinib, we decided to evaluate them for antiproliferative effect against a panel of EGFR-positive cancer cell lines, namely, the A549, Caco-2, C3A (HepG2/C3A) and HeLa cell lines. The structure activity relationship (SAR) of these compounds was studied with respect to the substitution pattern on the phenyl rings of the quinazoline and benzofuran scaffolds.

### 3.2. Biological evaluation

#### 3.2.1. *In vitro* cytotoxicity of quinazoline-benzofuran hybrids 10a–j

Compounds **10a**–**j** were evaluated for potential antiproliferative properties *in vitro* using the well-established 3–(4,5-dimethylthiazole-2-yl)-2,5-diphenyltetrazoliumbromide based colorimetric cell viability (MTT) assay. The compounds were screened as DMSO solutions at concentrations ranging from 5–100 µM and their cytotoxic activities were expressed as LC_50_ values (the dose that reduces survival to 50%) in µM concentrations using DMSO as the negative control and Gefitinib as a reference standard. We used DMSO as a negative control because at 1% or less this substance has been found to exhibit no apparent effect on proliferation of the HeLa and Caco-2 cells up to 48 h^25^^,^^26^. Likewise, Gefitinib was used as a positive control because it has previously been found to enhance apoptosis and suppress proliferation of lung cancer (A549) cells^27^. Moreover, the Caco-2 cells have also been found to display greater sensitivity to this drug due to their high EGFR expression^26^. The corresponding LC_50_ values of the tested compounds against Gefitinib are represented in Table 2. We observed variable inhibition of cell growth of the A549 (modest), Caco-2 (intermediate), C3A (highly sensitive) and HeLa (resistant) cells against Gefitinib, which seem not to correlate the level of EGFR expression. The sensitivity to growth inhibition by Gefitinib has been associated with dependence on protein kinase B/AKT (Akt) and extracellular signal-regulated kinase 1/2 (ERK1/2) activation in response to EGFR signaling for survival and proliferation and extent of EGF-induced down-regulation of cell surface EGFR^28^. The presence in **10a** of a phenyl group on the benzofuran ring and 2–(4-fluorophenyl) substituent on the quinazoline framework led to diminished cytotoxicity against the A549, Caco-2 or C3A cell lines, but significant cytotoxicity against the HeLa cells with LC_50_ value of 17.6 µM. Compound **10b** substituted with a 3-fluorophenyl group on the benzofuran ring and a 4-fluorophenyl group on the quinazoline moiety resulted in moderate cytotoxicity against the A549 cells (LC_50_ = 54.3 µM) and no activity against the other three cells. No cytotoxicity was observed against the Caco-2 and C3A cell lines for compound **10c** substituted with the 4-fluorophenyl group at the 2-positions of the benzofuran and quinazoline moieties. However, this compound was found to exhibit significant cytotoxicity against the A549 (LC_50_ = 52.6 µM) and the HeLa (LC_50_ = 11.4 µM) cell lines when compared to Gefitinib. A combination of 3-chlorophenyl on the benzofuran ring and 4-fluorophenyl group on the quinazoline framework in **10d** was found to result in reduced cytotoxicity against the A549 and Caco-2 cell lines, but increased anti-growth effect against the C3A (LC_50_ = 9.0 µM) and the HeLa (LC_50_ = 22.1 µM) cell lines when compared to Gefitinib. Compound **10e** substituted with a 4-trifluoromethoxy group on the benzofuran ring and a 4-fluorophenyl group on the quinazoline framework exhibits moderate cytotoxicity against the HeLa cells (LC_50_ = 23.4 µM) and no activity against the other three cell lines. Within the series **10f**–**j**, compound **10f** substituted with a phenyl ring on the benzofuran ring and a 4-chlorophenyl group at C-2 position of the quinazoline was found to exhibit significant cytotoxicity against the A549 (LC_50_ = 48.0 µM) and the HeLa (LC_50_ = 15.4 µM) cell lines when compared to Gefitinib. Compound **10g** substituted with a 4-chlorophenyl group at the C-2 position of the quinazoline framework and a 3-fluorophenyl ring on the benzofuran moiety, on the other hand, exhibited no activity against all the four cell lines. Likewise, a combination of 4-chlorophenyl group on the quinazoline ring and a 4-fluorophenyl group on the benzofuran arm resulted in loss of activity for **10h** against the A549, Caco-2 and C3A cell lines. However, this compound exhibited significant cytotoxicity against the HeLa cell lines (LC_50_ = 28.6 µM). Compound **10i** substituted with a 4-chlorophenyl ring on position 2 of the quinazoline and 3-chlorophenyl group on the 2-position of the benzofuran moiety was found to exhibit significant cytotoxicity against the Caco-2 cell line with the LC_50_ value of 33.5 µM, but no activity against the other three cell lines. The presence of a 4-trifluoromethoxyphenyl group on the benzofuran framework in compound **10j** led to significant cytotoxicity against the A549 (LC_50_ = 47.4 µM) and HeLa (LC_50_ = 28.1 µM) and more so against the Caco-2 cell line (LC_50_ = 18.4 µM) with no activity against the C3A cells when compared with Gefitinib. Compounds substituted with the strong electron-withdrawing trifluoromethyl group have been found to exhibit superior metabolic stability and improved activity profile compared to the corresponding methyl analogs^29^. A combination of this moiety with a 4-chlorophenyl group on the quinazoline framework seems to be more desirable for cytotoxicity than with a 2–(4-fluorophenyl) ring, which resulted in diminished cytotoxicity for **10e**.

**Table 2.** Cytotoxic effects of **10a**–**j** and Gefitinib against A549, Caco-2, C3A and HeLa cell lines.

	
	Cancer cells LC_50_ (µM)					
Compound	R	A549	Caco-2	C3A	HeLa	
**10a**	H	64.7 ± 0.11	96.3 ± 0.10	81.0 ± 0.03	17.6 ± 0.39	
**10b**	3-F	54.3 ± 0.19	83.9 ± 0.07	90.1 ± 0.02	47.0 ± 0.38	
**10c**	4-F	52.6 ± 0.20	98.8 ± 0.02	>100	11.4 ± 0.56	
**10d**	3-Cl	65.1 ± 0.12	47.7 ± 0.01	9.0 ± 0.01	22.1 ± 1.10	
**10e**	4-OCF_3_	97.6 ± 0.07	65.2 ± 0.10	>100	23.4 ± 0.49	
**10f**	H	48.0 ± 0.10	75.0 ± 0.02	73.9 ± 0.02	15.4 ± 0.45	
**10g**	3-F	78.5 ± 0.15	94.4 ± 0.02	79.1 ± 0.01	53.5 ± 4.46	
**10h**	4-F	74.1 ± 0.21	49.1 ± 0.01	>100	28.6 ± 1.12	
**10i**	3-Cl	86.6 ± 0.16	33.5 ± 0.10	68.2 ± 0.02	75.1 ± 5.25	
**10j**	4-OCF_3_	47.4 ± 0.07	18.4 ± 0.07	>100	28.1 ± 0.79	
**Gefitinib**		51.3 ± 0.17	27.9 ± 1.05	5.0 ± 0.04	98.8 ± 0.56	

We took into consideration the pro-apoptotic properties of the 4-anilinoquinazolines^6^^,^^30^, and benzofuran derivatives^31^ and decided to verify whether the observed inhibition of cell growth by compounds **10** was related to cell apoptosis. Apoptosis is an essential mechanism by the body to get rid of unwanted cells and induction of this process in cancer cells would lead to automatic death and decrease cancer proliferation.

#### 3.2.2. Evaluation of cell death pathways

**Figure 3.**
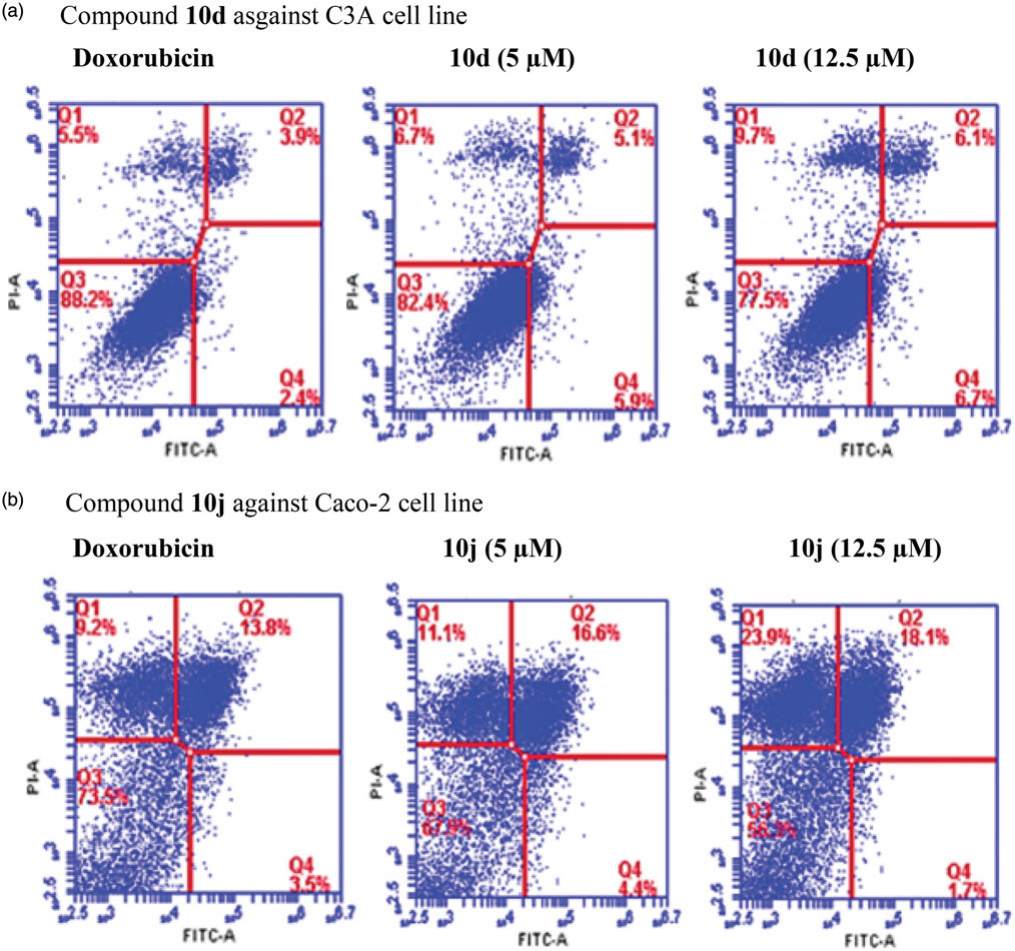
Effects of compounds **10d** and **10j** on the induction of apoptosis in C3A (3a) and Caco-2 cells (3b) as determined by Annexin V/PI staining. Data represent the percentage of apoptotic cells for the control and compounds **10d** and **10j** at 5 and 12.5 µM after 24 h.

We selected compounds **10d** and **10j** to evaluate whether they induce apoptosis in the C3A and Caco-2 cell lines against doxorubicin hydrochloride as a reference standard, respectively. The population of apoptotic cells was determined by means of Annexin-V and propium iodide (PI) double staining by flow cytometry, which is a useful tool for evaluation of molecular and morphological events that take place during cell death and cell proliferation. The Annexin V-FITC/PI apoptosis assay is a popular method to distinguish between viable cells (Annexin V-negative; PI-negative), early apoptotic cells (Annexin V-positive; PI-negative), late apoptotic cells (Annexin V-positive; PI-positive) and unviable or necrotic cells (Annexin V-negative; PI-positive) through differences in plasma membrane integrity and permeability. After treatment with different concentrations of compound **10d** and **10j** (5 and 12.5 µM) for 24 h, the C3A and Caco-2 cell lines were stained with propidium iodide (PI) and Annexin V binding buffer, and then analyzed by the flow cytometry. The percentage of cell populations, i.e., necrotic cell (Q1), late apoptotic cells (Q2), healthy cells (Q3) and early apoptotic cells (Q4) are represented in Figure 3. As shown in Figure (3(a)) and (3(b)), compounds **10d** and **10j** induced apoptosis in the C3A and Caco-2 cell lines, respectively. Both necrotic (Q1) and apoptotic cells (Q2) seem to increase with the increasing concentration, but the population of necrotic cells remained higher at the highest concentration tested. This is presumably because necrosis takes place after the cells have already died by any mechanism including apoptosis and have reached equilibrium with their surroundings^32^^,^^33^.

**Figure 4.**
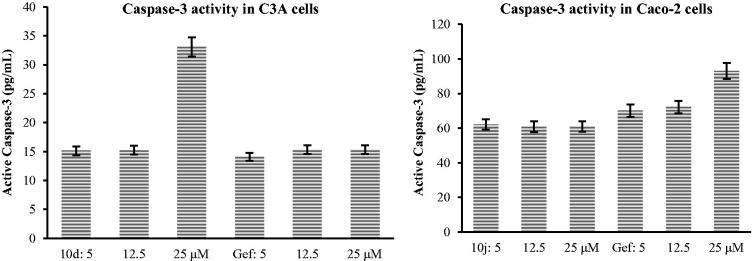
Effects of compounds **10d** and **10j** on Human Caspase-3 (Asp175) activity in C3A and Caco-2 cells against Gefinitib (Gef), respectively.

A major biochemical feature of apoptosis is the activation of caspases (caspase-2, -3, -6, -7, -8, -9, and -10), which play a central role in the morphological changes associated with apoptosis^34^. Activation of caspase-3, for example, is critically important for the induction of apoptotic pathways^34^. Consequently, we evaluated compounds **10d** and **10j** for potential to induce caspase-3 activation in C3A and Caco-2 cells using Gefitinib as a reference standard (Figure 4). The results of this study indicate that these compounds induce apoptosis through activation of caspase-3 that subsequently leads to cell membrane alterations.

The hepatocellular carcinoma and the colorectal adenocarcinoma cell lines have been found to express high levels of EGFR and sensitivity to Gefitinib^27^^,^^35^^,^^36^. Inhibition of EGFR-TK activity is regarded as the most promising approach for innovative therapeutic strategies in cancer treatment^37^.

#### 3.2.3. Inhibition of EGFR-TK by compounds 10a–j

In order to test whether the benzofuran-appended 4-aminoquinazolines prepared in this investigation inhibit the ligand binding-induced receptor phosphorylation, we performed kinase activity of the human EGFR in the presence of compounds **10a**–**j** against Gefitinib as the reference standard (Table 3). Within the series **10a**–**e**, compounds **10a** and **10b** substituted with a 2–(4-fluorophenyl) ring on the quinazoline framework and a phenyl or 3-fluorophenyl ring on the benzofuran arm were found to exhibit reduced inhibitory effect against the EGFR when compared to the reference standard. A combination of 4-fluorophenyl group at the 2-positons of the quinazoline and benzofuran moieties in **10c**, on the other hand, resulted in moderate inhibitory effect with an IC_50_ value of 52.2 nM. The most cytotoxic compound **10d** against the C3A cell line was found to inhibit the EGFR (IC_50_ = 29.3 nM) slightly higher than the reference drug. The presence of a 3–(4-fluoromethoxyphenyl) group on the benzofuran arm of **10e** also resulted in increased inhibitory effect (IC_50_ = 31.1 nM) when compared to Gefitinib. Derivatives **10f**–**j** substituted with a 2–(4-chlorophenyl) group on the quinazoline framework were generally less active against the EGFR. Only compound **10j** found to exhibit significant cytotoxicity against the A549, Caco-2 and Hela cells showed moderate EGFR inhibitory activity (IC_50_ = 61.5 nM) compared to the reference standard. The hepatocellular carcinoma cells have been found to display greater sensitivity to Gefitinib due to high EGFR expression^38^^,^^39^. Based on the observed results, we can conclude that the EGFR-TK inhibitory activity of the benzofuran-aminoquinazolines **10** is correlated to their antiproliferative activities.

**Table 3.** IC_50_ values (in nM) of **10a**–**j** and Gefitinib against EGFR-TK.

Compound	IC_50_ (nM)
**10a**	111.3 ± 0.42
**10b**	122.5 ± 0.50
**10c**	52.2 ± 0.18
**10d**	29.3 ± 0.02
**10e**	31.1 ± 0.12
**10f**	40.4 ± 0.16
**10g**	132.9 ± 0.55
**10h**	125.7 ± 0.52
**10i**	90.2 ± 0.34
**10j**	61.5 ± 0.01
**Gefitinib**	33.1 ± 0.02

EGFR tyrosine kinase is often used as a model in molecular docking (*in silico*) studies to predict the hypothetical protein-ligand binding mode. To help us understand the anticancer activity of compounds **10** and guide further structure activity relationship (SAR) studies, we performed molecular docking of compounds **10** into EGFR-TK active site against Gefitinib.

#### 3.2.4. Molecular docking of compounds 10a–j into the ATP binding site of EGFR

**Figure 5.**
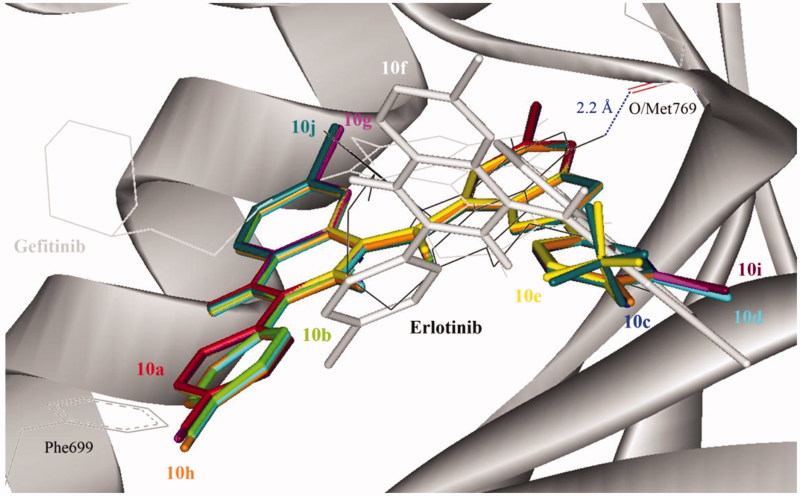
Docked conformation of Erlotinib (as docking control), Gefitinib and 4-aminoquinazoline–appended 2-arylbenzofurans (compound **10**; stick representation) in the binding pocket of epidermal growth factor receptor (EGFR) kinase domain (surface and ribbon representation). Blue dotted line is the hydrogen bonding formed between erlotinib and EGFR. T-shape π-stacking interaction between the chlorophenyl/fluorophenyl of **10a**–**e** and **10g**–**j** with EGFR Phe699 was observed.

In order to explore probable interaction model of inhibitors and enzyme active site, molecular docking of **10d** and **10j** into ATP binding site of EGFR kinase was performed on the binding model based on the EGFR complex structure (Figure 5). A crystal structure of EGFR that previously co-crystalized with Erlotinib (an inhibitor of EGFR) was obtained from the protein data bank (PDB ID: 1M17). The control (Gefitinib) docked on Erlotinib binding site produced root mean square deviation (RMSD) of 1.6 Å with the crystal structure. The morpholine region of Gefitinib was positioned as anti-parallel with Phe699 side chain, but no such orientation was observed in Erlotinib. In addition, the 3-chloro-4-fluorophenyl moiety of Gefitinib also docked deeper into the EGFR binding pocket compared with Erlotinib. The binding conformations of the 2-arylbenzofuran–appended 4-aminoquinazoline hybrids **10a**–**j** are depicted in Figure 5 and these were similar for all of the compounds except for compound **10f**, which docked at the entrance of the binding pocket.

Direct hydrogen bond formation between the ligand and the receptor was only observed with Erlotinib (Hb distance = 2.2 Å) despite the fact that the 2-arylbenzofuran–appended 4-aminoquinazoline hybrids **10** with the exception of **10f** bind on the same site as this reference drug. The aminoquinazoline and chloro/fluorophenyl moieties of compounds **10a**–**e** and **10g**–**j**, on the other hand, extended towards the entrance of the binding pocket. The chloro/fluorophenyl rings of these compounds (with the exception of **10f**) formed T-shape π-stacking interaction with Phe699 that might contribute towards the favorable binding affinity with EGFR. Compared to the docking mode, it could be assumed that the synthesized compounds might act on the ATP binding site of EGFR like Gefitinib does.

## 4. Conclusions

In summary, we have developed a simple method for linking the 2-arylbenzofuran and quinazoline scaffolds through an amino bridge to comprise analogs of the medicinally important 4-anilinoquinazolines or Cediranib (**2**). Some of the prepared benzofuran-aminoquinazoline hybrids exhibited moderate to significant cytotoxicity *in vitro* against the A549 and HeLa cancer cell lines. Although most of these compounds are less active against the Caco-2 and C3A cell lines, a combination of a 2–(3-chlorophenyl) group on the benzofuran moiety and a 2–(4-fluorophenyl) group on the quinazoline framework of **10d** resulted in significant cytotoxicity against the C3A cell line (LC_50_ = 9.0 µM) when compared to Gefitinib (LC_50_ = 5.01 µM). Likewise, compound **10j** substituted with a 4-chlorophenyl group at the 2-position of the quinazoline moiety and 4-trifluoromethoxyphenyl group at the 2-position of the benzofuran framework was found to exhibit increased cytotoxicity against the Caco-2 cells (LC_50_ = 18.4 µM) more so than Gefitinib (LC_50_ = 27.9 µM). Compounds **10a**–**j** were found to exhibit moderate to significant cytotoxicity against the HeLa cells with the exception of **10b, 10g** and **10i** substituted with a fluorine or chlorine atom on the 3-position of the phenyl ring on the benzofuran arm. Compounds **10d** and **10j**, which were selected for further evaluation of the mode of cell death were able to trigger apoptosis in C3A & Caco-2 cells, respectively. This demonstrates that the benzofuran-aminoquinazoline hybrids prepared in this investigation exhibit cytotoxicity and pro-apoptotic properties. Compounds **10d** and **10j** not only demonstrated strong antiproliferation activities against some of the tested cancer cell lines, but also showed significant inhibitory activity towards EGFR (IC_50_ = 29.3 nM and 61.5 nM, respectively) compared to the medicinally important EGFR inhibitor, Gefitinib (IC_50_ = 33.1 nM). Molecular docking studies into the EGFR also demonstrated that the benzofuran-aminoquinazoline hybrids prepared in this investigation have potential to inhibit EGFR-TK phosphorylation. Since the title compounds and their derivatives may also target protein kinases other than EGFR, future studies will be extended to other types of protein kinases to explore further their mechanism of action and selectivity.

## Supplementary Material

GENZ-2018-0165_Supplementary_Data_CorrectionsV1_.docx
