# Supplementary material for: Benzofuran–appended 4-aminoquinazoline hybrids as epidermal growth factor receptor tyrosine kinase inhibitors: synthesis, biological evaluation and molecular docking studies
Source: J Enzyme Inhib Med Chem. 2018 Oct 2;33(1):1516–28. doi: 10.1080/14756366.2018.1510919 (PMC6171423; doi:10.1080/14756366.2018.1510919)
Supplement: GENZ-2018-0165_Supplementary_Data_CorrectionsV1_.docx [file IENZ_A_1510919_SM3011.docx]

**Supplementary Information:**

**Benzofuran–appended 4-Aminoquinazoline Hybrids as Epidermal Growth Factor Receptor Tyrosine Kinase Inhibitors: Synthesis, Biological Evaluation and Molecular Docking Studies**

M.J. Mphahlele, M.M. Maluleka, A. Aro, L.J. McGaw and Y.S. Choong

**Figure S1:** ^1^H- and ^13^C-NMR spectra of compounds **6a**–**e**, **7a**–**e**, **8a**–**e**, **10a**–**j**

**Figure S1: ^1^H- and ^13^C-NMR spectra of compounds 6a–e, 7a–e, 8a–e, 10a–j**


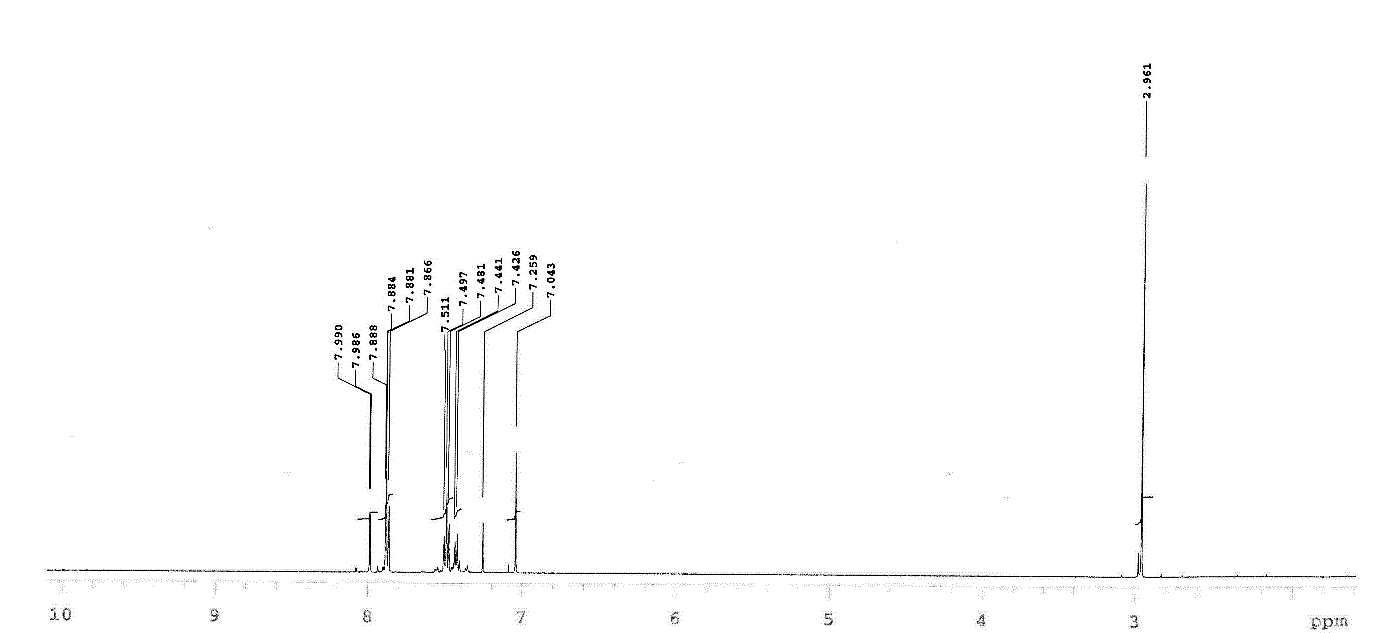

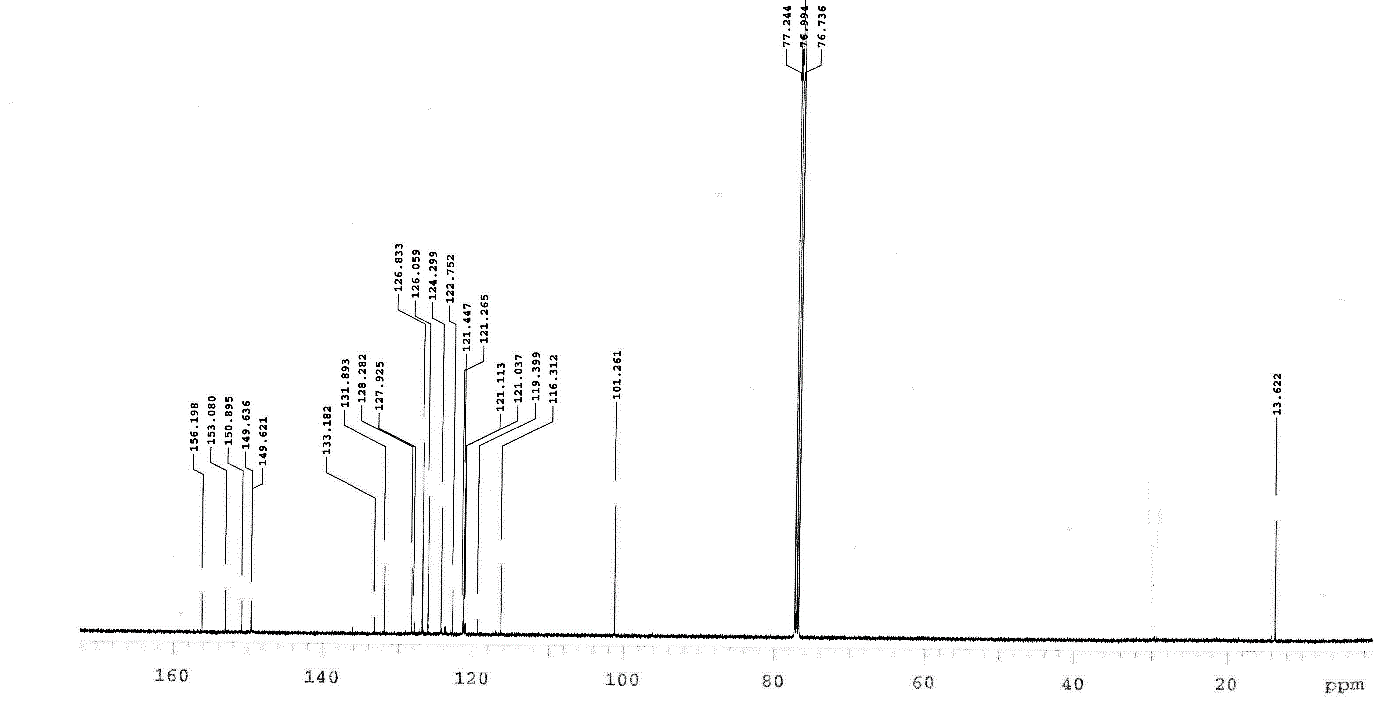


**Figure S1.1:** ^1^H- and ^13^C-NMR spectra of **6a** in CDCl_3_ at 500 MHz and 125 MHz, respectively.


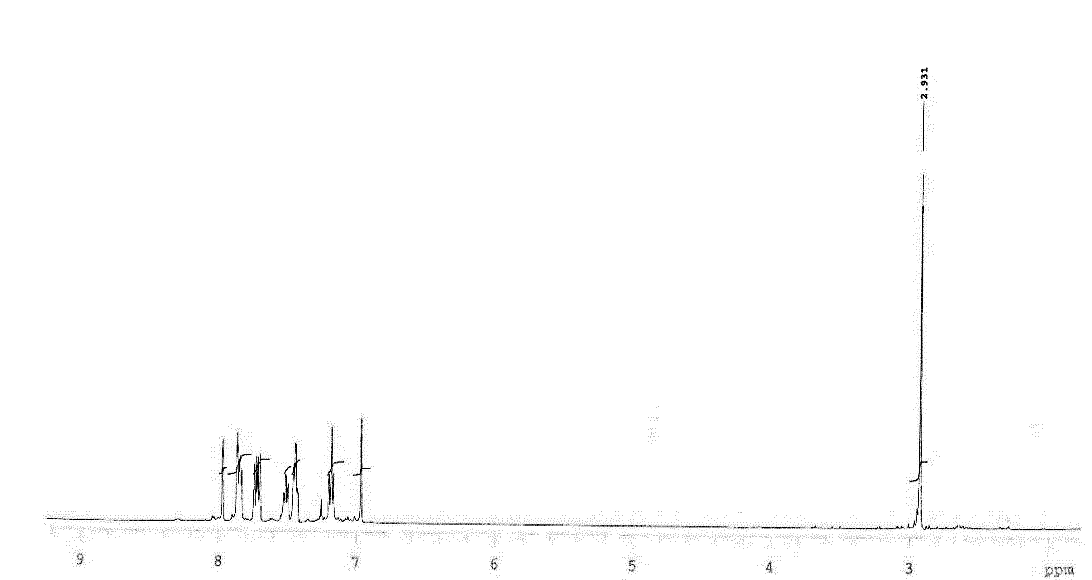

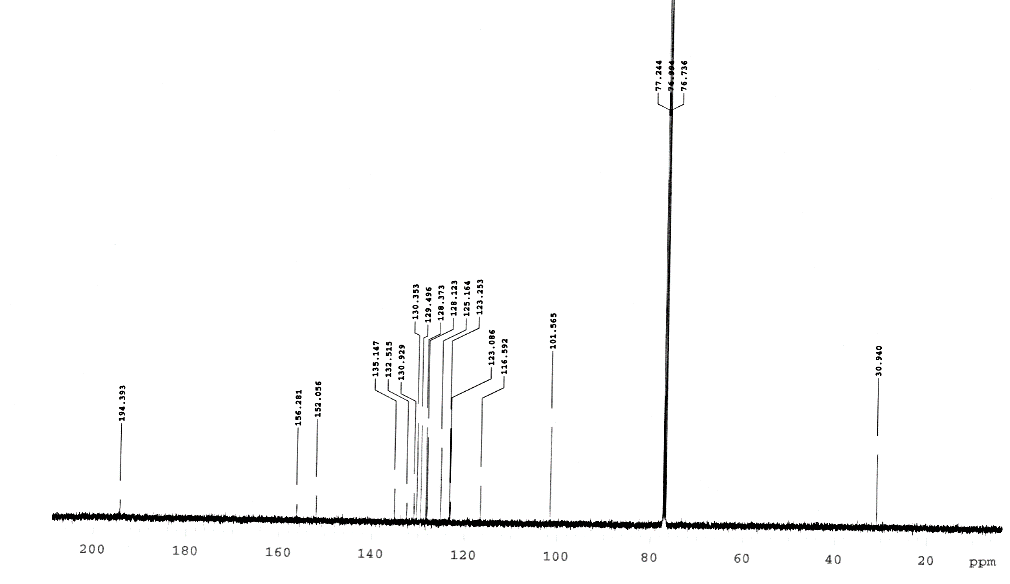


**Figure S1.2:** ^1^H- and ^13^C-NMR spectra of **6b** in CDCl_3_ at 500 MHz and 125 MHz, respectively.


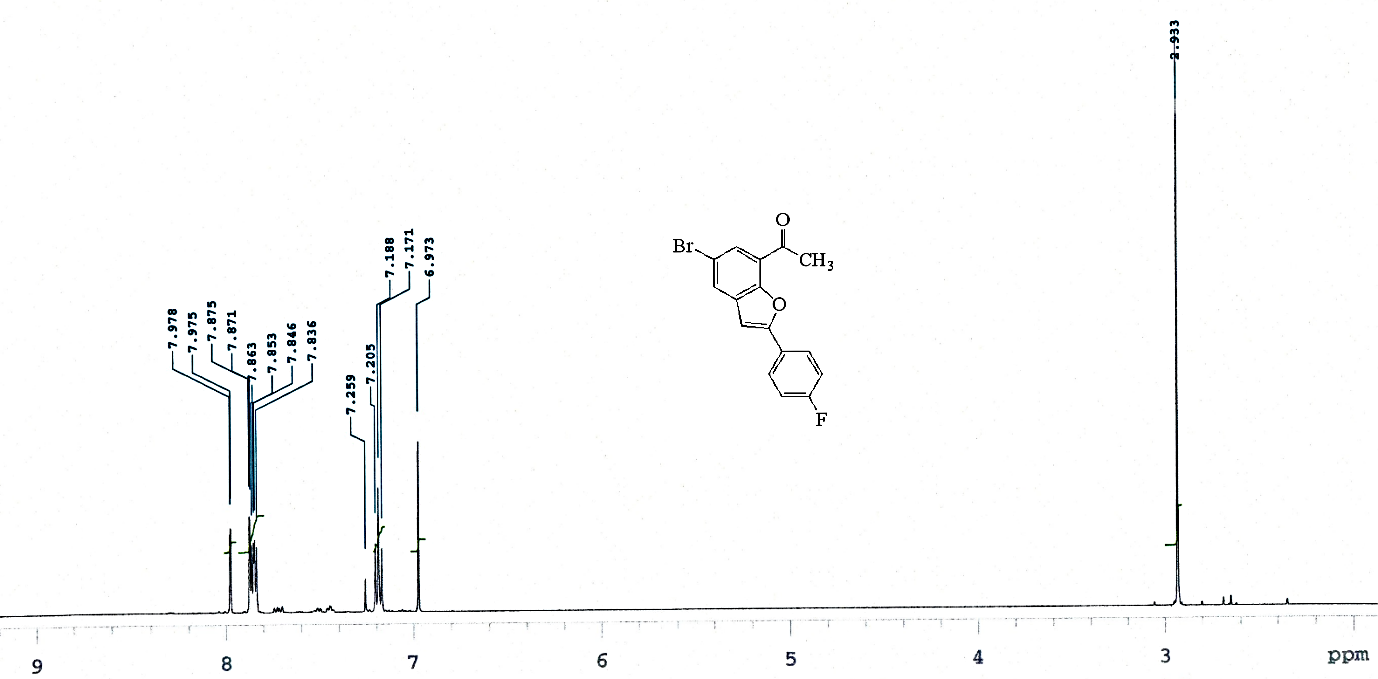


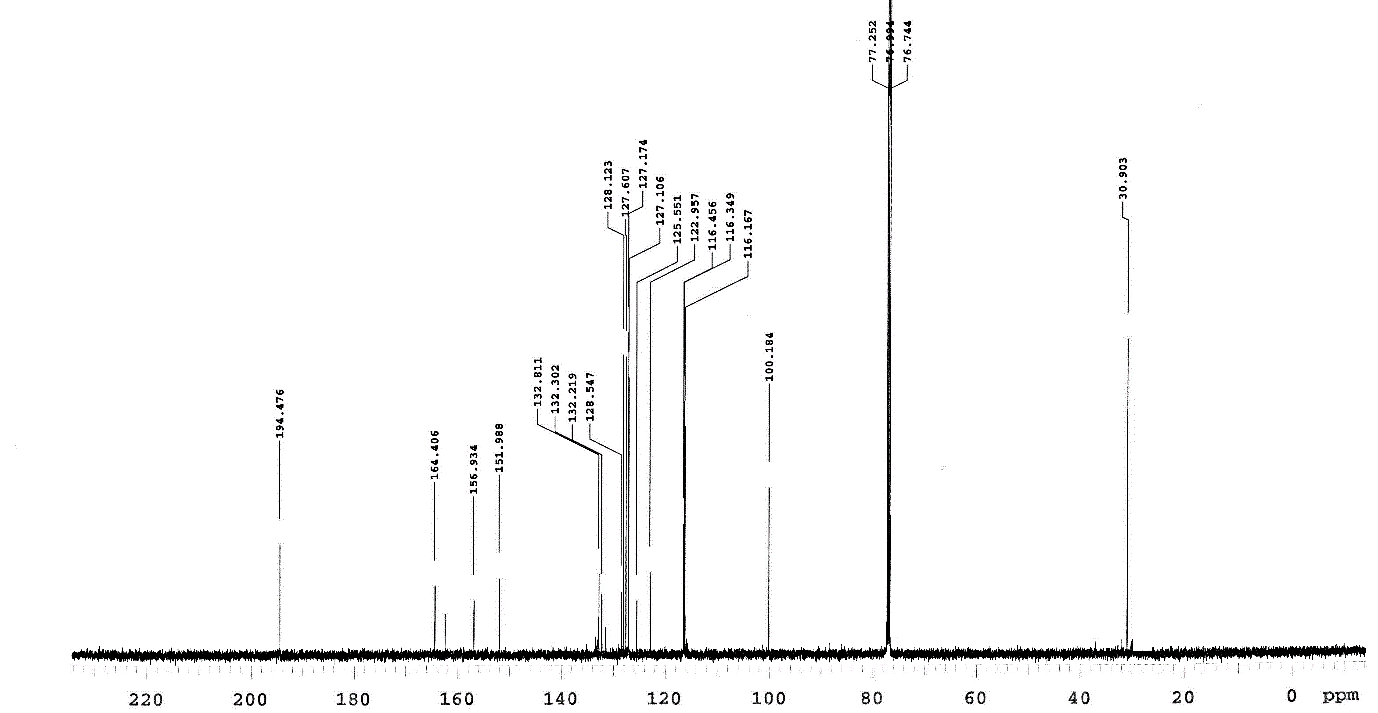


**Figure S1.3:** ^1^H- and ^13^C-NMR spectra of **6c** in CDCl_3_ at 500 MHz and 125 MHz, respectively.


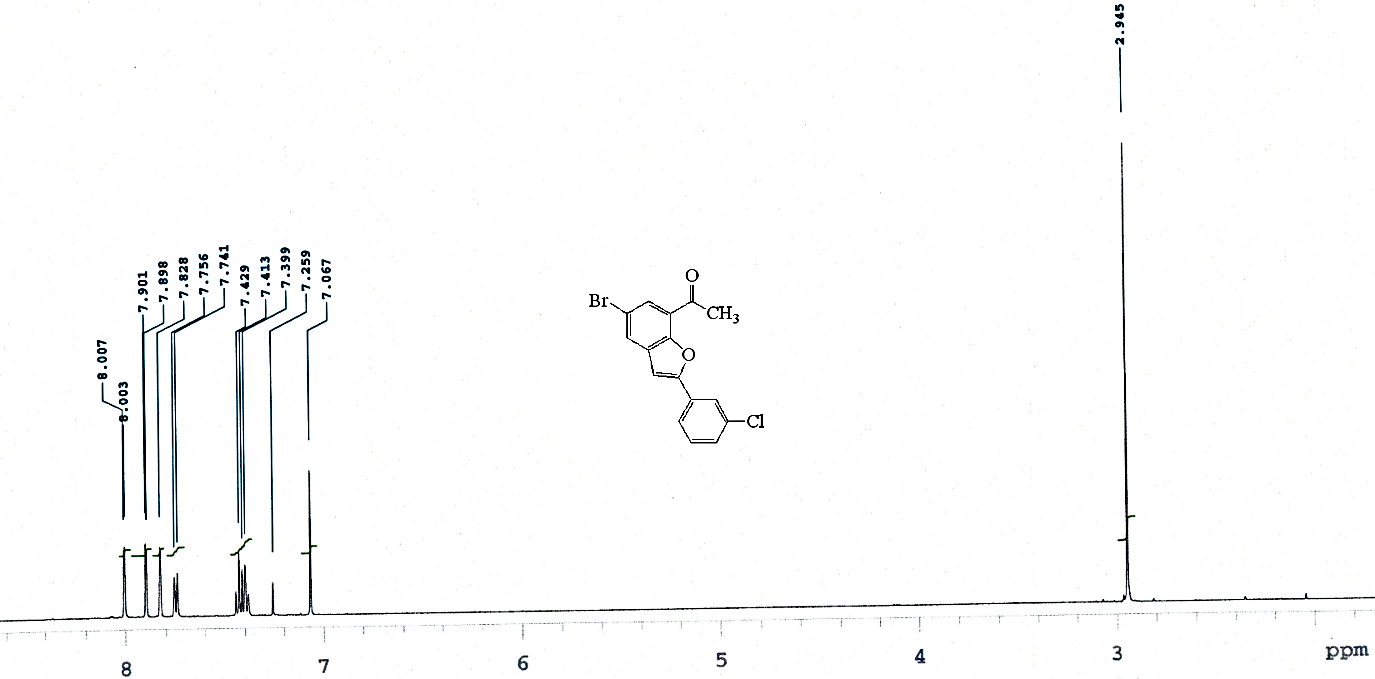

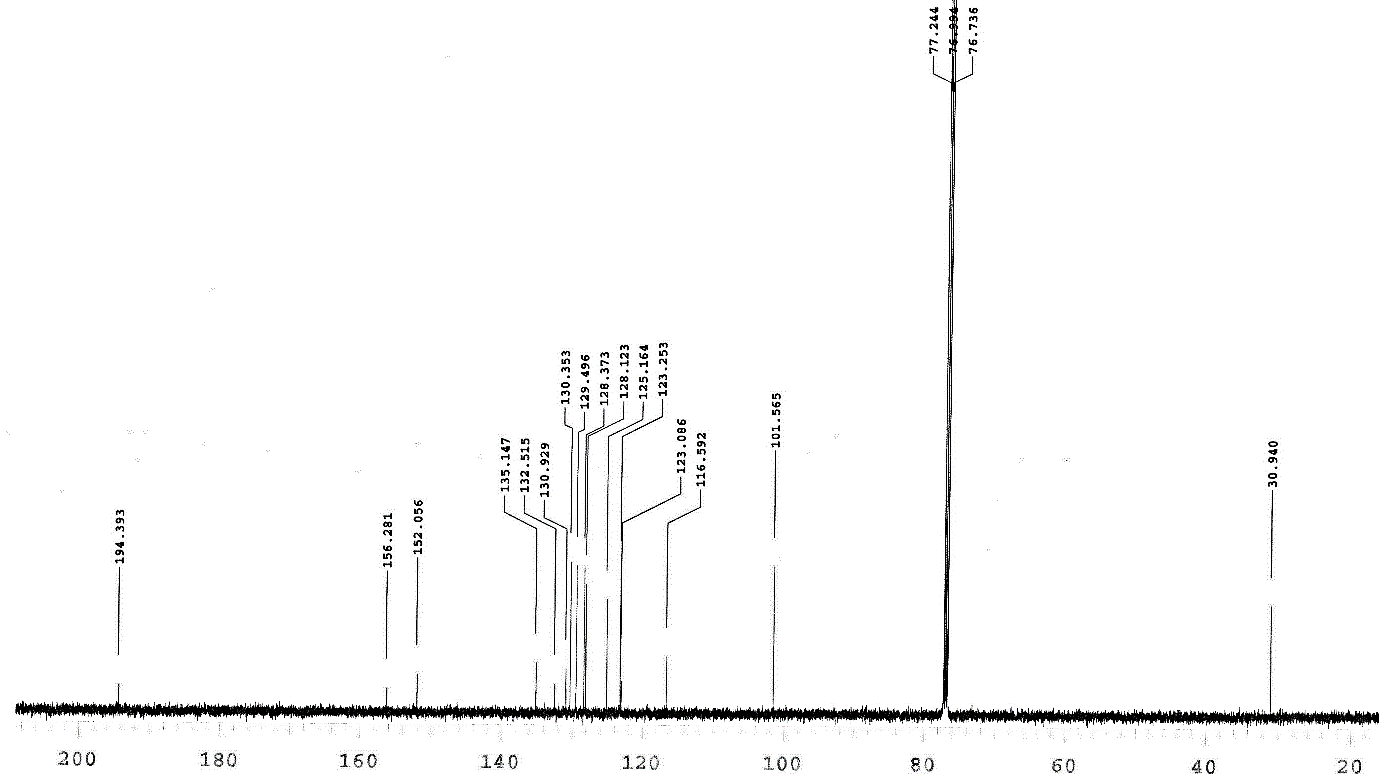


**Figure S1.4:** ^1^H- and ^13^C-NMR spectra of **6d** in CDCl_3_ at 500 MHz and 125 MHz, respectively.


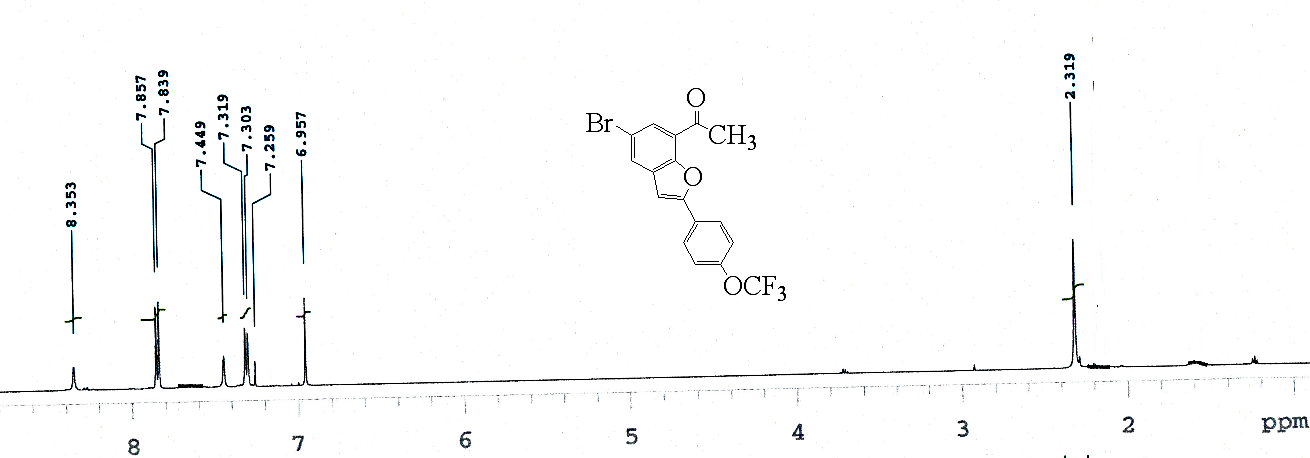


**
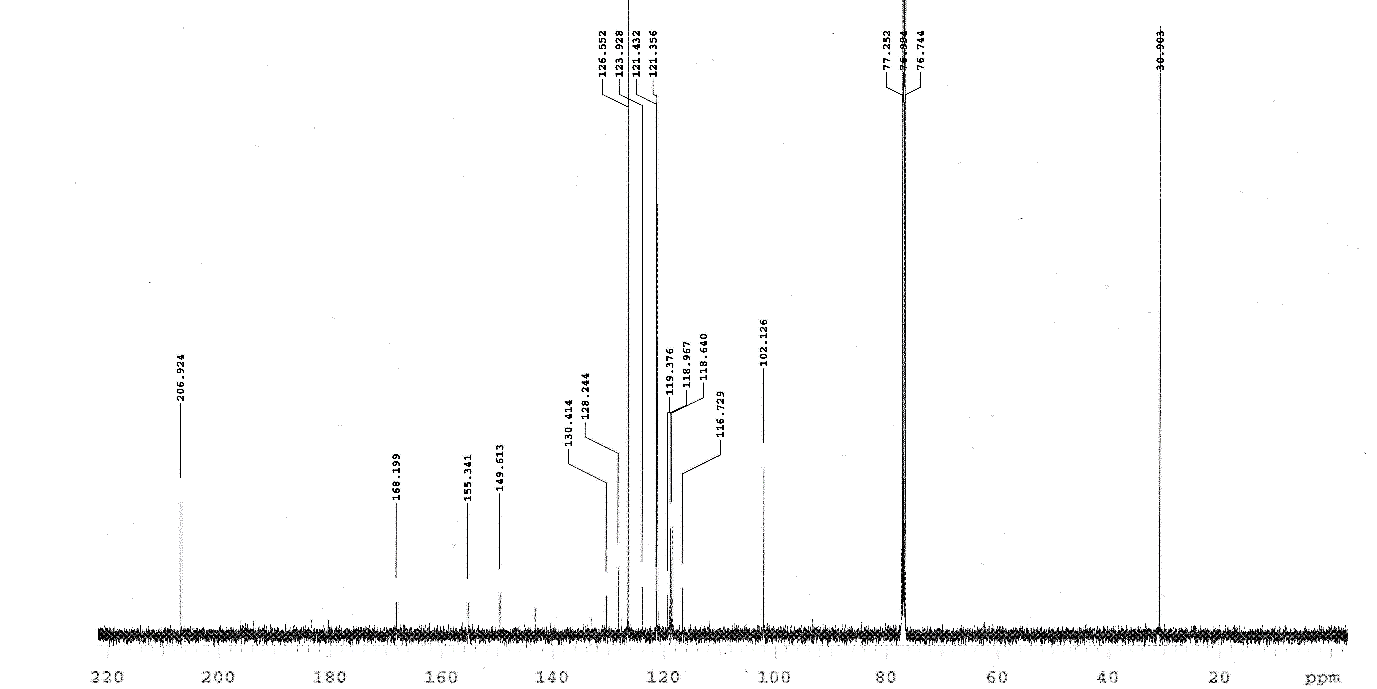
**

**Figure S1.5:** ^1^H- and ^13^C-NMR spectra of **6e** in CDCl_3_ at 500 MHz and 125 MHz, respectively.


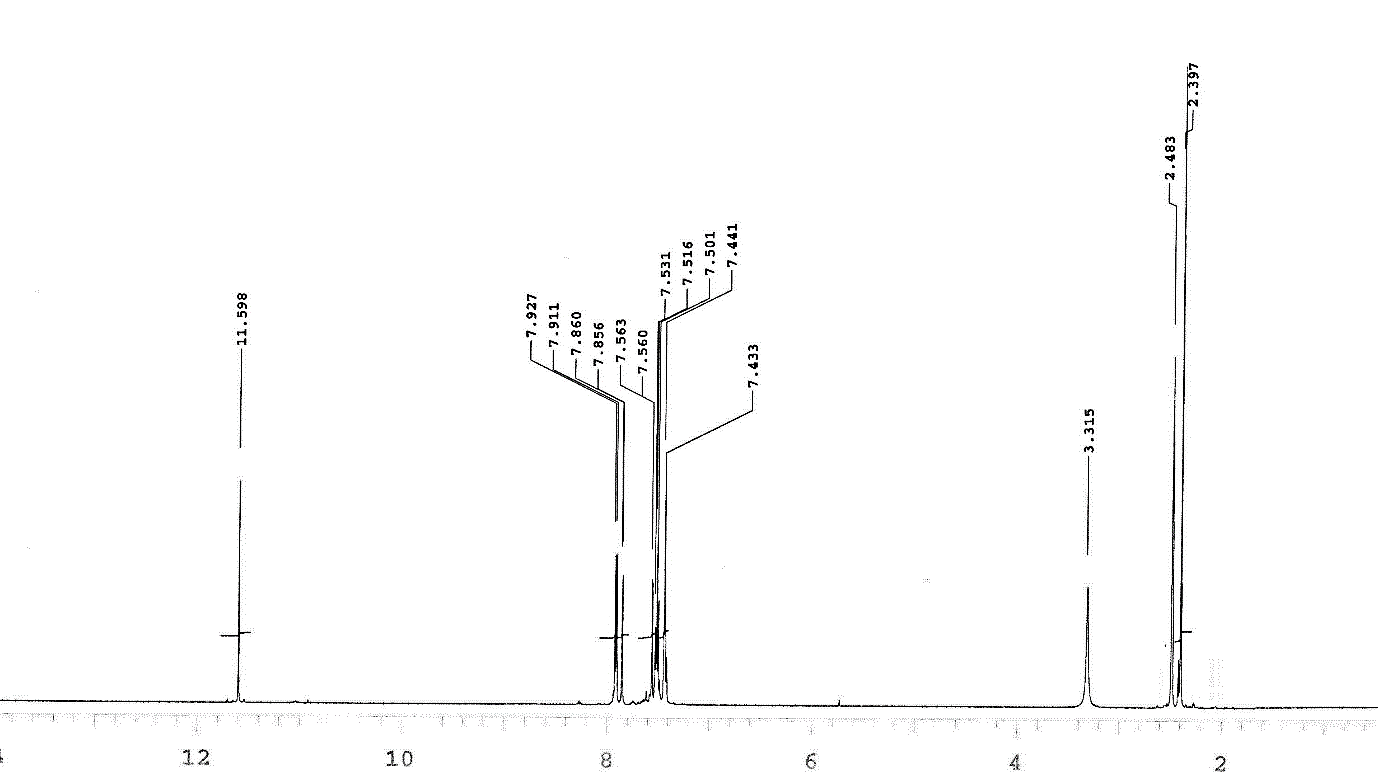

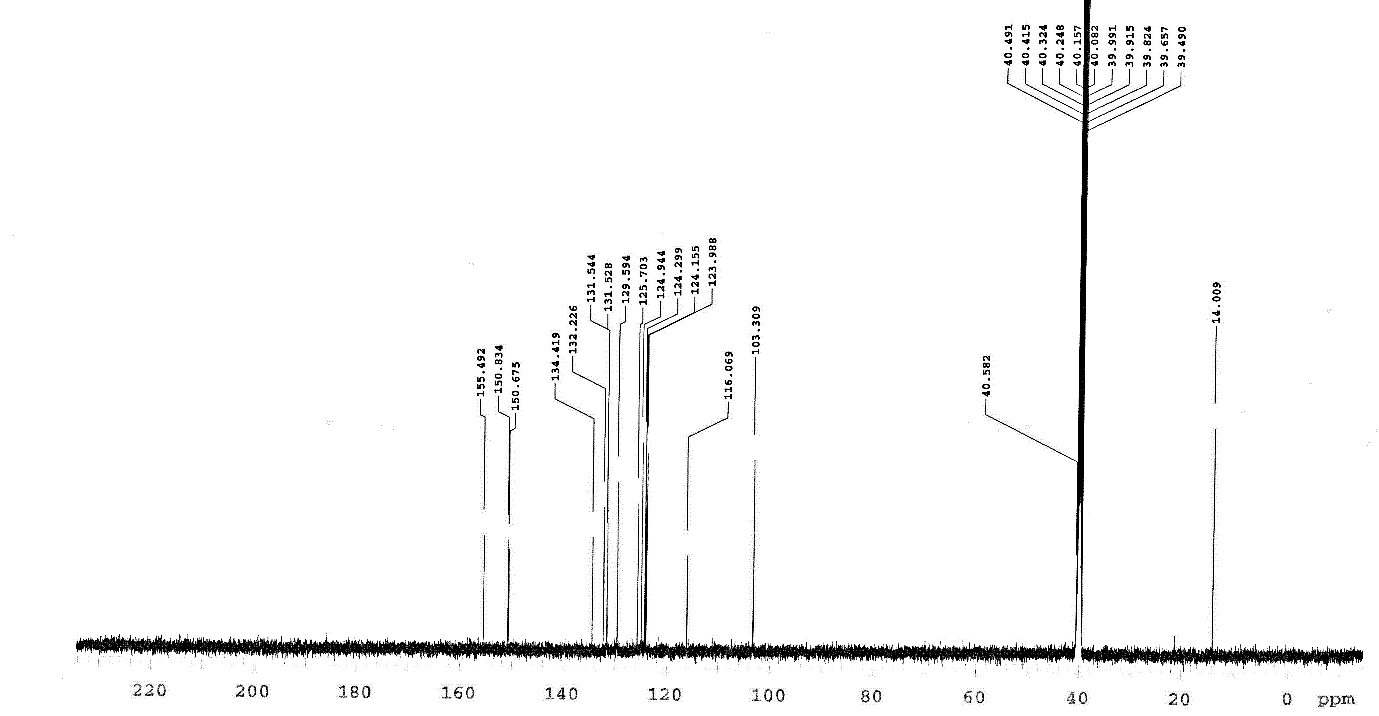


**Figure S1.6:** ^1^H- and ^13^C-NMR spectra of **7a** in DMSO-*d_6_* at 500 MHz and 125 MHz, respectively.

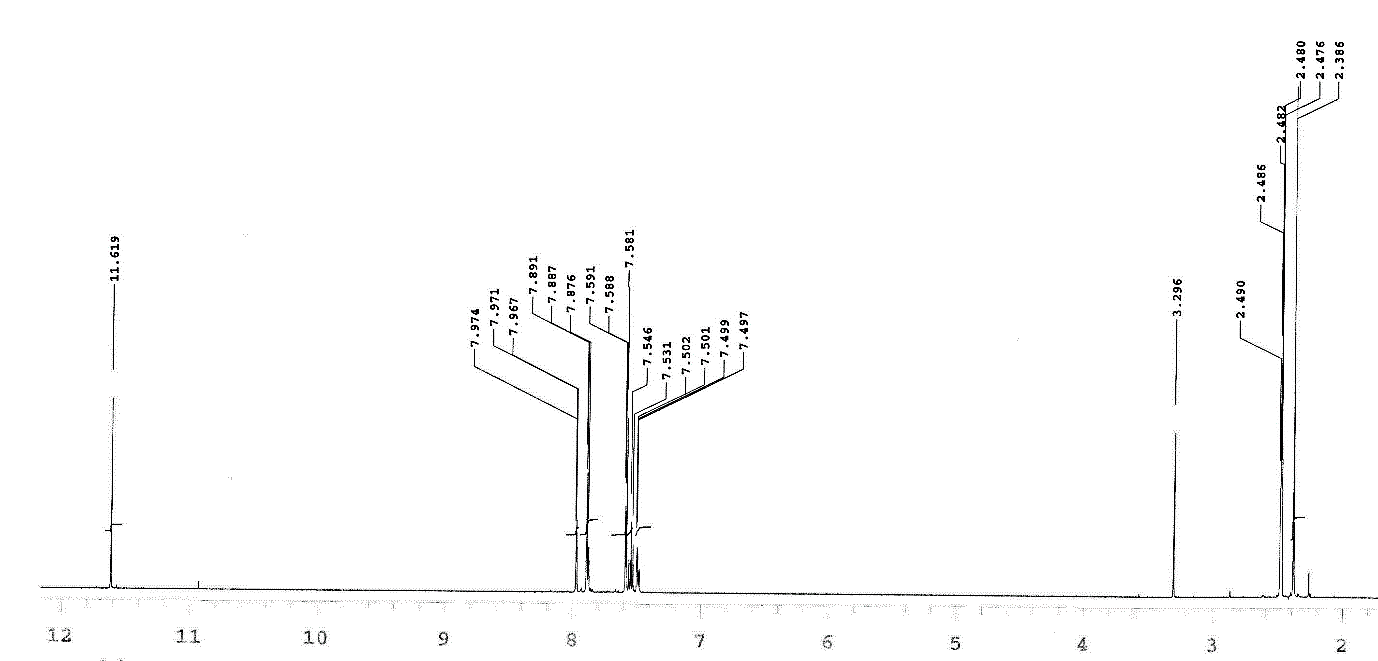


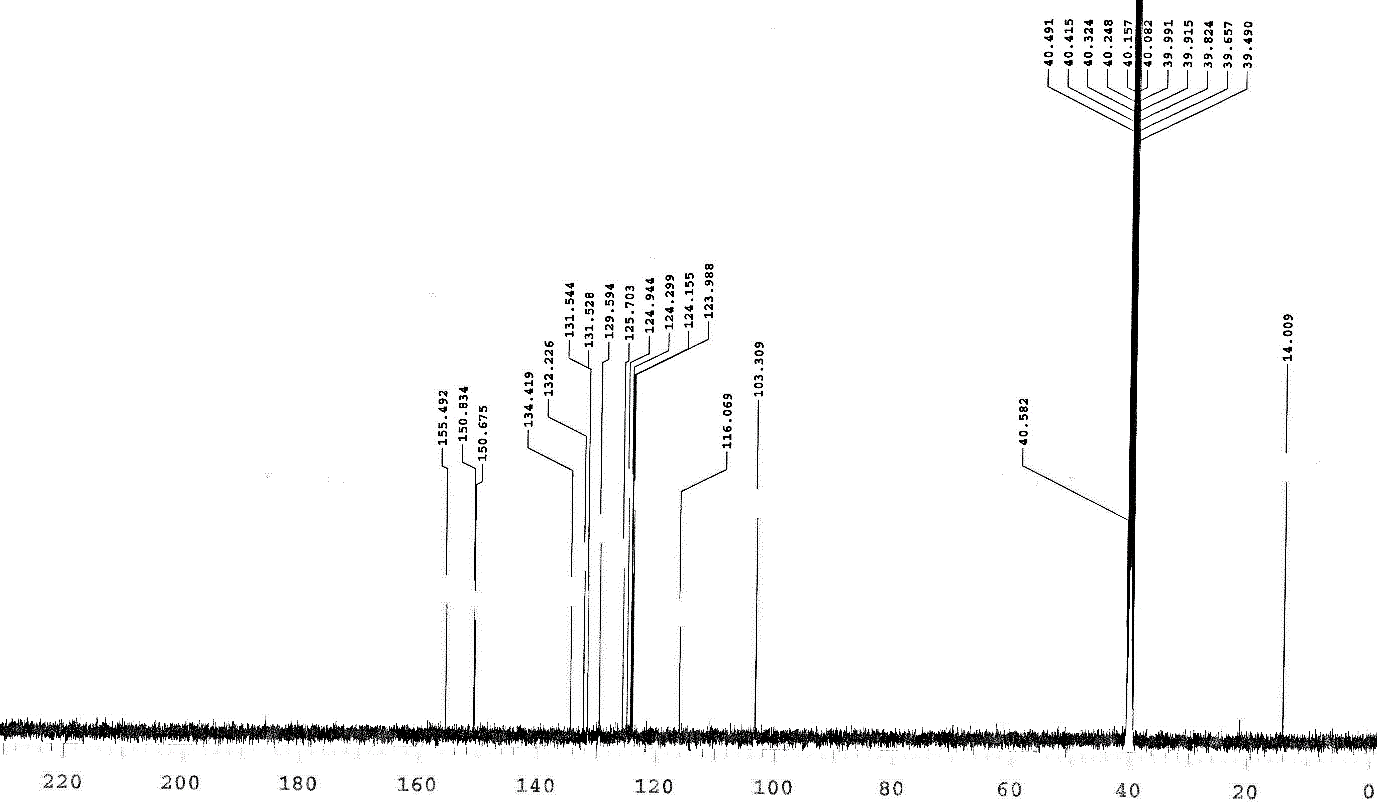


**Figure S1.7:** ^1^H- and ^13^C-NMR spectra of **7b** in DMSO-*d_6_* at 500 MHz and 125 MHz, respectively.


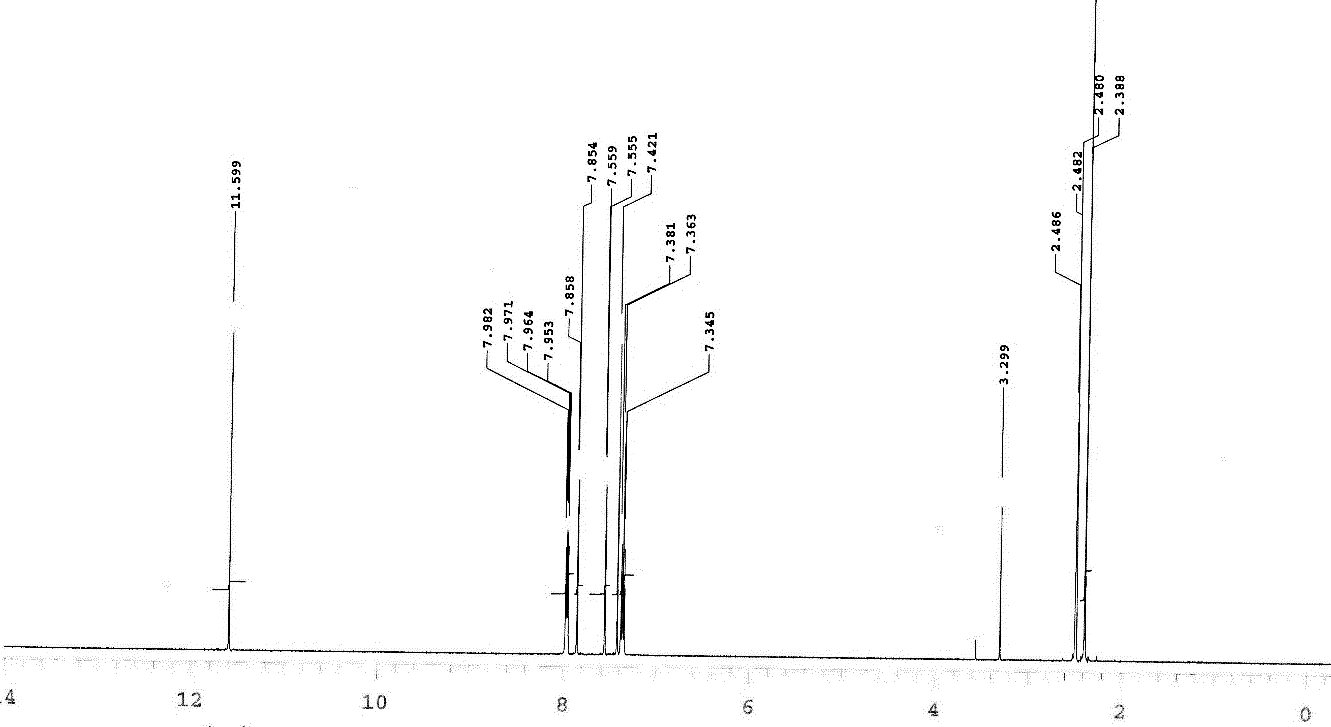

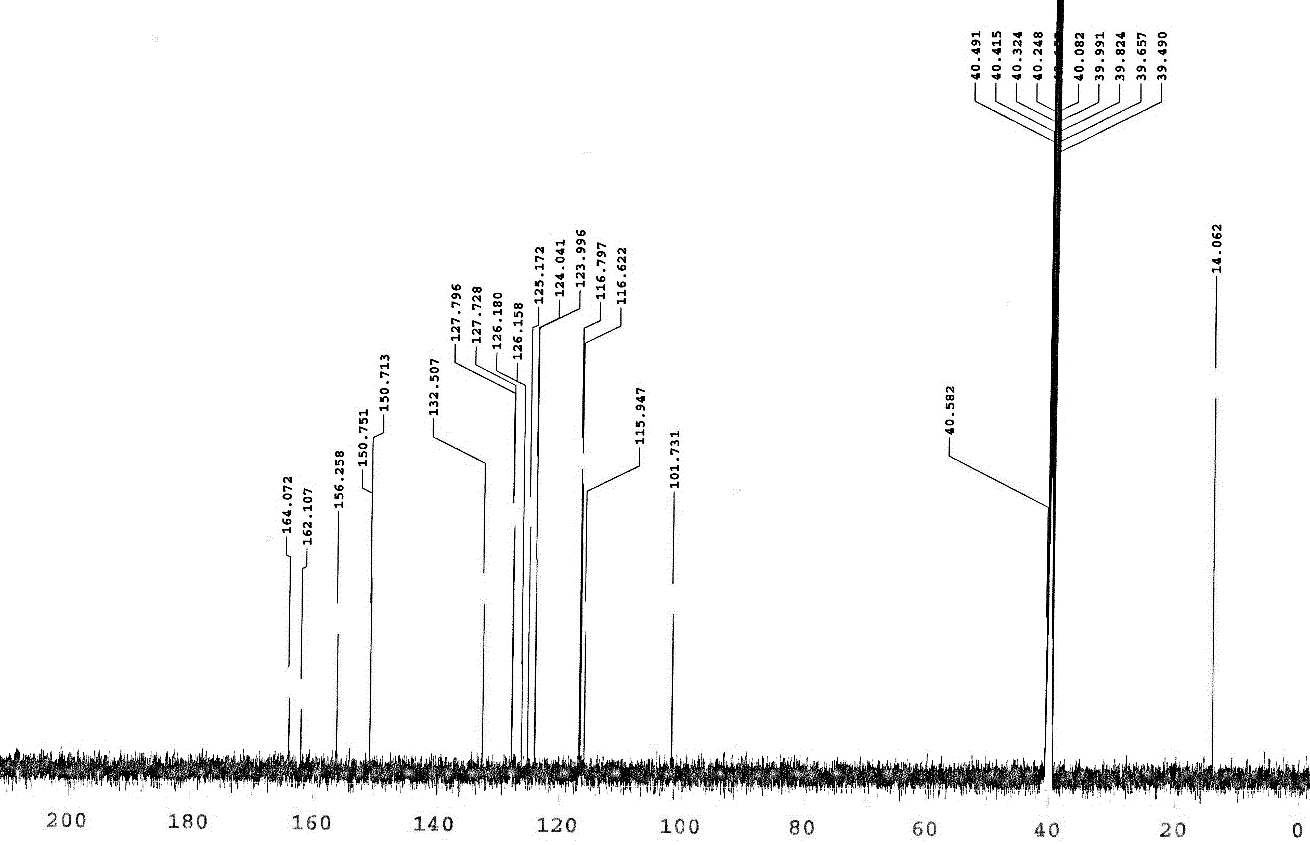


**Figure S1.8:** ^1^H- and ^13^C-NMR spectra of **7c** in DMSO-*d_6_* at 500 MHz and 125 MHz, respectively.


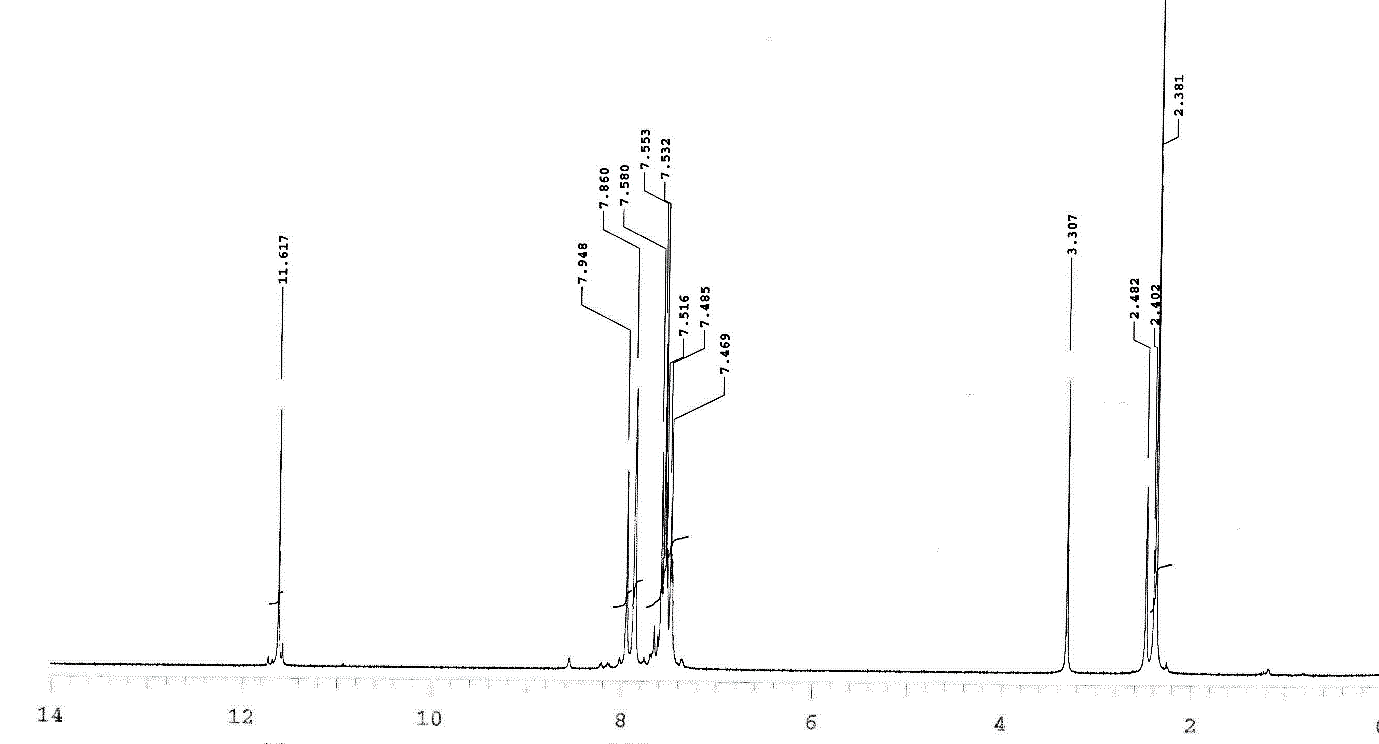

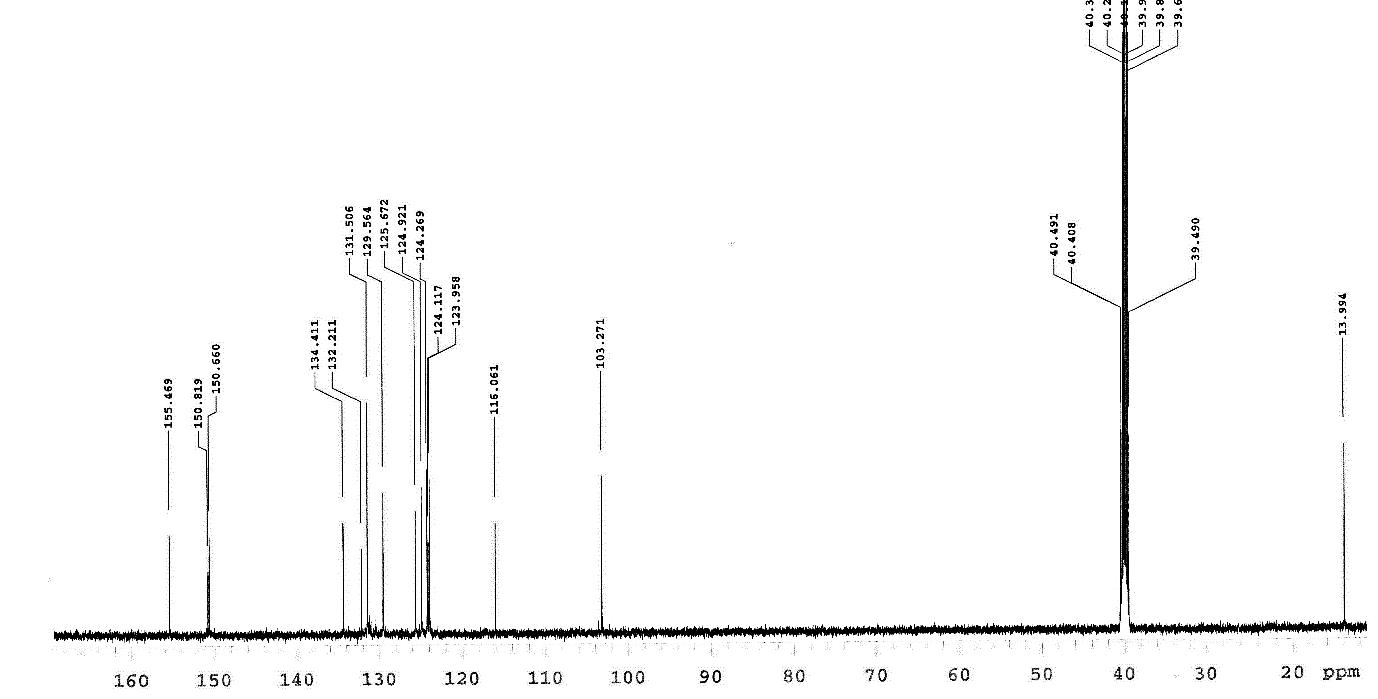


**Figure S1.9:** ^1^H- and ^13^C-NMR spectra of **7d** in DMSO-*d_6_* at 500 MHz and 125 MHz, respectively.


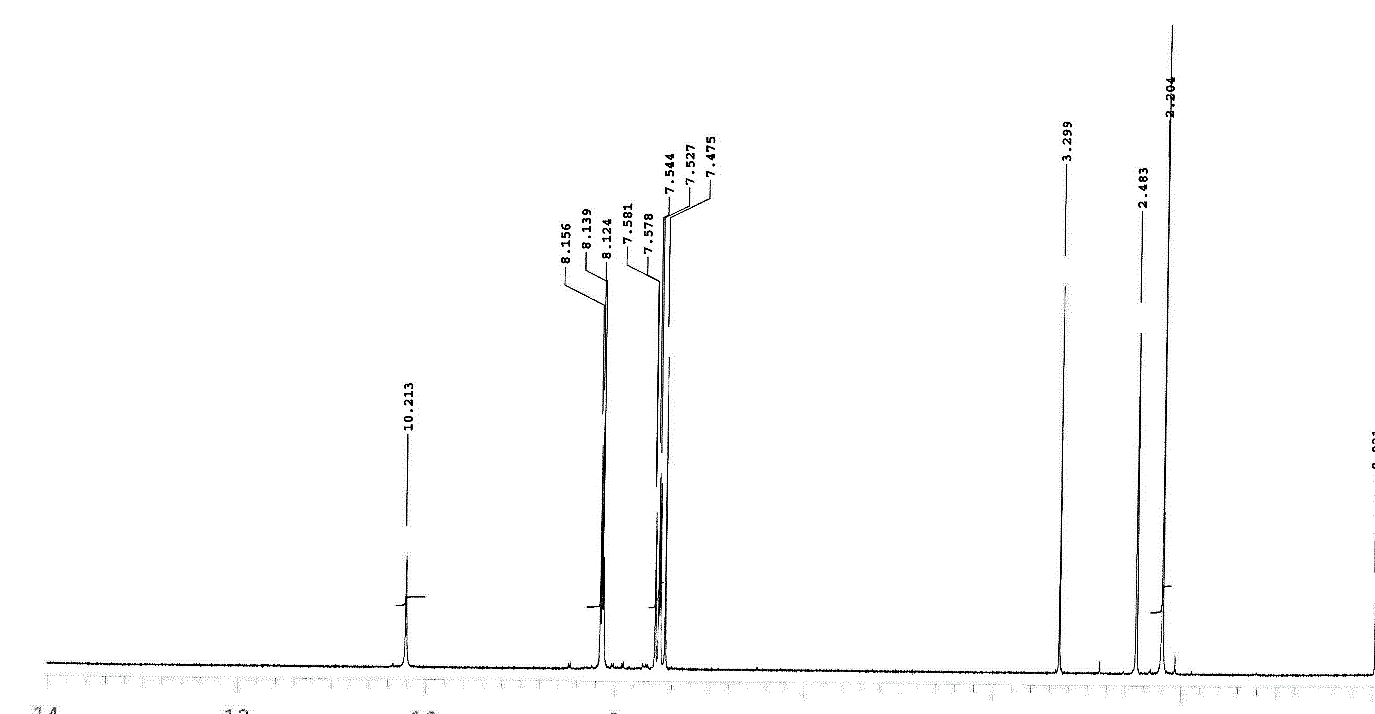

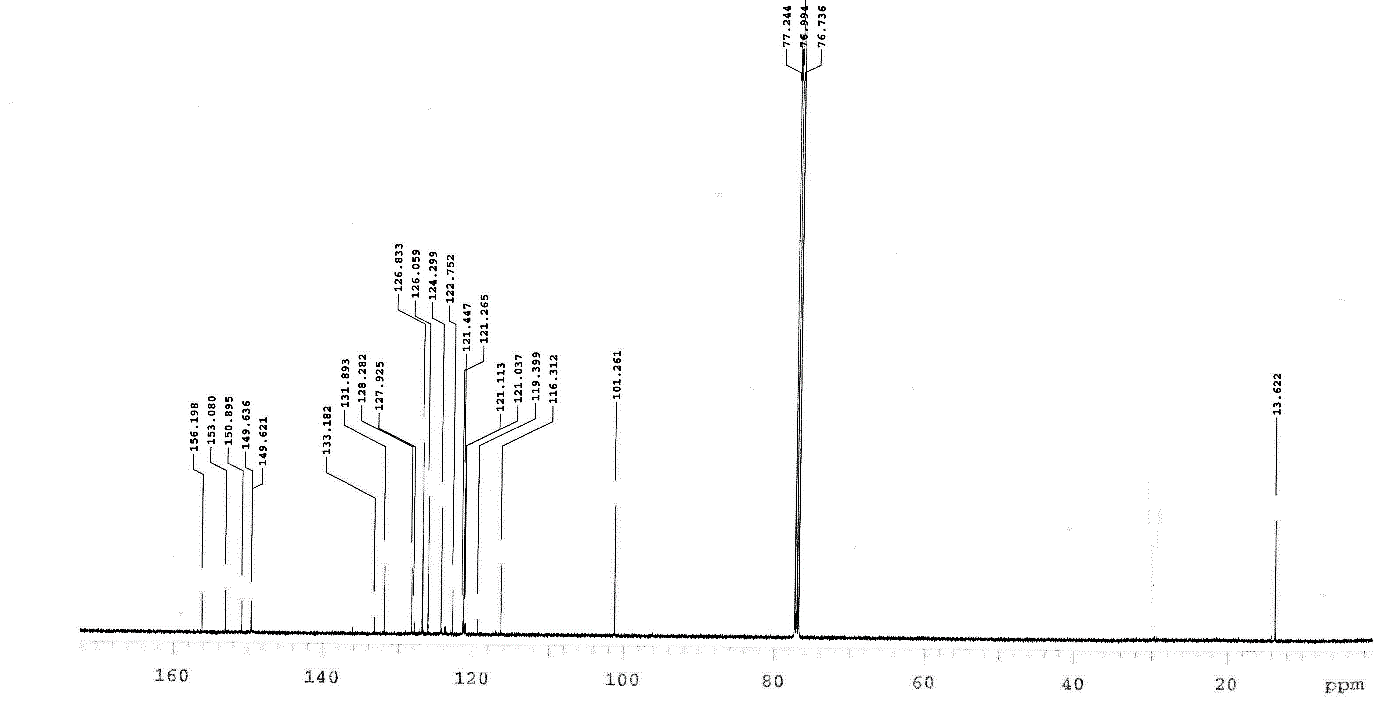


**Figure S1.10:** ^1^H- and ^13^C-NMR spectra of **7e** in DMSO-*d_6_* at 500 MHz and 125 MHz, respectively.


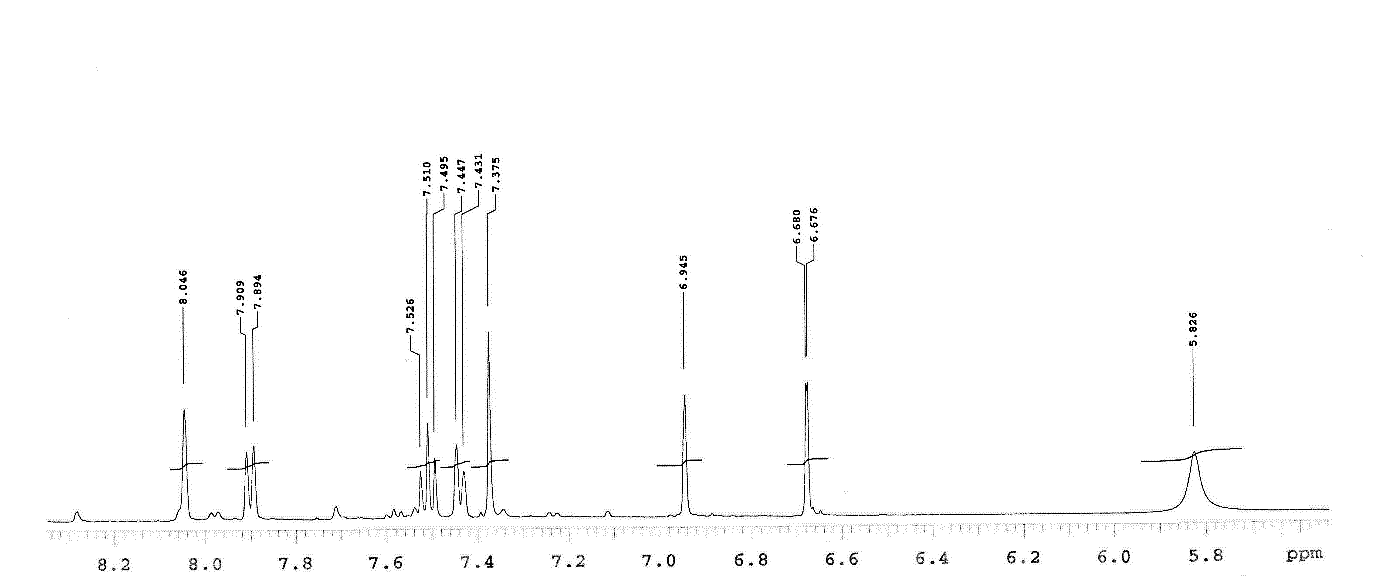

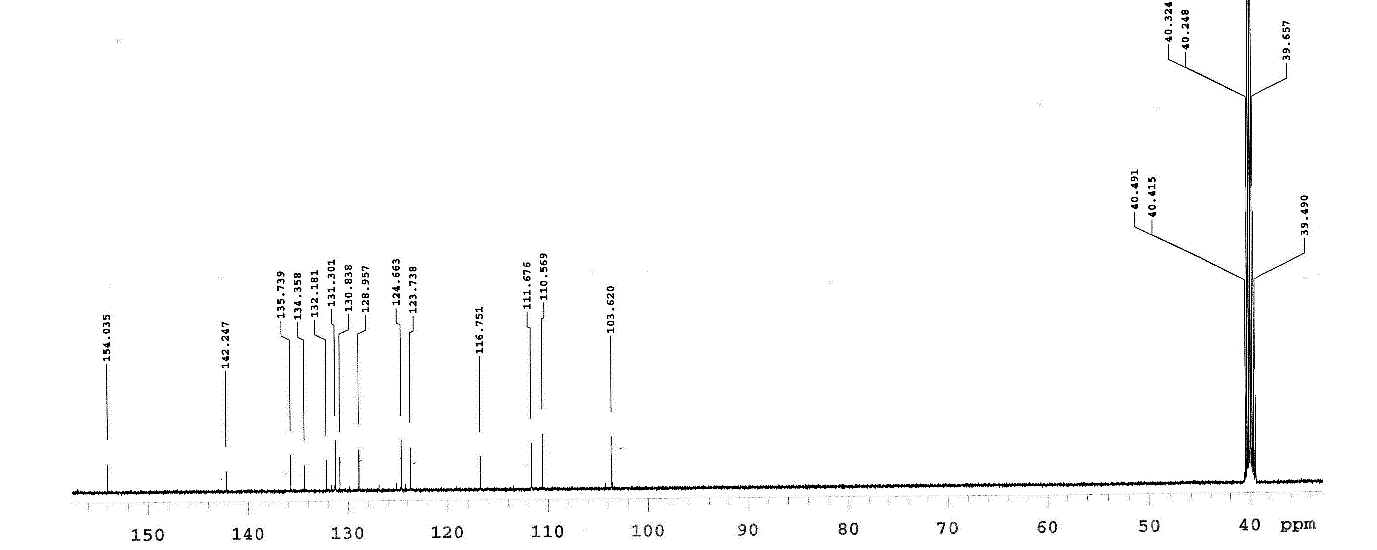


**Figure S1.11:** ^1^H- and ^13^C-NMR spectra of **8a** in DMSO-*d_6_* at 500 MHz and 125 MHz, respectively.


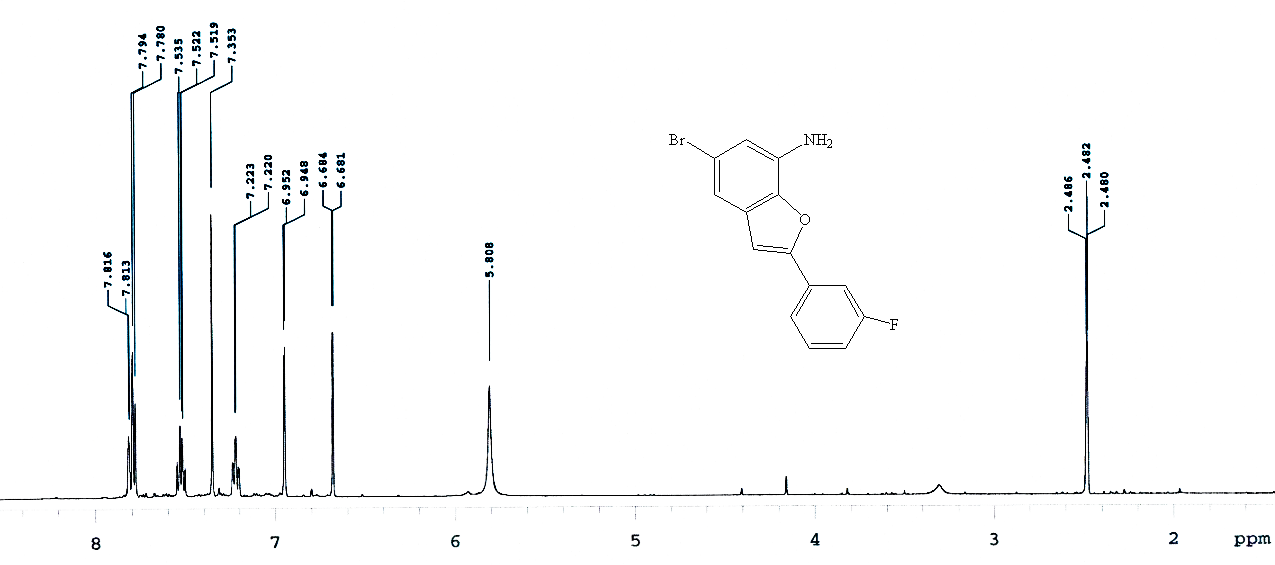


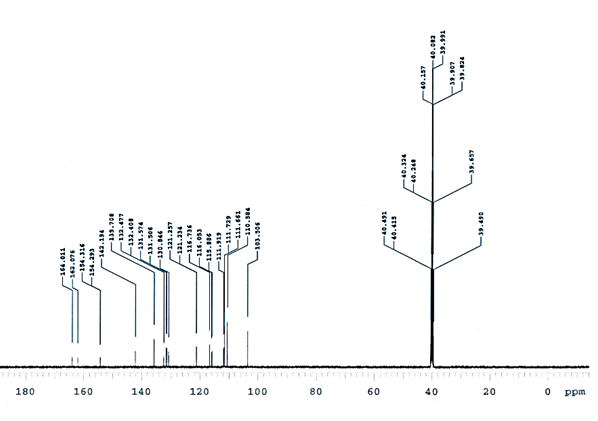


**Figure S1.12:** ^1^H- and ^13^C-NMR spectra of **8b** in DMSO-*d_6_* at 500 MHz and 125 MHz, respectively.


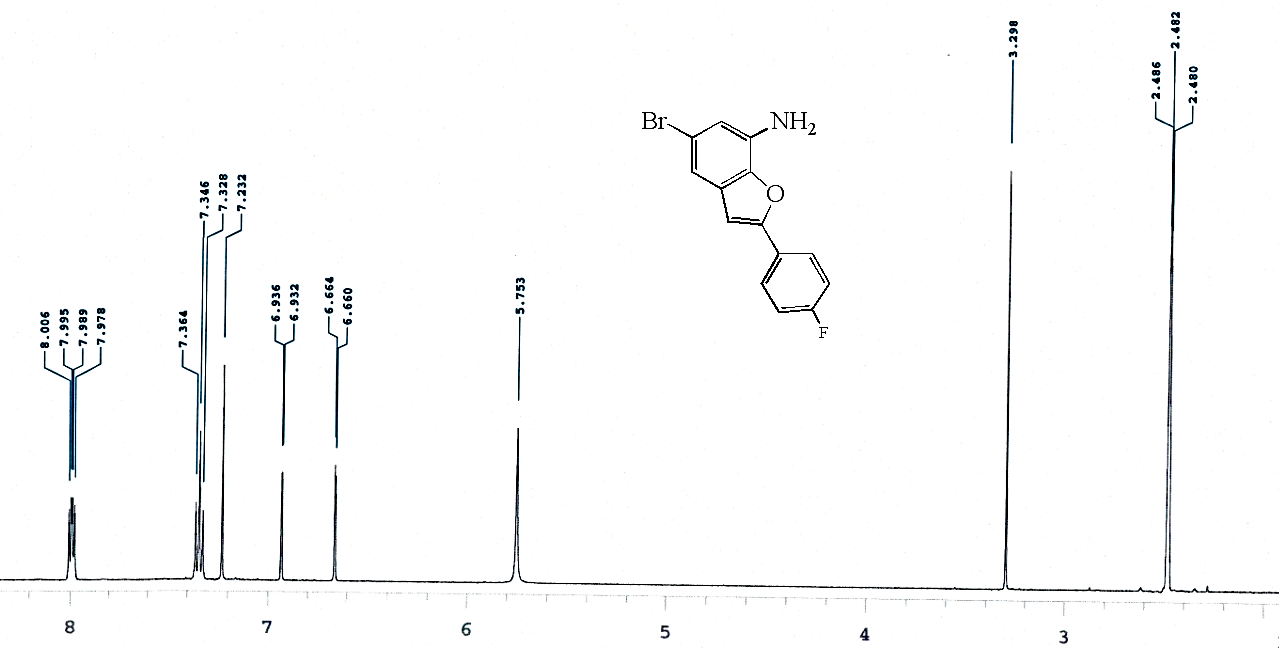

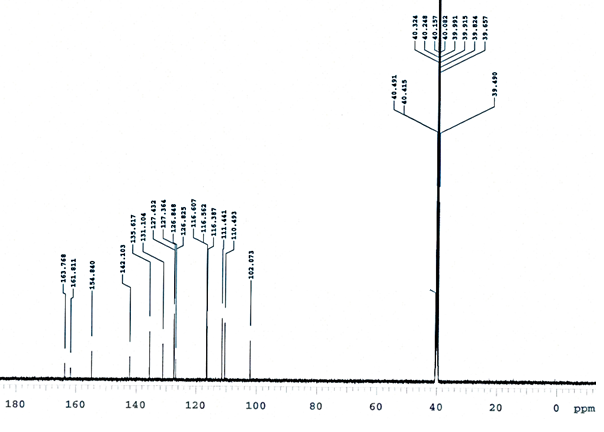


**Figure S1.13:** ^1^H- and ^13^C-NMR spectra of **8c** in DMSO-*d_6_* at 500 MHz and 125 MHz, respectively.


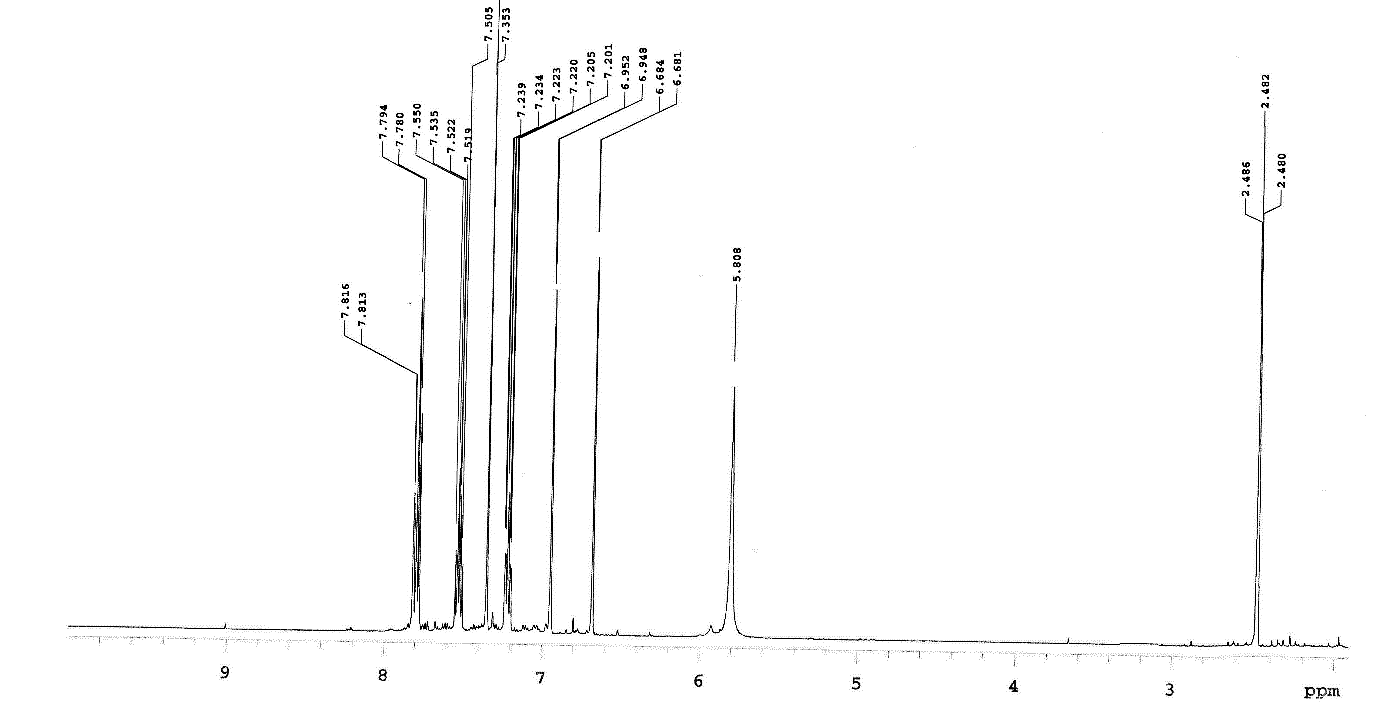

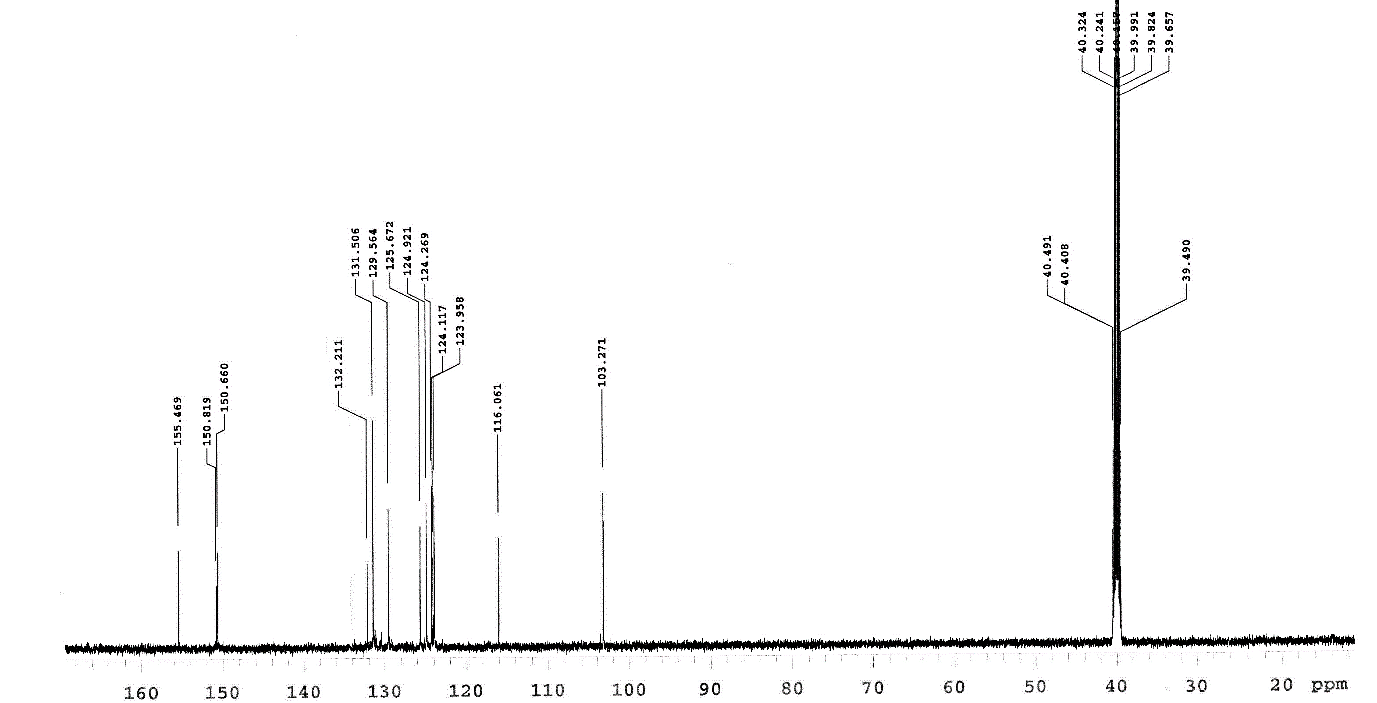


**Figure S1.14:** ^1^H- and ^13^C-NMR spectra of **8d** in DMSO-*d_6_* at 500 MHz and 125 MHz, respectively.


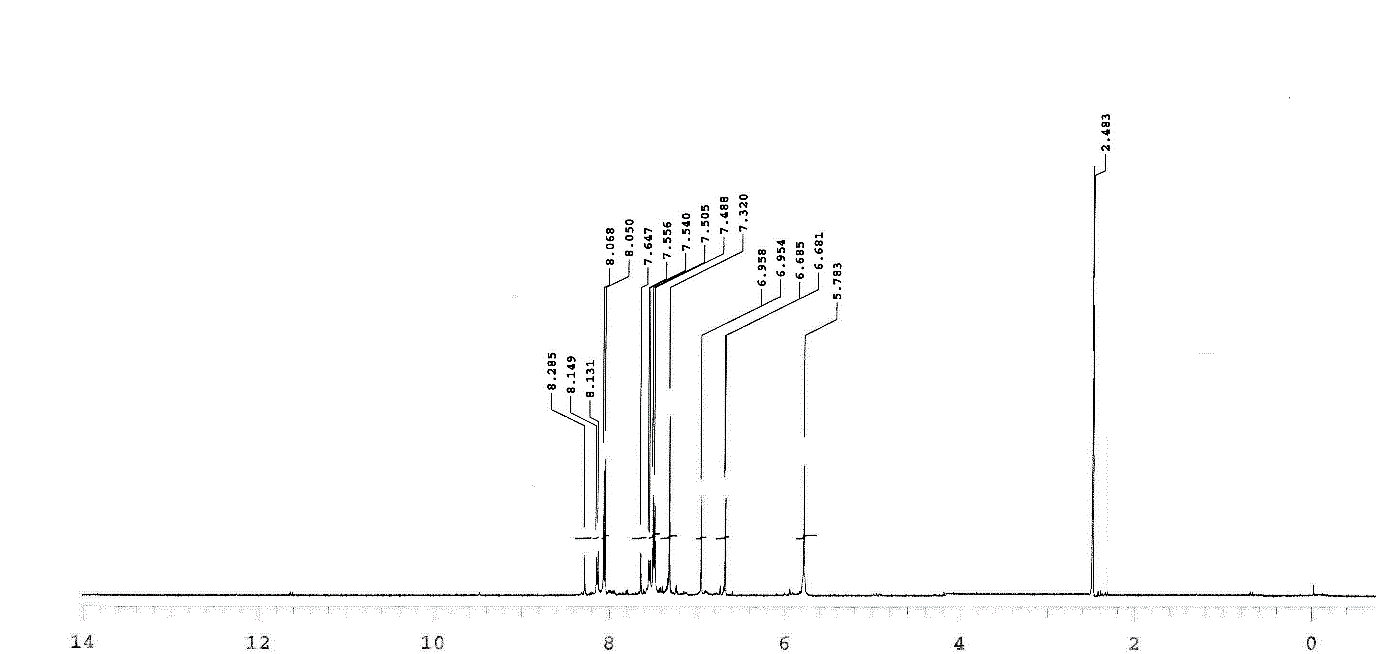

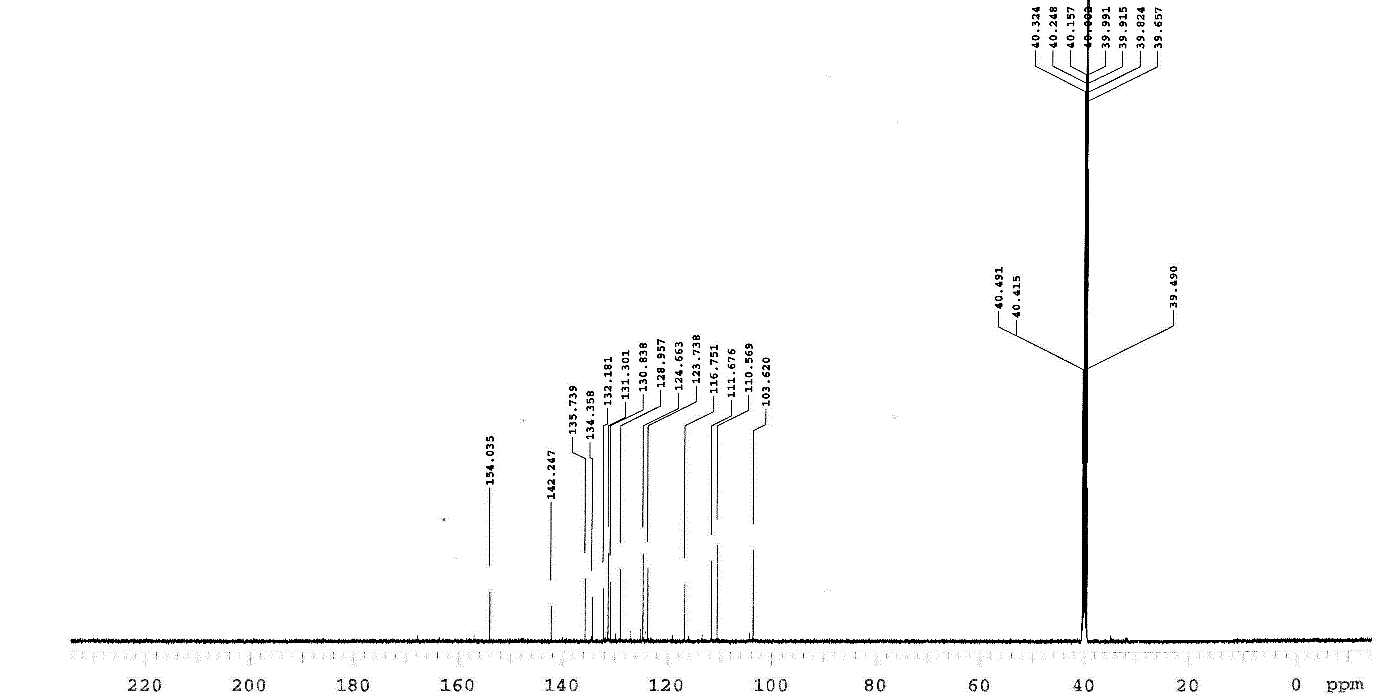


**Figure S1.15:** ^1^H- and ^13^C-NMR spectra of **8e** in DMSO-*d_6_* at 500 MHz and 125 MHz, respectively.


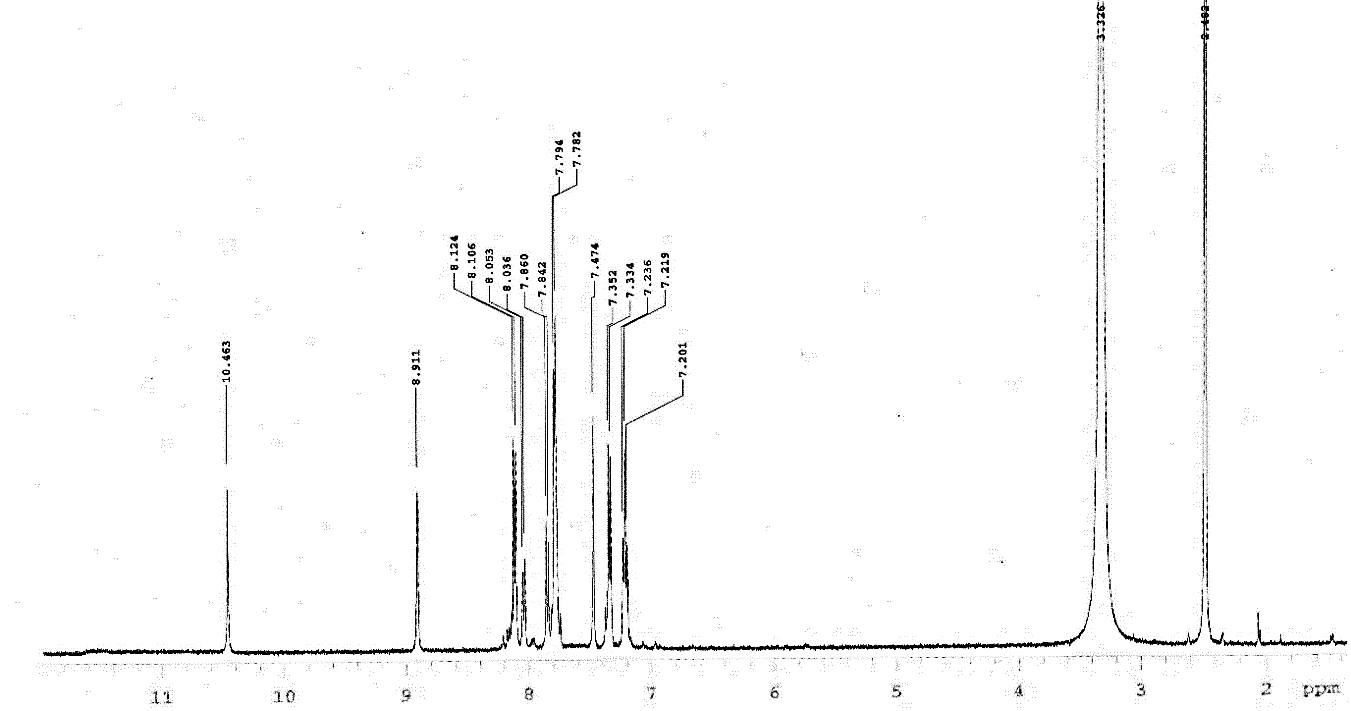

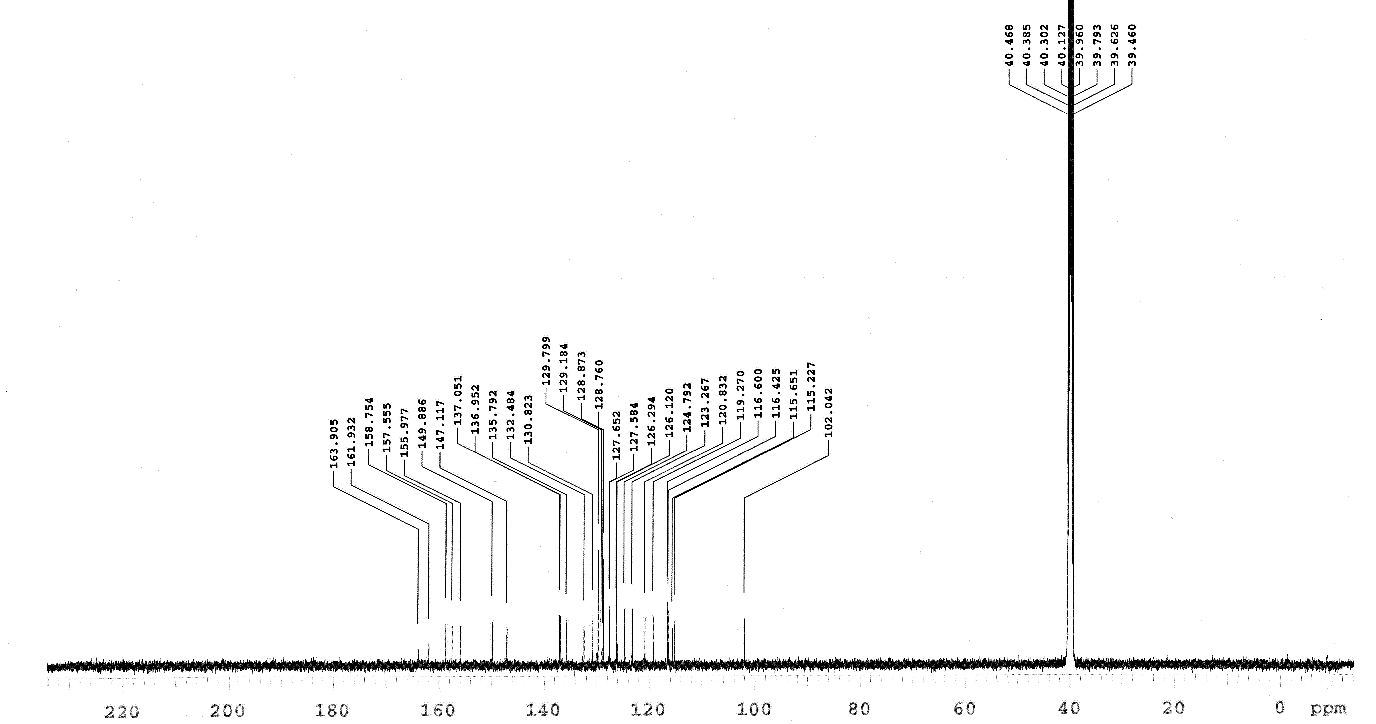

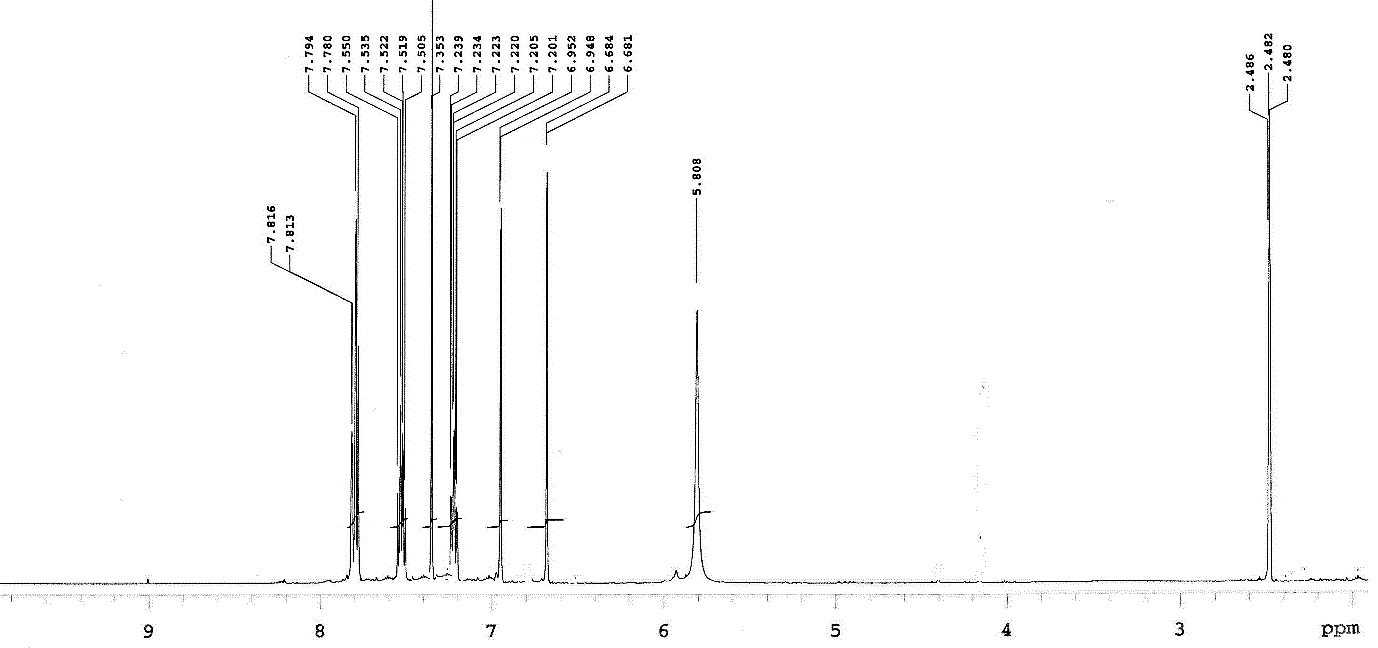


**Figure S1.16:** ^1^H- and ^13^C-NMR spectra of **10a** in DMSO-*d_6_* at 500 MHz and 125 MHz, respectively.


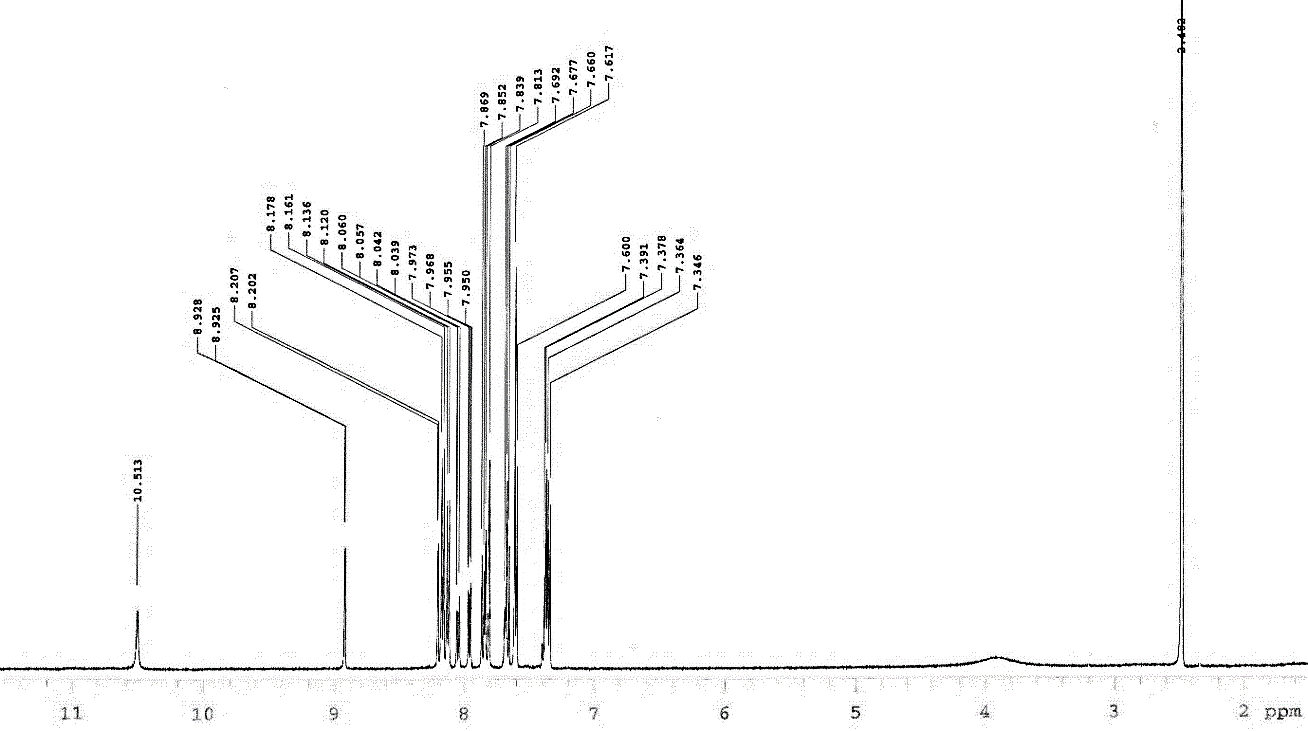

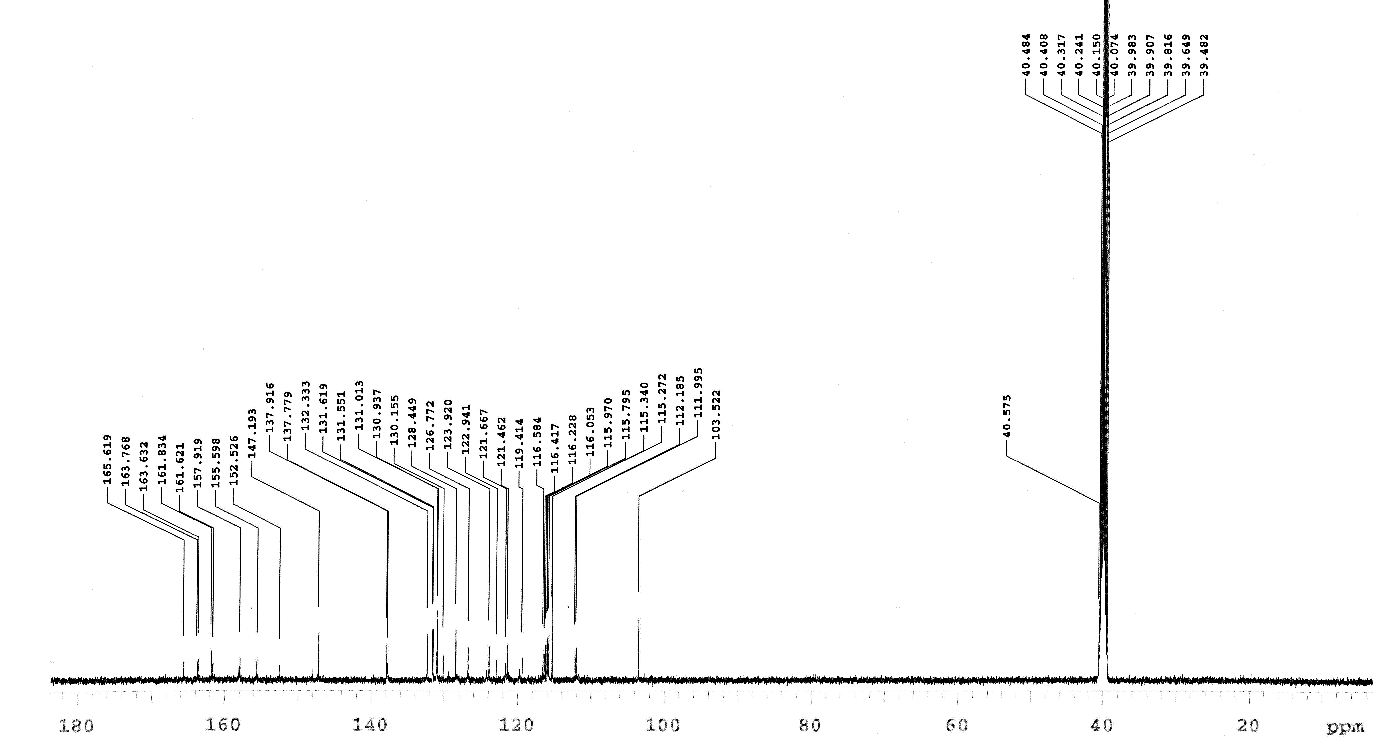


**Figure S1.17:** ^1^H- and ^13^C-NMR spectra of **10b** in DMSO-*d_6_* at 500 MHz and 125 MHz, respectively.

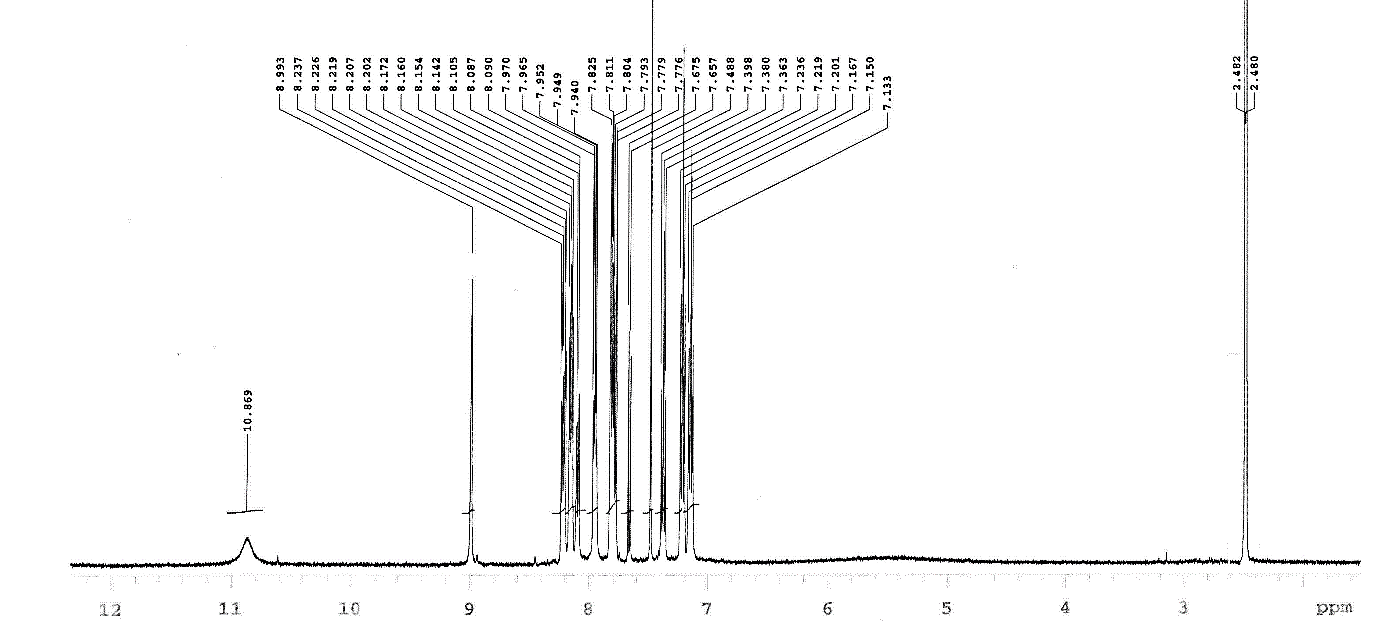


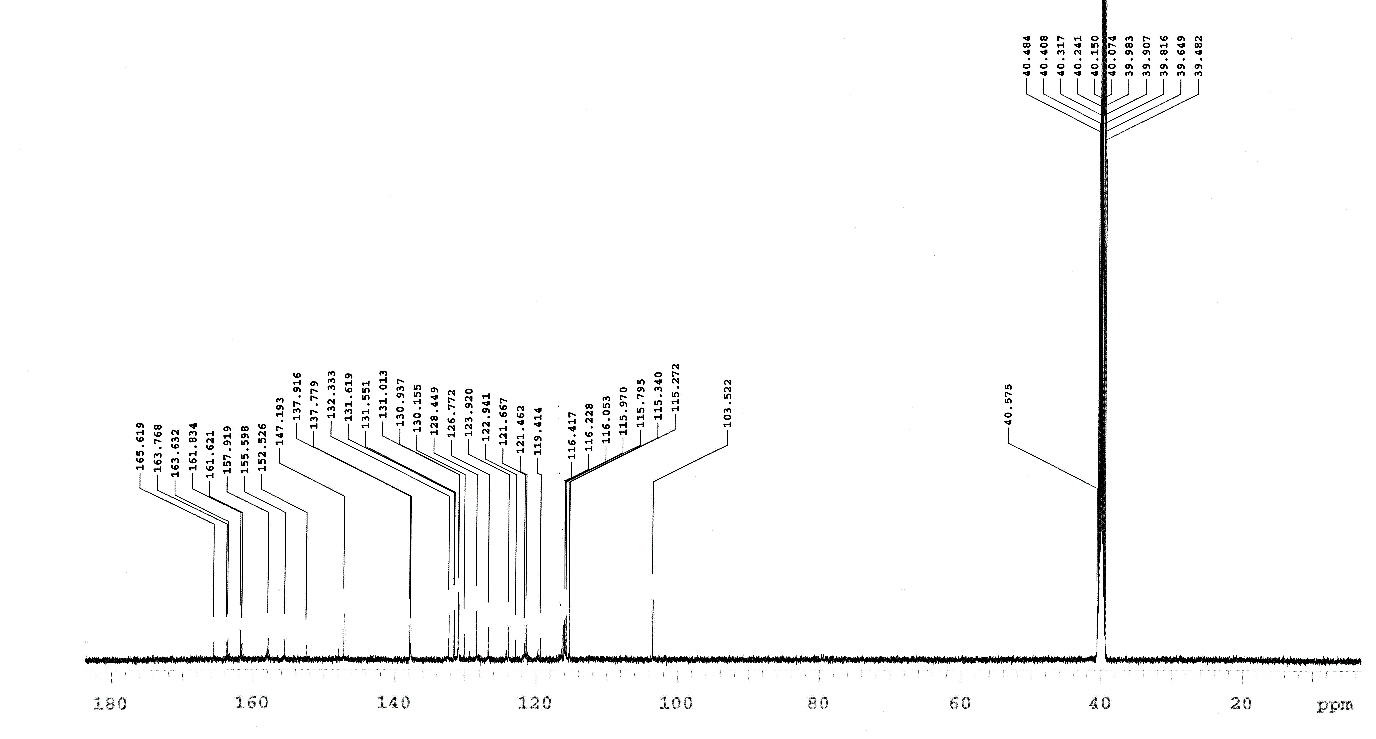


**Figure S1.18:** ^1^H- and ^13^C-NMR spectra of **10c** in DMSO-*d_6_* at 500 MHz and 125 MHz, respectively.


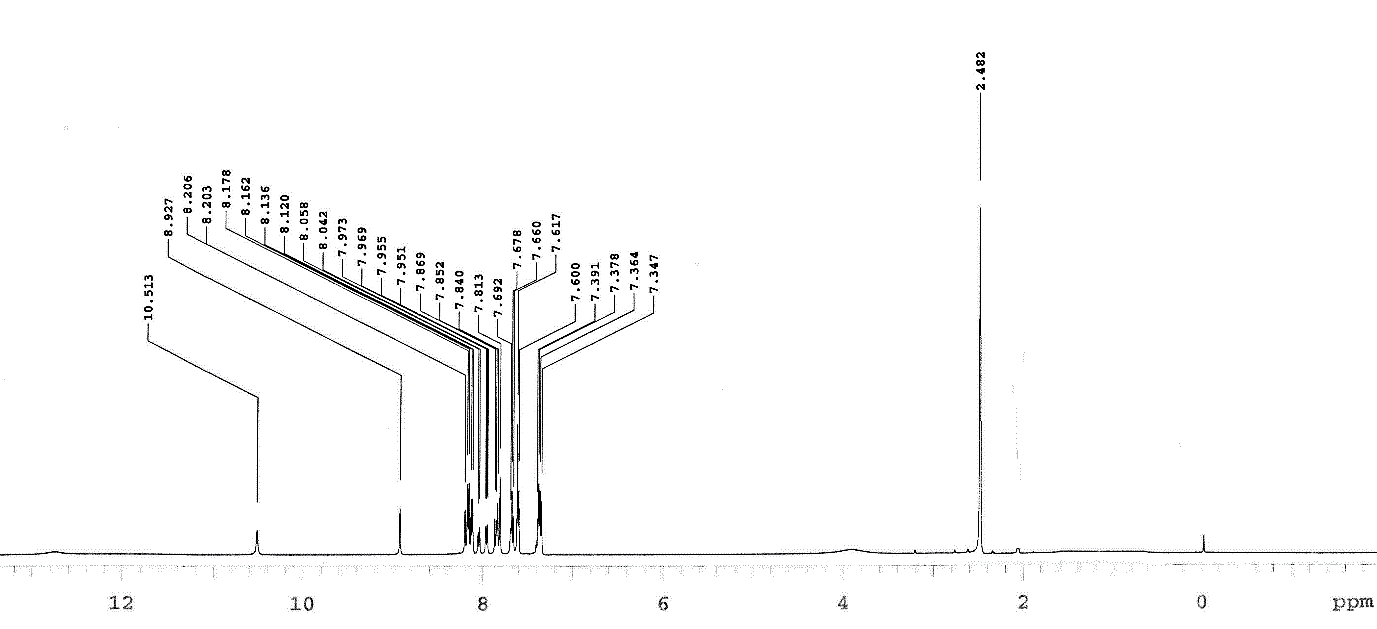

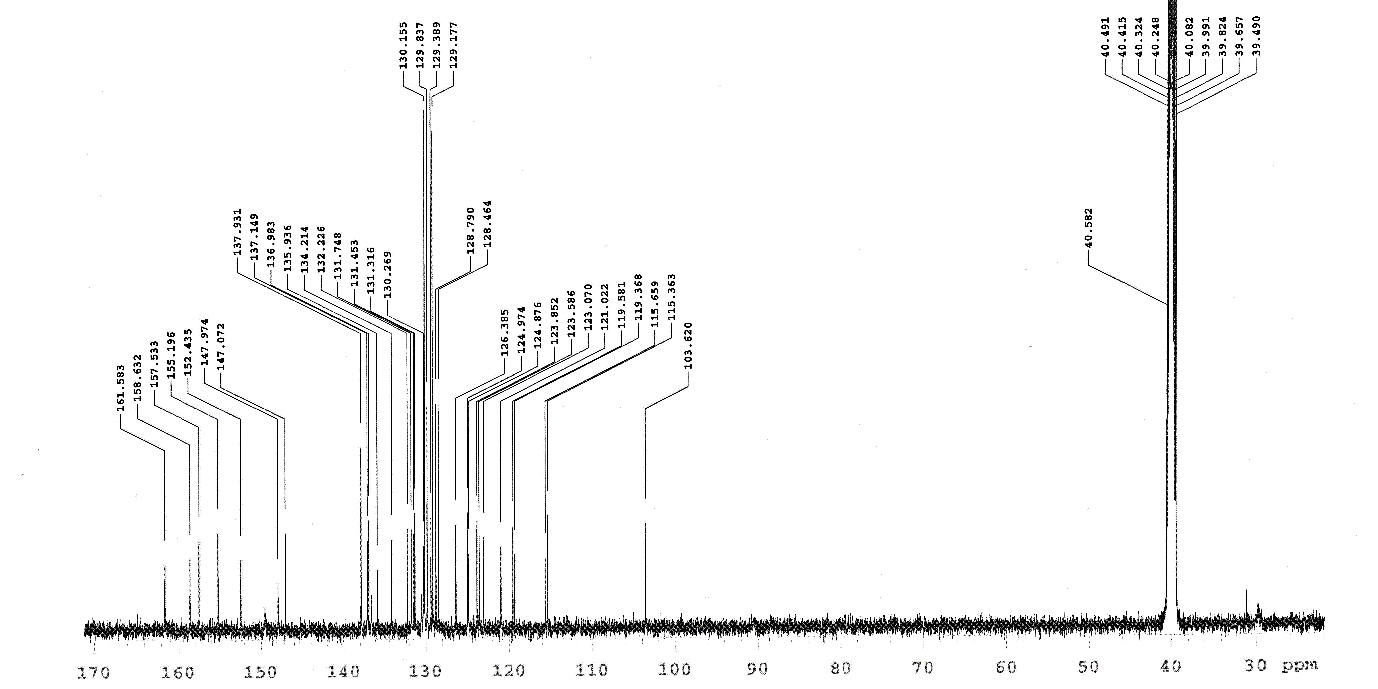


**Figure S1.19:** ^1^H- and ^13^C-NMR spectra of **10d** in DMSO-*d_6_* at 500 MHz and 125 MHz, respectively.


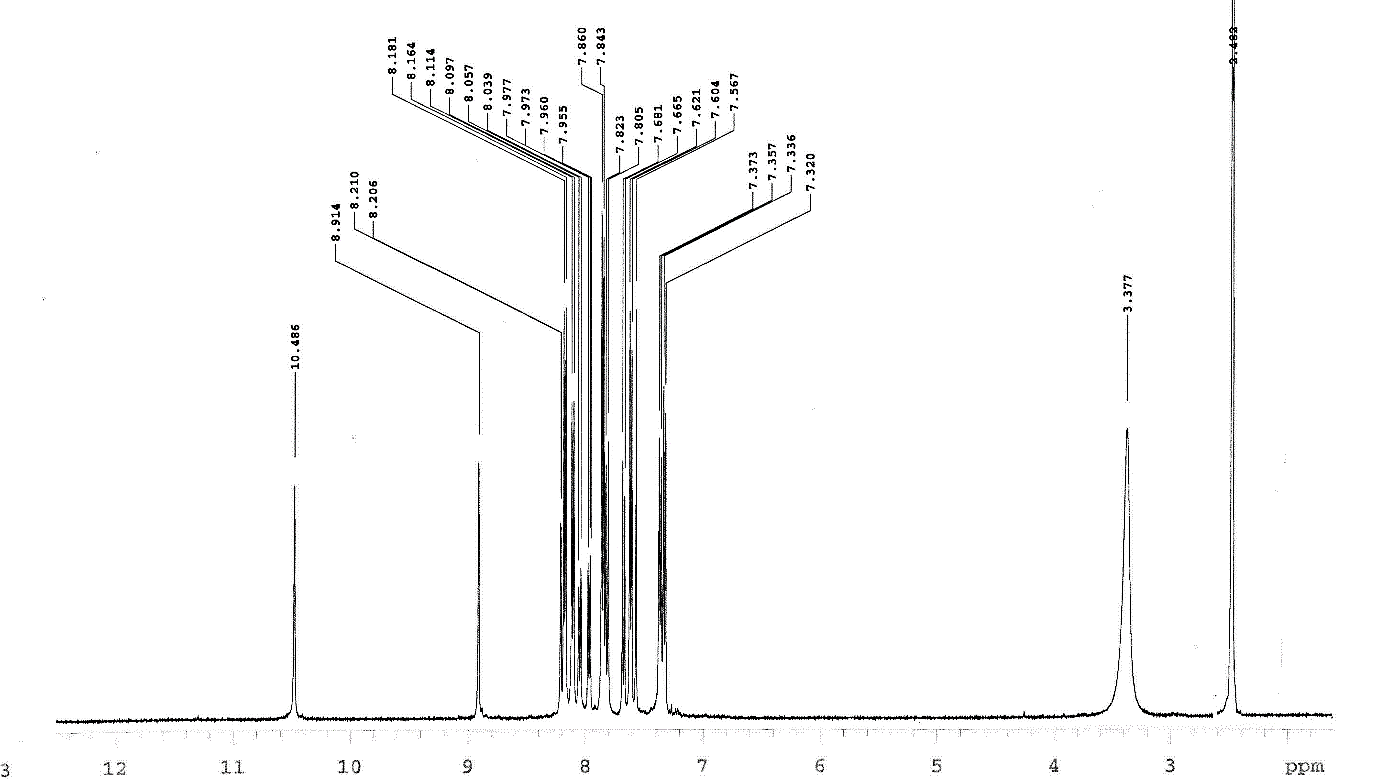

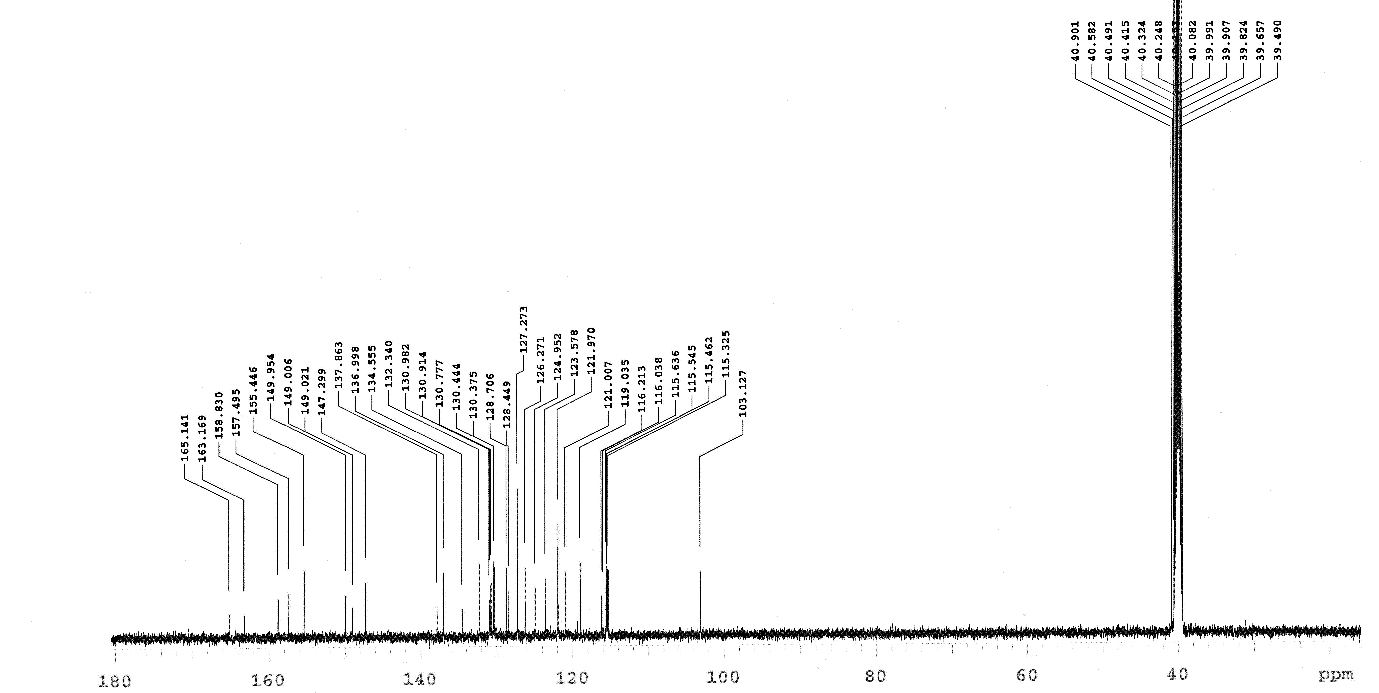


**Figure S1.20:** ^1^H- and ^13^C-NMR spectra of **10e** in DMSO-*d_6_* at 500 MHz and 125 MHz, respectively.

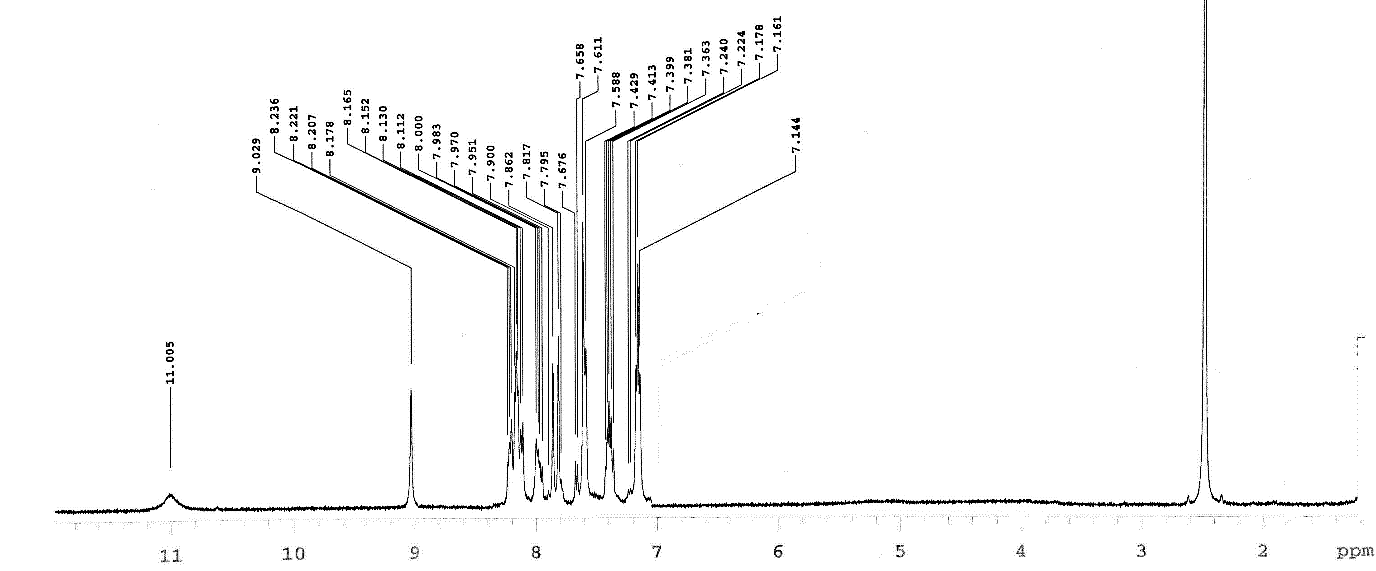


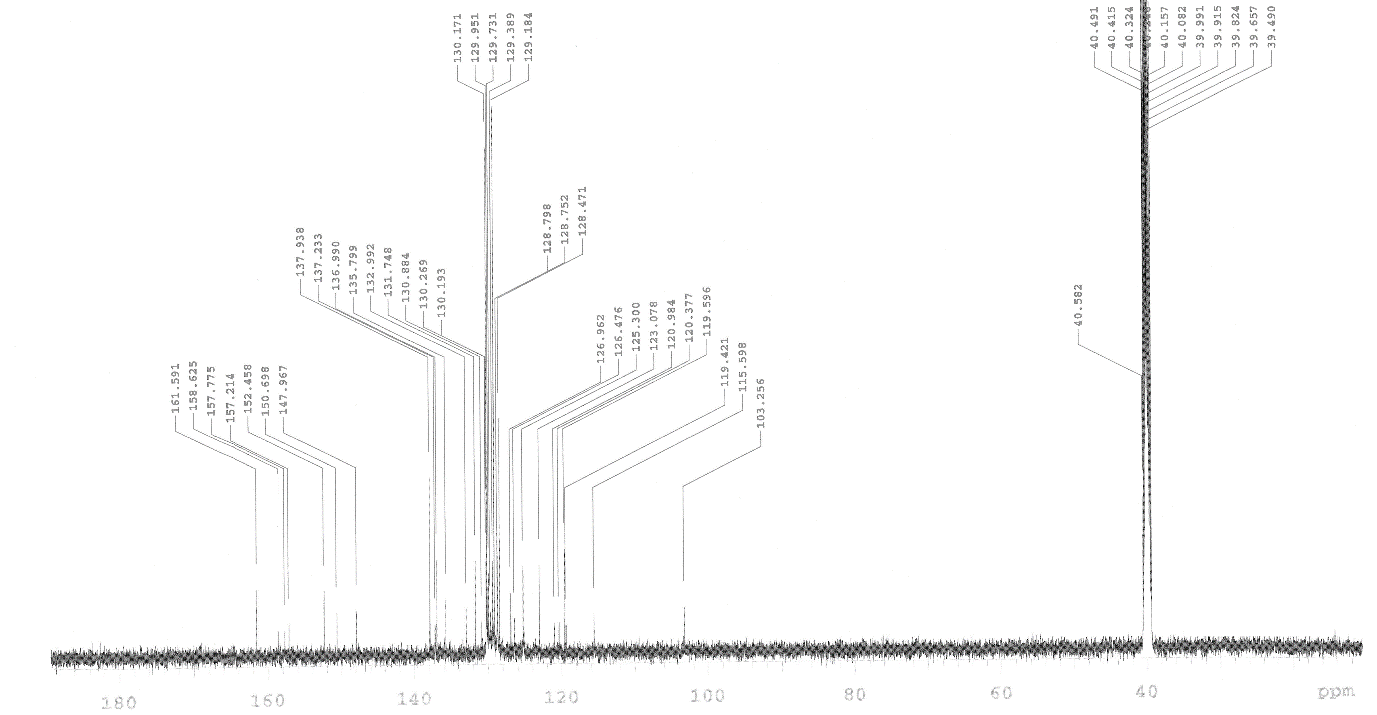


**Figure S1.21:** ^1^H- and ^13^C-NMR spectra of **10f** in DMSO-*d_6_* at 500 MHz and 125 MHz, respectively.

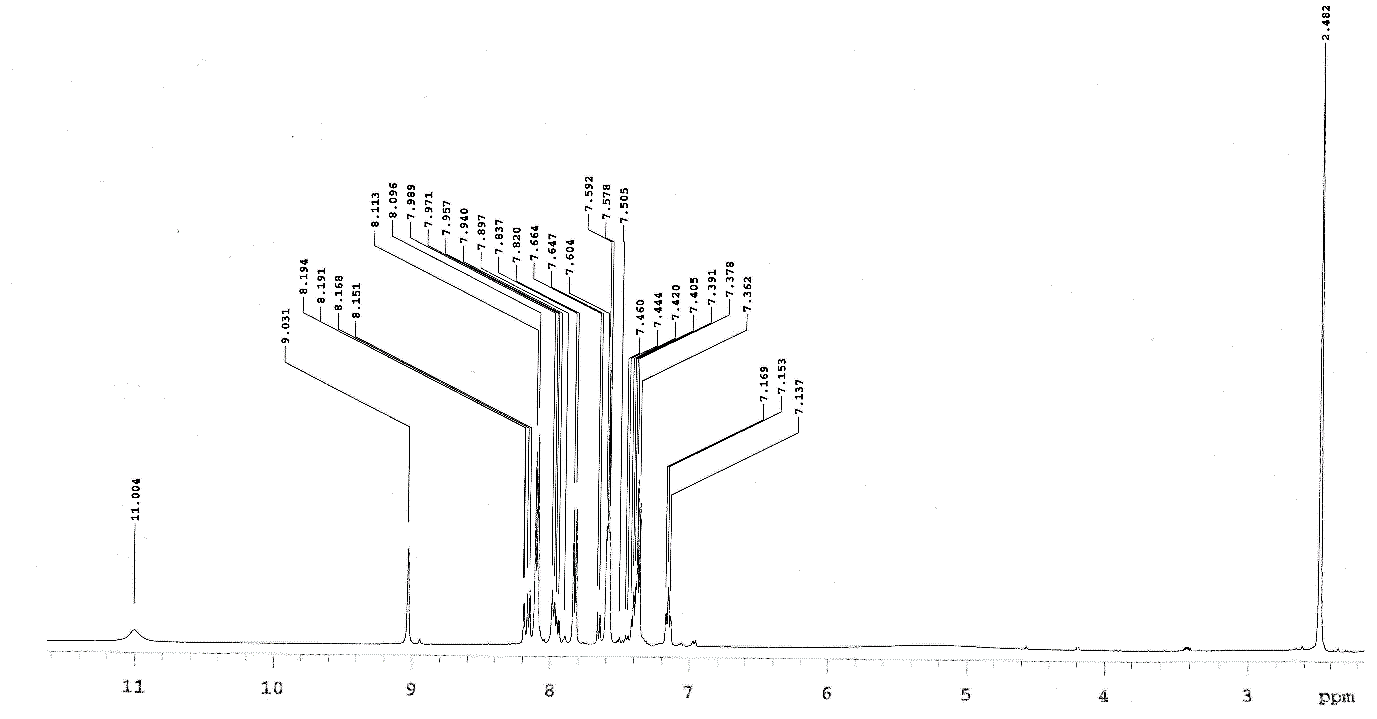


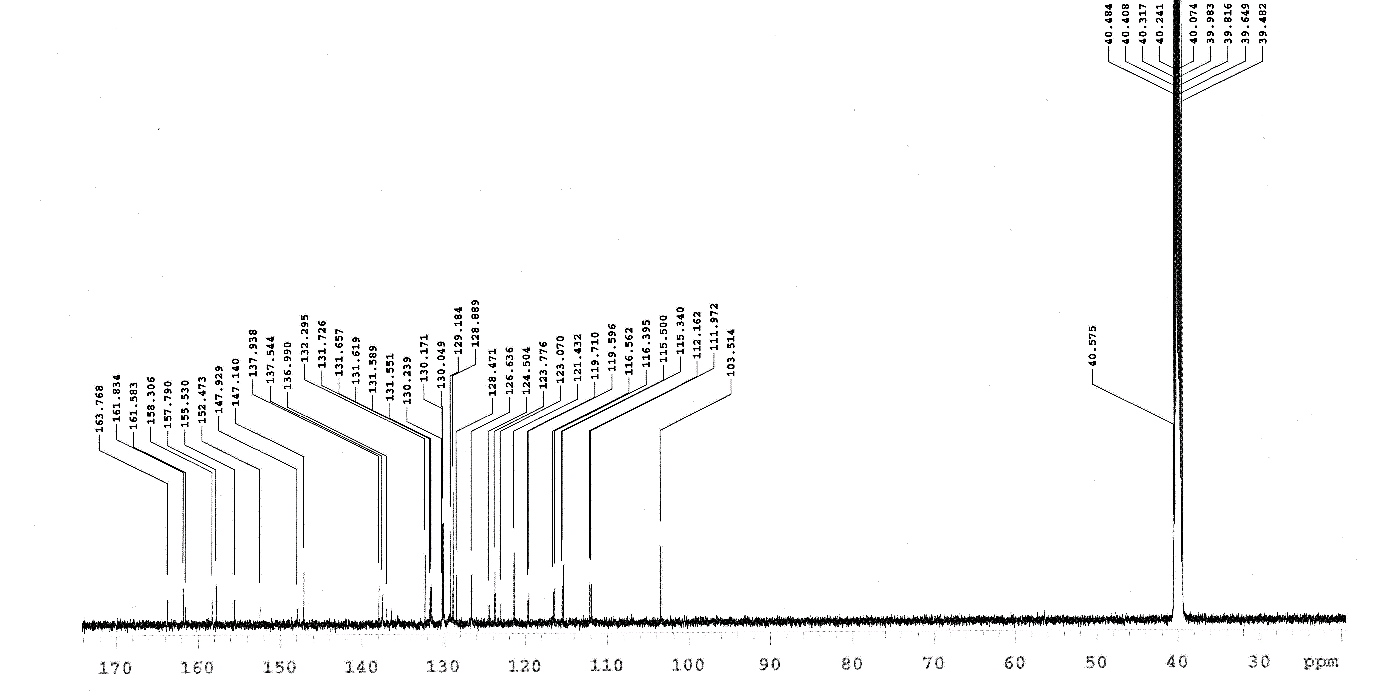


**Figure S1.22:** ^1^H- and ^13^C-NMR spectra of **10g** in DMSO-*d_6_* at 500 MHz and 125 MHz, respectively.


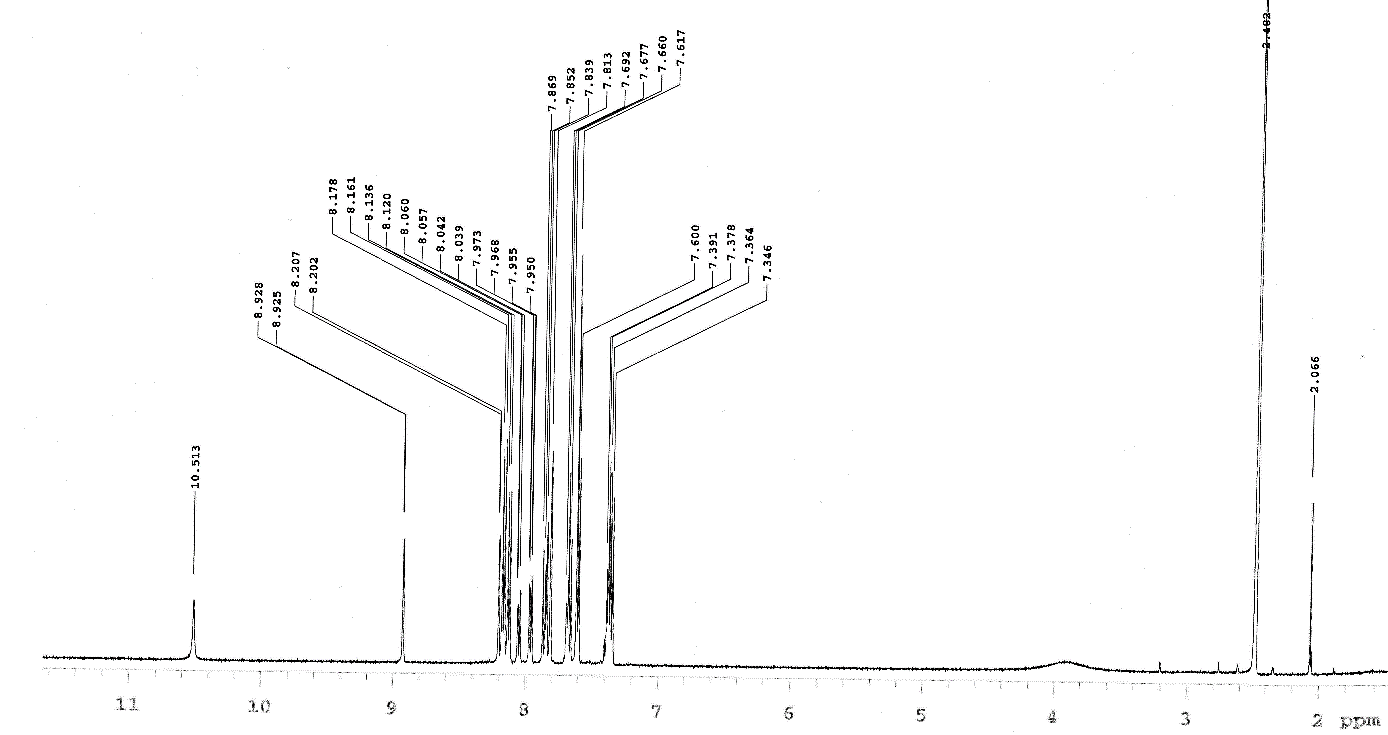

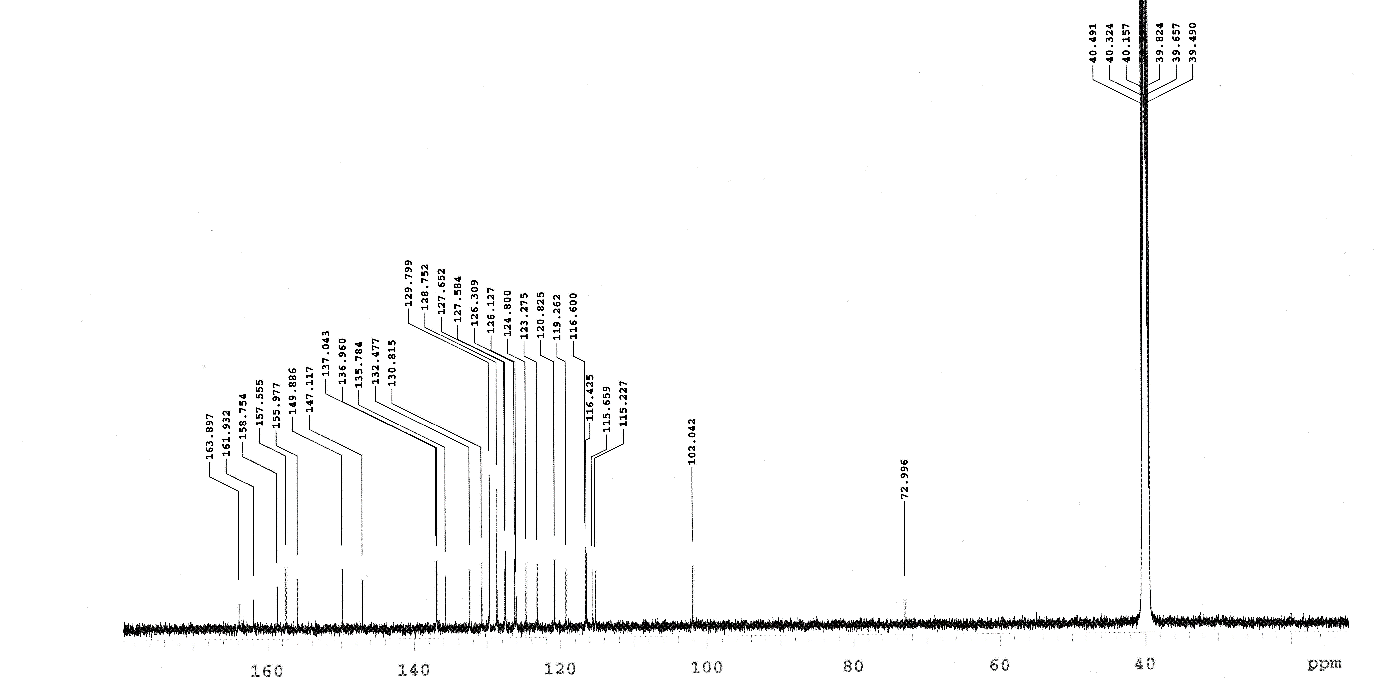


**Figure S1.23:** ^1^H- and ^13^C-NMR spectra of **10h** in DMSO-*d_6_* at 500 MHz and 125 MHz, respectively.


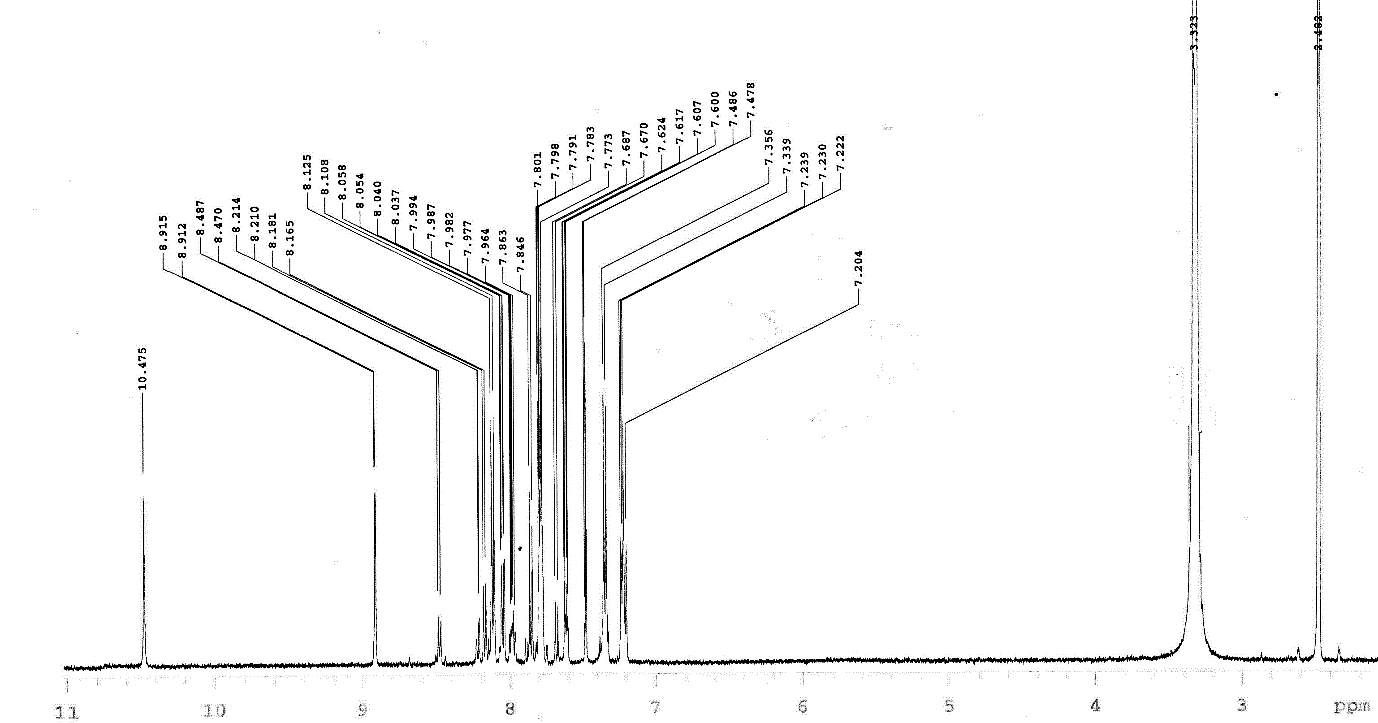

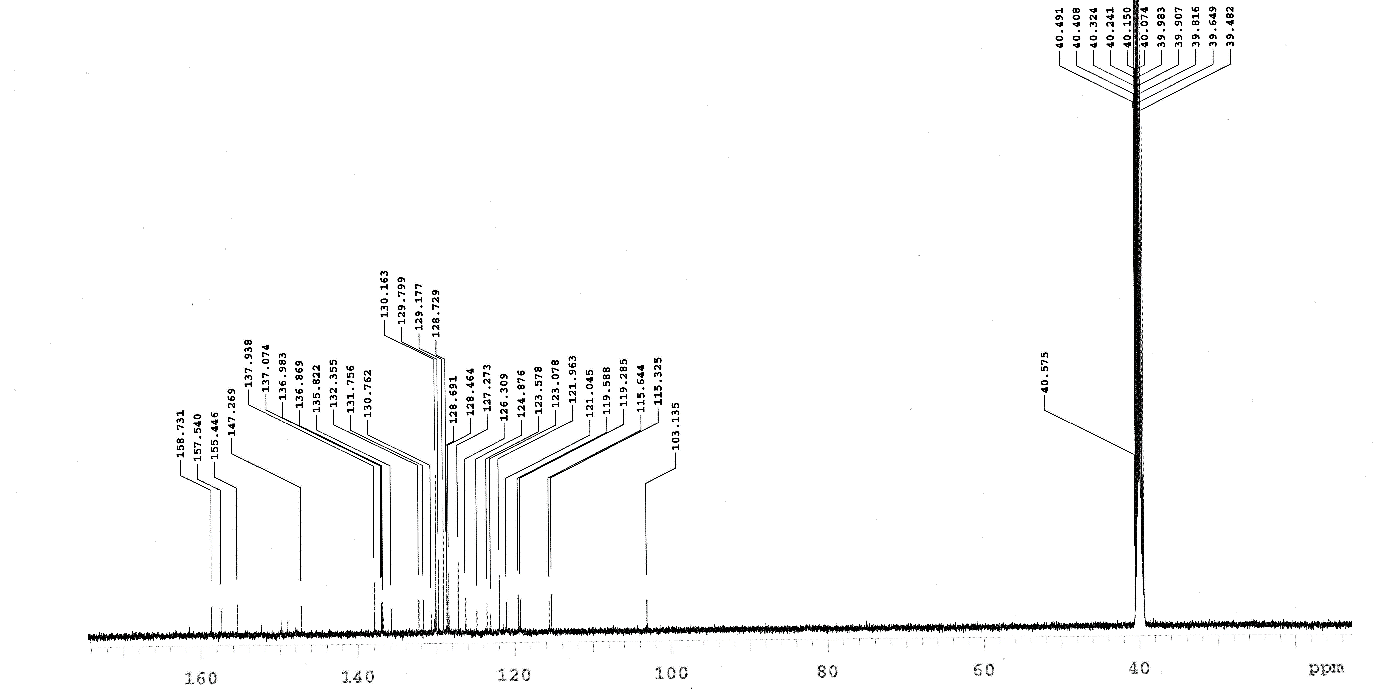


**Figure S1.24:** ^1^H- and ^13^C-NMR spectra of **10i** in DMSO-*d_6_* at 500 MHz and 125 MHz, respectively.

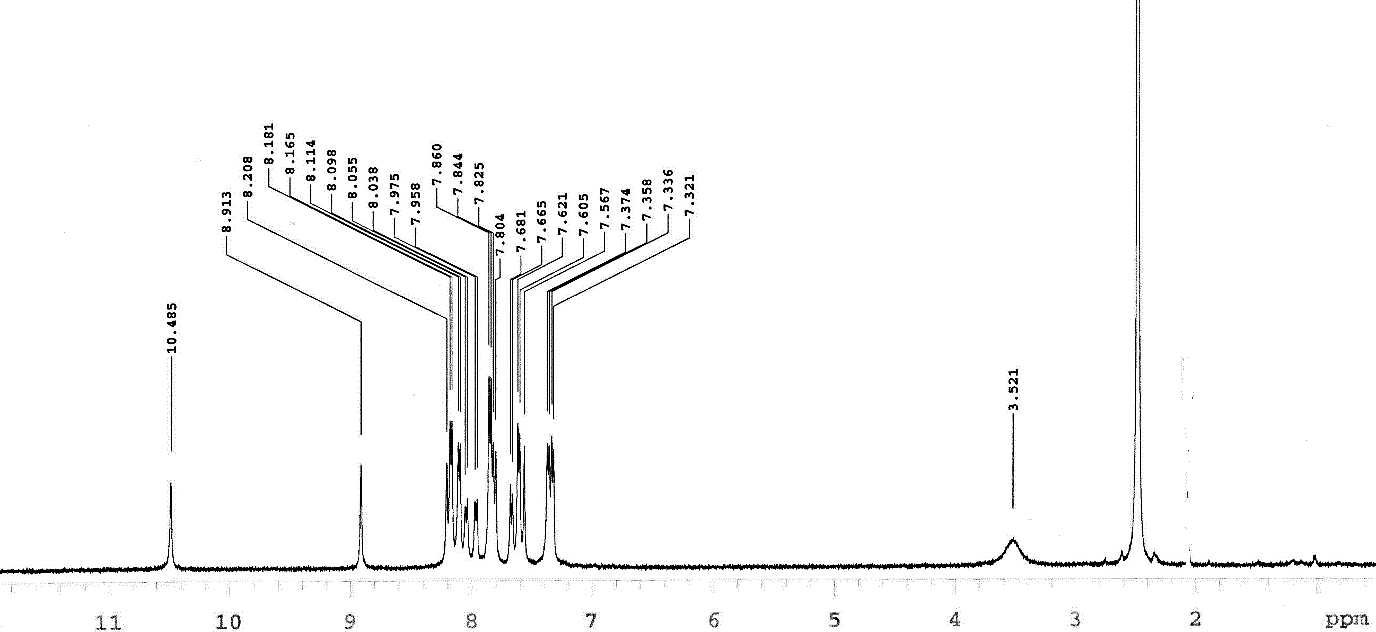


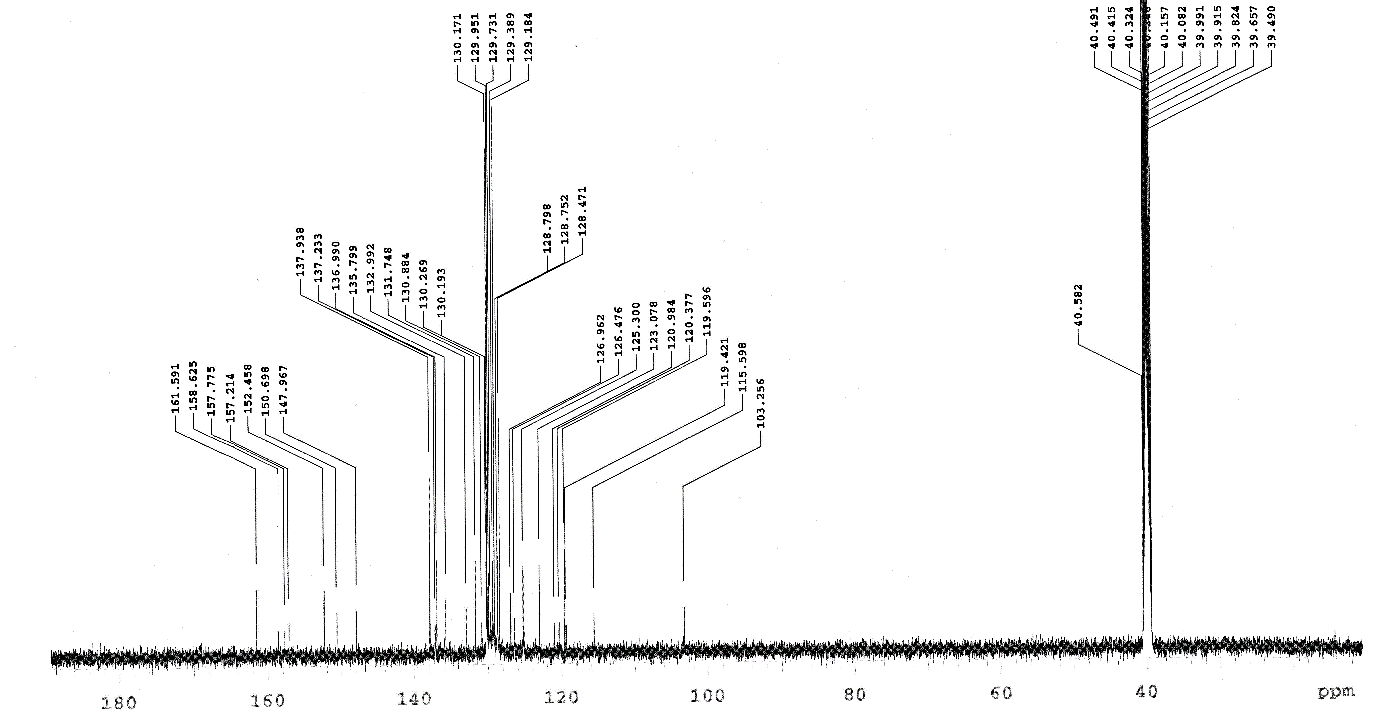


**Figure S1.25:** ^1^H- and ^13^C-NMR spectra of **10j** in DMSO-*d_6_* at 500 MHz and 125 MHz, respectively.
